# Rain Amplification of Persistent Organic Pollutants

**DOI:** 10.1021/acs.est.1c03295

**Published:** 2021-09-23

**Authors:** Gemma Casas, Alícia Martinez-Varela, Maria Vila-Costa, Begoña Jiménez, Jordi Dachs

**Affiliations:** † Institute of Environmental Assessment and Water Research, Spanish National Research Council (IDAEA-CSIC), Barcelona, Catalonia 08034, Spain; ‡ Department of Instrumental Analysis and Environmental Chemistry, Institute of Organic Chemistry, Spanish National Research Council (IQOG-CSIC), Madrid 28006, Spain

**Keywords:** wet deposition, snow, scavenging, amplification, Antarctica, PFASs, OPEs, PAHs, PCBs, PBDEs

## Abstract

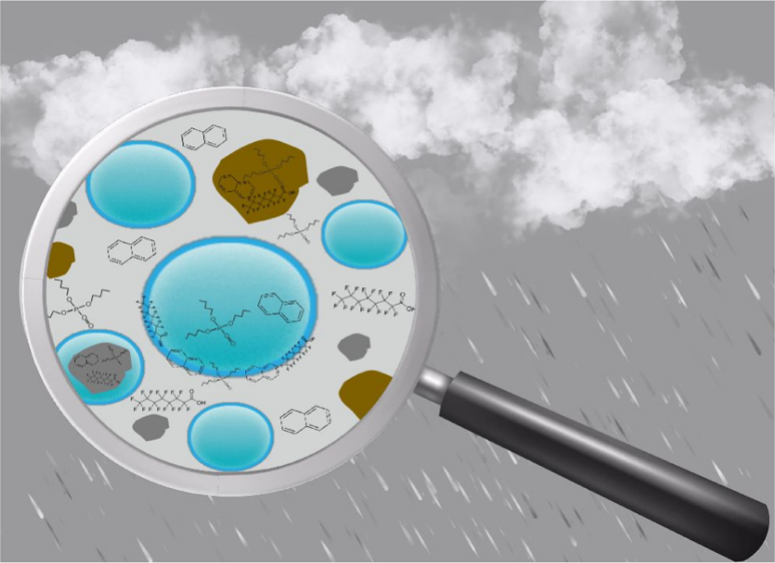

Scavenging of gas- and aerosol-phase organic pollutants by rain is an efficient wet deposition mechanism of organic pollutants. However, whereas snow has been identified as a key amplification mechanism of fugacities in cold environments, rain has received less attention in terms of amplification of organic pollutants. In this work, we provide new measurements of concentrations of perfluoroalkyl substances (PFAS), organophosphate esters (OPEs), and polycyclic aromatic hydrocarbons (PAHs) in rain from Antarctica, showing high scavenging ratios. Furthermore, a meta-analysis of previously published concentrations in air and rain was performed, with 46 works covering different climatic regions and a wide range of chemical classes, including PFAS, OPEs, PAHs, polychlorinated biphenyls and organochlorine compounds, polybromodiphenyl ethers, and dioxins. The rain–aerosol (*K*_RP_) and rain–gas (*K*_RG_) partition constants averaged 10^5.5^ and 10^4.1^, respectively, but showed large variability. The high field-derived values of *K*_RG_ are consistent with adsorption onto the raindrops as a scavenging mechanism, in addition to gas–water absorption. The amplification of fugacities by rain deposition was up to 3 orders of magnitude for all chemical classes and was comparable to that due to snow. The amplification of concentrations and fugacities by rain underscores its relevance, explaining the occurrence of organic pollutants in environments across different climatic regions.

## Abstract 2

Rain scavenging of atmospheric organic pollutants has a high capacity to amplify concentrations in aquatic and terrestrial environments across climatic regions.

## Introduction

Persistent organic pollutants (POPs) have the potential for long-range atmospheric transport (LRAT) from source to remote regions due to their persistence, semivolatility, or transference to the atmosphere with sea-spray aerosols.^1−3^ The study of LRAT and atmospheric deposition has been central in previous assessments of the occurrence of POPs at regional and global scales. Some of these mechanisms were previously described, such as cold trapping,^4^ enhanced deposition due to the biological pump,^5,6^ degradation pump enhanced deposition,^7,8^ temperature and biological pump-driven grasshopping over the oceans,^4,9^ and retardation of grasshopping due to sorption to soil and vegetation organic matter.^10,11^ Many of these transport mechanisms are fugacity-driven diffusive fluxes between the air and the receiving surface (water, soils, and vegetation). Conversely, wet deposition by rain and snow is independent of the fugacity gradient between the air and surface but is dependent on the capacity of rain and snow to scavenge atmospheric pollutants.^12−14^ These deposition processes can lead to higher concentrations of organic pollutants in water and soils than those derived from air-surface partitioning only, a process named “amplification.” However, the amplification of organic pollutants has received little attention. Previous works focused on the role of snow deposition amplifying the concentrations of POPs in soils and seawater.^15−19^ Comparatively, the role of rain deposition has received less attention.

Wet deposition by rain or snow^12^ scavenges POPs found in the gas and aerosol phases very efficiently and can amplify concentrations of POPs in other environmental compartments, such as in seawater^18−21^ or soils.^22−25^ This process has been proven to be especially effective for snow scavenging and is one of the main entries of POPs in cold regions, such as high-mountain and polar regions.^18,26−28^ Partition toward the snowflakes is favored at low temperatures. This together with the high specific surface area of snow, makes this deposition process crucial to understand the occurrence of a large variety of POPs in polar regions.^17−19,26,27,29^ A meta-analysis of the snow–air partition constants (*K*_SA_), estimated as the ratio of POP concentrations in snow and air, from previously reported simultaneous field measurements, showed that snow amplification was relevant for diverse families of POPs, independent of their volatility.^18^ The same work showed that seawater–air fugacity ratios of polychlorinated biphenyls (PCBs) were highly correlated with the product of *K*_SA_ and the dimensionless Henry’s law constant (*H*′), a measure of snow amplification of fugacity. Therefore, coastal seawater mirrored the PCBs in snow due to snowmelt releasing POPs. This and other observations of the occurrence of POPs provide the field evidence that pollution in the environment is underpredicted when considering air-surface diffusive partitioning only.

Rain also scavenges atmospheric pollutants found in the gas and aerosol phases, but the role of rain deposition, occurring under various climatic regimes, remains unclear, especially in terms of amplification of concentrations and fugacities. Snow and rain deposition have been compared previously, suggesting that snow is significantly more efficient than rain when collecting POPs from the atmosphere.^26^ Nowadays, the occurrence of legacy and emerging POPs have been reported in rain from diverse climatic regions, with what has become a remarkable body of field evidence of the role of rain as a deposition process.^30−74^

The wet deposition flux (*F*_WD_, pg m^–2^ d^–1^) is given by
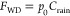
1where *p*_0_ (m d^–1^) is the precipitation depth per day and *C*_rain_ (pg m^–3^) is the POP concentration in rain. It is useful to refer to the wet deposition flux for the raining period and for the atmospheric concentrations, which are often measured instead of concentrations in the rain. Then, the estimation of the wet deposition flux is given by
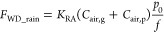
2where *C*_air,p_ is the concentration in the particulate phase of air, *C*_air,G_ is the concentration in the gas phase, and f is the fractional occurrence of rain. The rain–air partition constant (*K*_RA_), also called the water/air scavenging ratio or washout ratio, is given by^33^
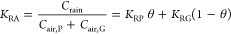
3where *K*_RG_ and *K*_RP_ are the rain–air partition constants for the gas phase and particulate phase, respectively. θ (dimensionless) is the fraction of aerosol-bound POPs to the total atmospheric POP concentration (*C*_air,P_/*C*_air,P_ + *C*_air,G_). These dimensionless partition constants can be estimated by
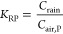
4
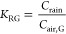
5*K*_RA_ is thus the scavenging ratio including both the gas- and particulate-phase concentrations in air, while *K*_RP_ and *K*_RG_ only consider the scavenging of aerosol- or gas-phase POPs, respectively.

The values of *K*_RP_ depend, in a complex manner, on meteorology, aerosol characteristics, and chemical properties and have generally been determined empirically. Field derived values are highly variable with a mean value^13^ of 2 × 10^5^. Conversely, *K*_RG_ depends on the raindrop-air diffusive partitioning and the POP adsorption on the raindrop surface from the gas phase. Thus,
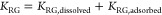
6

Therefore, *K*_RG_ is the sum of the absorption and adsorption terms (*K*_RG_,_dissolved_ and *K*_RG,adsorbed_). *K*_RG_,_dissolved_ is given by the inverse of the dimensionless Henry’s law constant (1/*H*′). On the other hand, *K*_RG,adsorbed_ depends on the water interface-air partition constant and the size distribution of rain drops.^13,75^ The second term in eq 6 has been suggested to be important for polycyclic aromatic hydrocarbons (PAHs), dioxins, and furans (PCDD/Fs) but not for PCBs.^13,75^ When the adsorption to raindrops is important, then it is possible that there is an amplification of gas-phase POPs in rain.

The objectives of this work are (i) to report a meta-analysis of the rain–air partitioning of POPs previously reported in the literature, with a contribution of a new data set of perfluoroalkyl substances (PFAS), organophosphate ester (OPEs), and PAHs in the Antarctic rain, (ii) to assess the potential for rain amplification for different legacies and emerging POPs, and (iii) to compare rain and snow amplification of POPs.

## Materials and Methods

### Site Description and Sampling

The samples for this study were collected during two sampling campaigns. The first campaign was carried out in Deception Island (62°58′S 60°39′W) during the Antarctic summer 2016–2017, while the other one was in Livingston Island (62°36′S 60°30′O) during the Antarctic summer 2017–2018. These two islands are located in the South Shetland Archipelago (Figure S1) in the Antarctic Peninsula. In this area, periods with temperatures above freezing during summer allow rain to fall.^76^ The samples for PFAS analysis were collected in both islands; however, the samples for OPEs and PAHs were collected only in Livingston Island.

The rain samples were collected with a stainless steel tray, from which 2 L was poured into Teflon bottles for the analysis of OPEs and PAHs (*n* = 10), and 2 L was poured into polypropylene bottles for the analysis of PFAS (*n* = 21). After 30–60 min of sample collection, the samples were filtered through precombusted GF/F glass fiber filters (47 mm diameter, Whatman 0.7 μm mesh size) before passing through their specific cartridges at the Antarctic research station’s laboratory. The PFAS extraction was based on a solid-phase extraction (SPE) procedure using Oasis WAX cartridges (6 cm^3^, 150 mg; Waters).^77^ On the other hand, OPEs and PAHs were extracted using an SPE procedure with Oasis hydrophilic–lipophilic balance cartridges (6 cm^3^, 200 mg; Waters) by following an established methodology^78,79^ with some modifications. Samples were spiked with recovery standards before the SPE (Table S1). Aerosol samples were collected (Figure S1) using a high volume air sampler (MCV, Collbató, Spain) operating at 40 m^3^ h^–1^. The air was drawn through a precombusted and preweighed quartz microfiber filter (QM-A; Whatman, 8–10 inches) to collect aerosol-bound compounds (total suspended particle). Twenty aerosol samples were analyzed for PFAS and 6 aerosol samples were analyzed for OPEs and PAHs. All the samples/cartridges were stored at −20 °C, after the sampling, until analysis in an ultraclean laboratory in Barcelona.

The procedures followed for the extraction, identification, and quantification of PFAS, OPEs, and PAHs are described in Annex S1 in Supporting Information. Quality assurance and quality control are reported in Annex S2 in Supporting Information. Recoveries and limits of detection are summarized in Tables S2 and S3 in Supporting Information.

### Criteria for Meta-Analysis of Rain–Air Partition Constants

**Figure 1:**
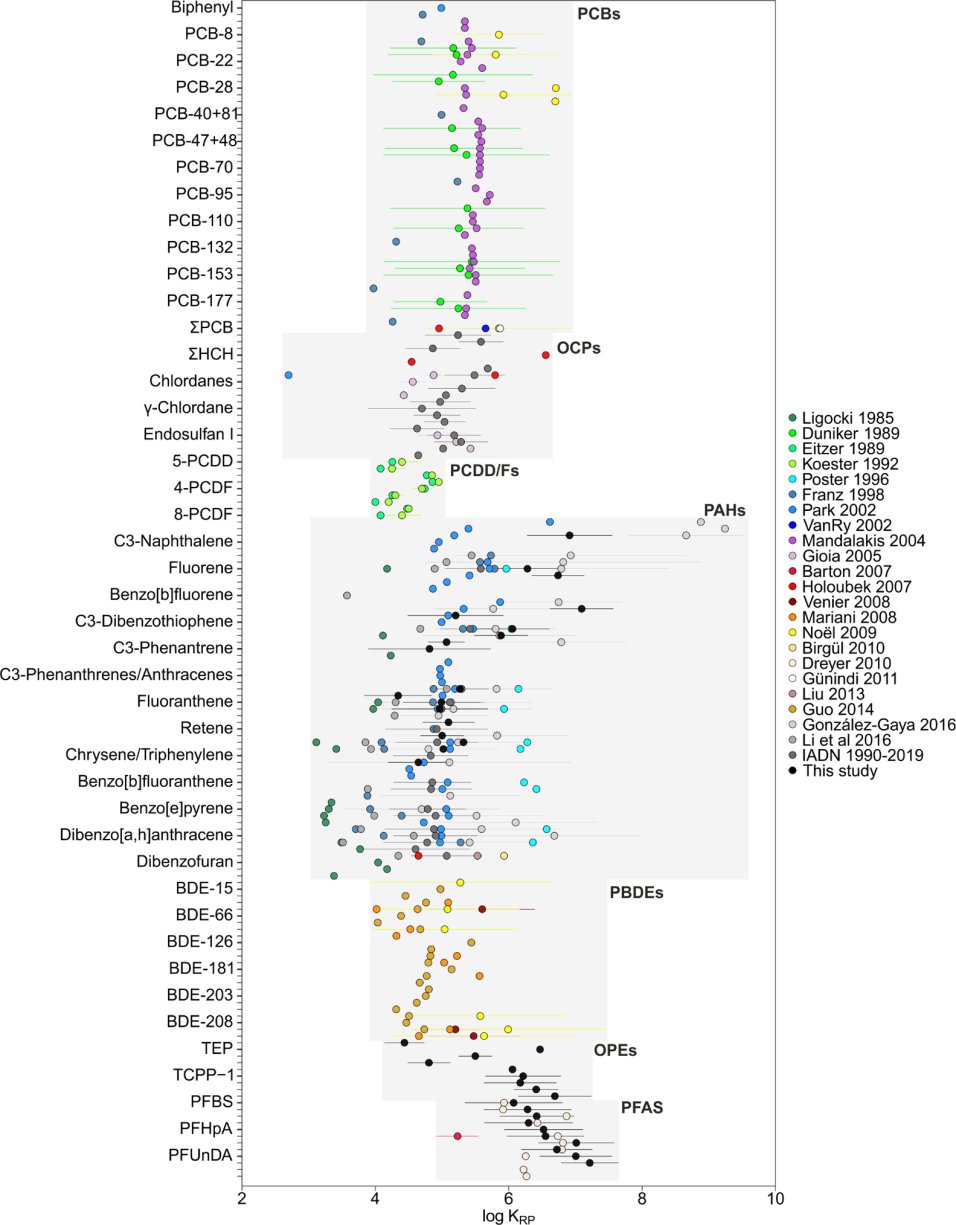
Meta-analysis of rain–air particulate partition constants (*K*_RP_) for various families of organic pollutants. The results shown are the mean and the standard deviation of log *K*_RP_.

**Figure 2:**
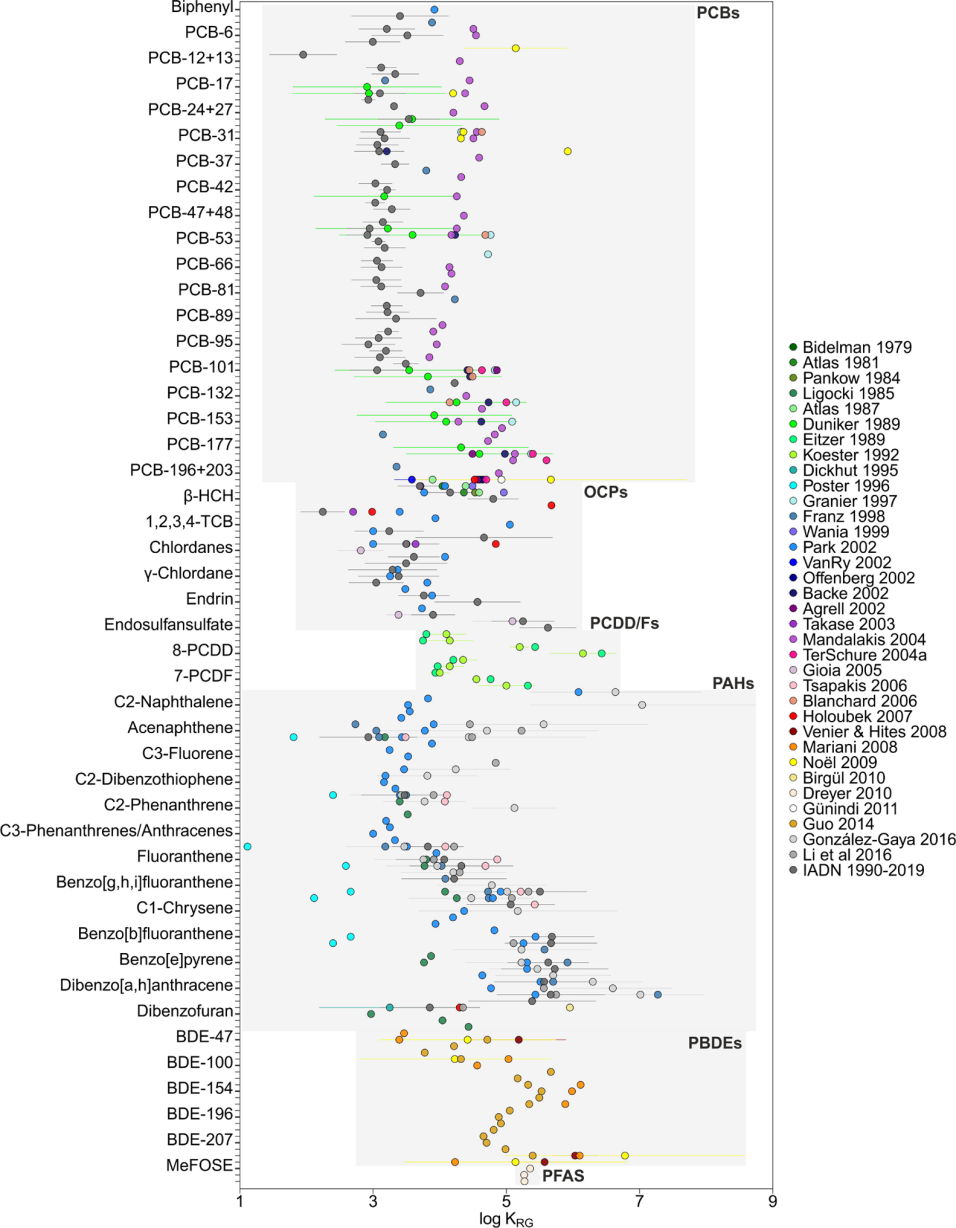
Meta-analysis of rain–gas partition constants (*K*_RG_) for various families of organic pollutants. The results shown are the mean and the standard deviation of log *K*_RG_.

**Figure 3:**
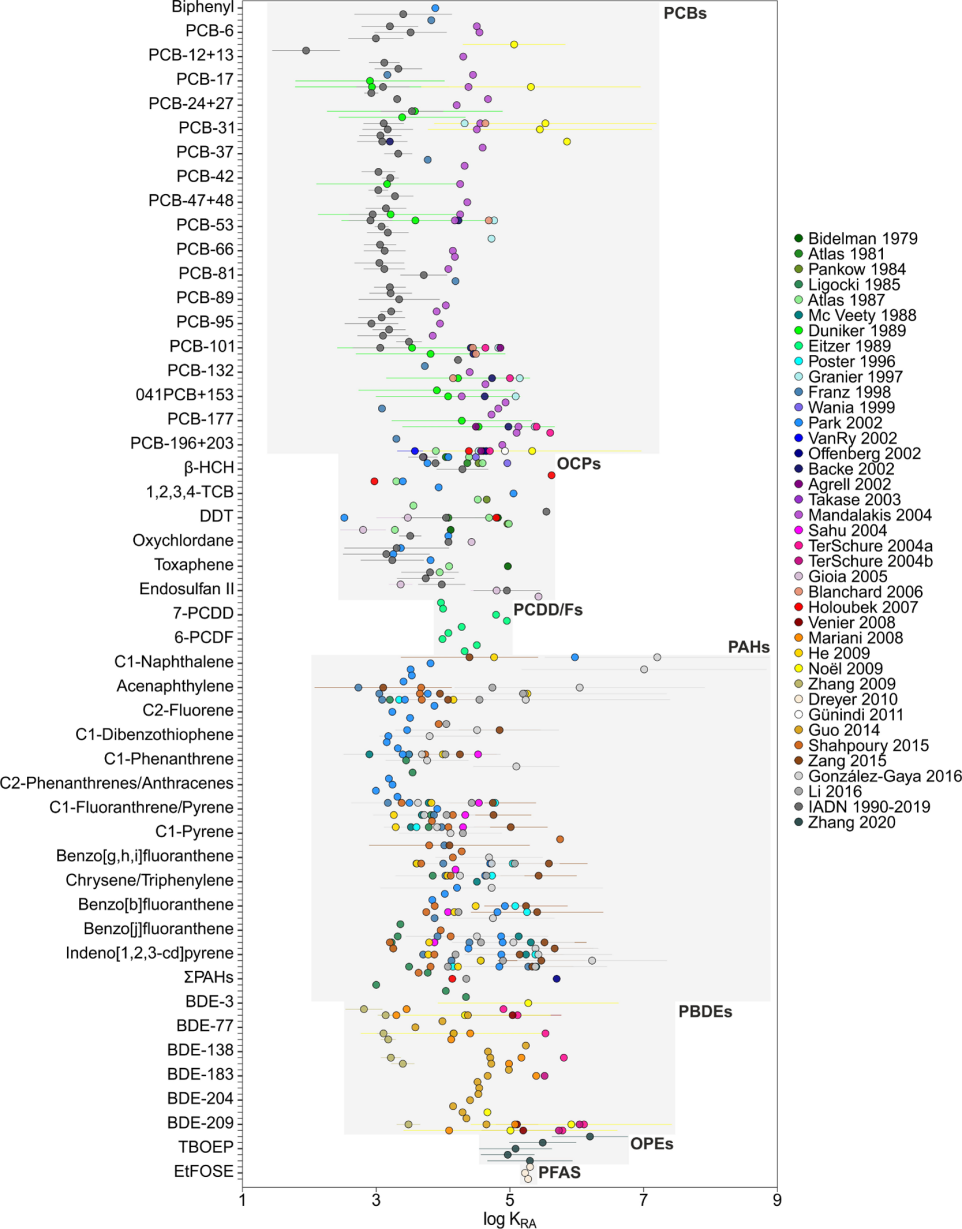
Meta-analysis of rain–air (particulate + gas phase) partition constants (*K*_RA_) for various families of organic pollutants. The results shown are the mean and the standard deviation of log *K*_RA_.

**Figure 4:**
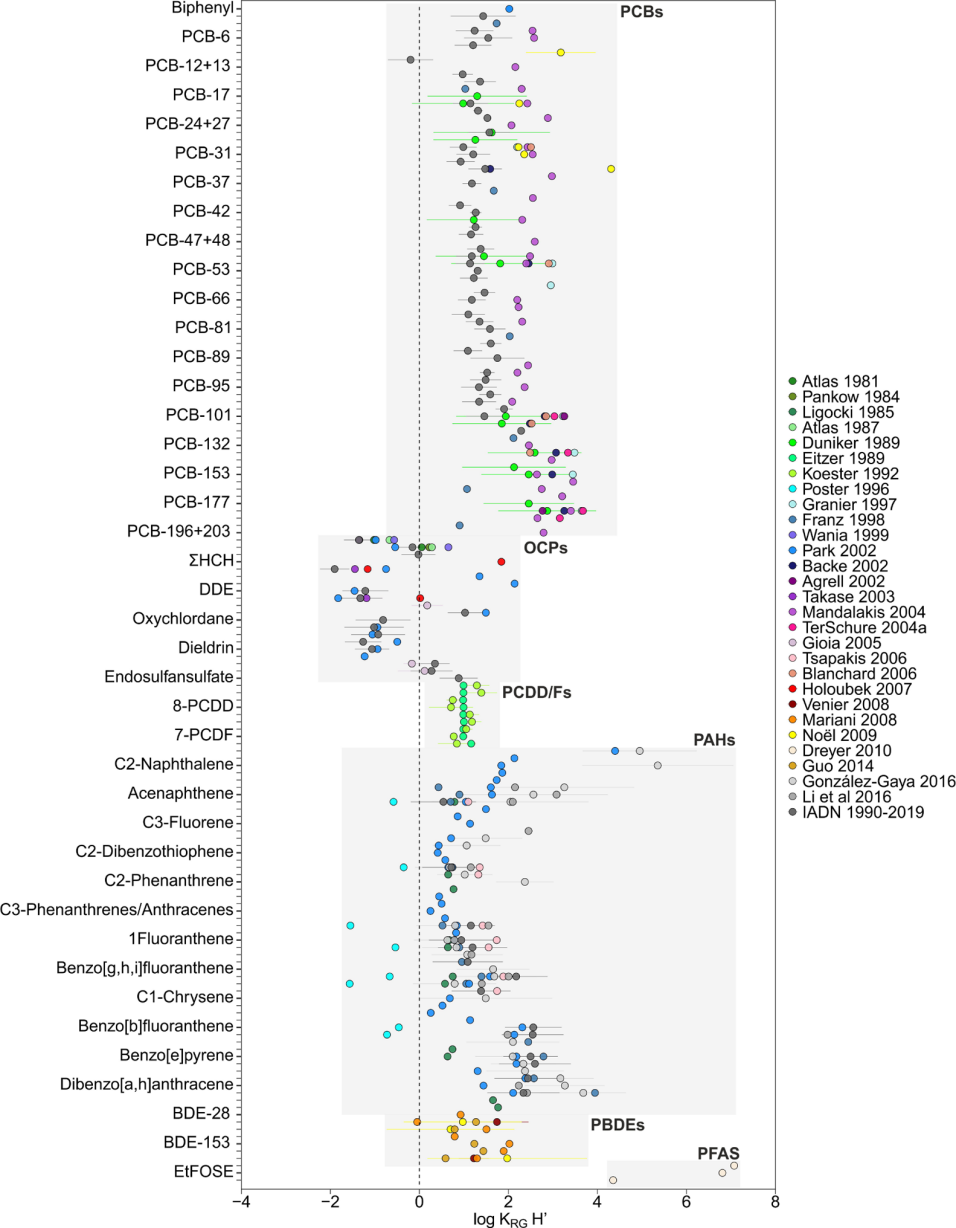
Predicted rain amplification of the fugacity ratio between rain and air given by the product of the rain–air (gas phase) partition constant and the dimensionless Henry’s law constant (*f*_w_/*f*_G_ = *K*_RG_*H*′) for various families of organic pollutants. The results shown are the mean and the standard deviation of log *K*_RG_*H*′.

**Figure 5:**
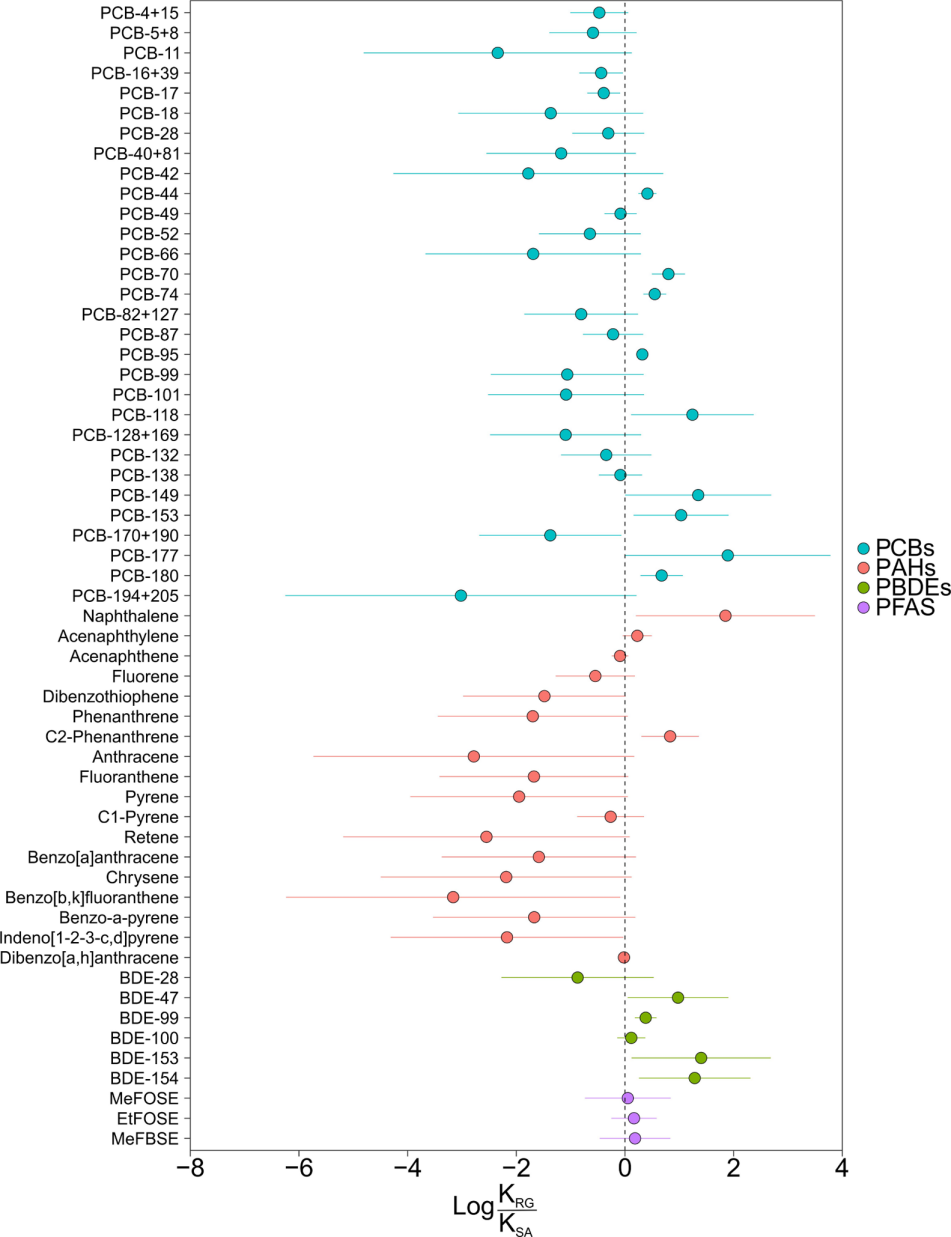
Comparison of amplification of organic pollutants by rain and snow. The results show the mean of *K*_RG_/*K*_SA_ and its error estimated using the uncertainty propagation approach (Annex S3).

We reviewed the previous reports of organic pollutants in rain and air (particulate and gas phase), and a total of 45 publications were found and used for this study.^30−74^ There are additional reports of field concentrations of organic pollutants in rain,^80−101^ but they lack concurrently measured atmospheric concentrations, or data were not given in the manuscript, and thus rain–air partition constants could not be estimated. Table S4 summarizes the data set contained in the 45 publications used for the partition constants. With the new data reported for PFAS, OPEs, and PAHs in this study, a total of 46 data sets were used in this meta-analysis. The meta-analysis was made for the three rain–air partition constants (eqs 3–5). In some of the previous studies, one, two, or three of the coefficients, *K*_RP_, *K*_RG_, and *K*_RA_ were already provided. For the other works reporting concentrations, we calculated the respective partition constants by following eqs 3–5. We were not able to perform the meta-analysis with all data sets for the three different coefficients as some works only reported concentrations for the particulate, or gas phase, or total atmospheric concentration. In the case of PCBs and hexachlorocyclohexane (HCH), if the authors provided only the concentrations in the gas phase, both *K*_RG_ and *K*_RA_ were estimated, as it is well known that these POPs are mostly found in the gas phase (low θ value in eq 3). These data were used to derive Figures 1, 2, 3, 4 and S5–S9 and are reported in Tables S4–S11. The results shown in these figures are the mean and the standard deviation of the partition constants for each compound and each data set. The standard deviation is not given when the original work provided only one value of the coefficients for each compound or reported only the mean (details in Table S4). We focused on rain–air partition coefficients as reported directly from chemical measurements. We did not consider estimates of these partition constants from models, even if these were partly based on measurements. For the estimation of *K*_RG_*H*′ (amplification potential), we used *H*′ values at 298.15 K (Table S8). All the calculations, statistics, and plots for the meta-analysis were performed using R Studio 1.4 (Figures 1, 2, 3, 4, 5, S1–S9, Tables S12).

## Results and Discussion

### Concentrations of PFAS, OPEs, and PAHs in Rain and Aerosols from Maritime Antarctica

The concentrations of the individual PFAS, OPEs, and PAHs measured in the rain and aerosol samples from Deception and Livingston Islands (the Antarctic Peninsula) are summarized in Tables S9–S11 (Supporting Information) and shown in Figures S2–S4. The average and range concentrations of ∑PFASs in rain and aerosol samples from Deception Island were 3,600 (660–7,600) pg L^–1^ and 0.13 (0.056–0.32) pg m^–3^, respectively. The average and range concentrations of ∑PFAS in rain and aerosol samples from Livingston Island were 3,600 (400-8,400) pg L^–1^ and 0.19 (0.0067–0.71) pg m^–3^, respectively. To the best of our knowledge, this is the first report of POP concentrations in rain from Antarctica. These concentrations in rain were 1 order of magnitude lower than those reported in Northern Germany.^65^ On the other hand, these PFAS concentrations in rain were similar to those measured in snow from Livingston Island.^19^ PFAS concentrations in aerosol samples were comparable to those reported previously at Livingston Island.^2^

The average and range concentrations of ∑OPEs in rain and aerosol samples from Livingston Island were 35,800 (9730–93,600) pg L^–1^ and 41.7 (13.3–28.3) pg m^–3^, respectively. The concentrations reported here for rain were 1 order of magnitude lower than those reported in Germany.^87,102^ The aerosol-phase concentrations of OPEs were comparable with those reported in the Western Antarctic Peninsula for 2014–2018,^103^ which ranged from 5.75 to 238 pg m^–3^.

The average concentrations of ∑PAHs in rain samples from Livingston Island averaged 9.05 (2.36–17.8) ng L^–1^ and concentrations of ∑PAHs in aerosol samples averaged 0.02 (0.005–0.03) ng m^–3^. These aerosol-phase concentrations from Livingston Island were comparable to the ∑PAH concentrations previously reported from the Antarctic and Southern Ocean atmosphere,^104^ where ∑PAH average concentrations from Livingston Island ranged from 0.03 to 0.09 ng m^–3^. The ∑PAH concentrations in rain for this study were significantly lower than those reported in rain during the Malaspina 2010 circumnavigation in the tropical and subtropical oceans.^71^

### Rain Scavenging of Aerosol-Bound POPs

Particle scavenging by rain is characterized by *K*_RP_. The efficiency of the washout of aerosols by rain depends on a number of factors, such as the aerosol and raindrop size distributions, among other factors.^13,75^ There are a number of reported measurements of *K*_RP_ for PCBs and PAHs, some of which have been used in modeling exercises for wet deposition.^13,36,44,45,50,58,62,66,105^ We compiled all the previous reports of simultaneous occurrence of organic pollutants in rain and aerosols, with 24 studies reporting concentrations of PCBs, PCDD/Fs, PAHs, organochlorine pesticides (OCPs), PBDEs, and PFAS, together with the new data set from this work for PAHs, OPEs, and PFAS from Antarctica. Figure 1 shows the comparison of *K*_RP_ from this meta-analysis. The compound-specific average log *K*_RP_ value ranged between 2.6 and 11.5; the mean value was 5.5.

The log *K*_RP_ value of PAHs ranged between 2.6 and 11.5, representing the highest variability. Among these, the values derived from studies by Ligocki et al. (1985)^33^ showed the most different values of *K*_RP_ compared with all the other studies, especially for the high molecular weight (MW) PAHs. Naphthalene and their methylated compounds showed the highest log *K*_RP_ average value, ranging from 4.9 to 11.5. With the exception of PAHs, for which there was a high variability of *K*_RP_, the other pollutant families showed similar *K*_RP_ values among the different studies. The log *K*_RP_ values of PCBs and OCPs presented similar ranges between studies, within the range 4.0–7.0 and 2.7–6.6, respectively. The log *K*_RP_ value of PCDD/Fs ranged between 4.0 and 5.0.^37,38^

As far as we know, the field-derived *K*_RP_ value of OPEs is reported here for the first time, with the log *K*_RP_ value ranging from 4.1 to 7.3. The log *K*_RP_ value of PBDEs ranged between 4.01 and 7.0.^49,50,59,60,62,63,68^ The *K*_RP_ value ranged between 3.6 and 8.8 for PFAS.^57,65^ We could only compare *K*_RP_ for various PFAS from two different studies that measured concentrations simultaneously in rain and aerosols. In addition, Barton et al. (2007)^57^ reported *K*_RP_ values for PFOA only. The *K*_RP_ values from previously reported concentrations^65,106^ were comparable with those measured here (Figure 1).

The aerosol type and the physical and chemical properties of the compound could influence the values of the scavenging ratios. OPEs and PFAS showed the highest average values for log *K*_RP_, together with naphthalene and some methylnaphthalenes. Overall, the *K*_RP_ values of the different chemicals showed high variability. Nevertheless, these were significantly correlated with *K*_OA_ and *H*′ (*K*_AW_), but correlations explained a small percentage of the variability (*r* = 0.39 or *r* = 0.2, respectively) (Figure S9 and Table S12). In addition, we compared *K*_RP_ for aerosols having different origins, by classifying the field studies between those performed in urban and continental areas and coastal and open oceans (Figure S5). A Tukey HSD test was carried out for performing pairwise comparison between the means of *K*_RP_ for different aerosol types, which showed significant differences between continental/urban areas and coastal/open ocean aerosols. Conversely, there were no significant differences between continental and urban areas and between coastal and open oceans. For modeling purposes, and for chemicals for which field-derived *K*_RP_ values (Figure 1) are not available, this meta-analysis shows that a value for log *K*_RP_ between 5 and 6 would be reasonable (Figure 1).

### Rain Scavenging of Gas-Phase POPs

Scavenging or washout of gas-phase organic pollutants by rain is characterized by *K*_RG_. There are also a number of reported *K*_RG_ field measurements for PCBs, OCPs, PAHs, PBDEs, and PCDD/Fs (Figure 2). Conversely, emerging pollutants have barely been measured concurrently in the gas phase and rain, with only one study reporting these for neutral PFAS (MeFOSE, EtFOSE, and MeFBSE).^65,106^Figure 2 shows the meta-analysis of *K*_RG_ with data from 35 studies. The compound-specific average log *K*_RG_ value ranged between 1.1 and 9.6; the mean value was 4.1.

PAHs showed a high variability of log *K*_RG_ (ranging between 1.1 and 7.3), while PCBs and OCPs showed similar *K*_RG_ values among the different studies. In the case of PAHs, the *K*_RG_ value derived from studies by Poster and Baker (1996)^40^ presented the lowest values in comparison with the rest of the studies. Generally, *K*_RG_ increases as the number of aromatic rings increases, with the exception of naphthalene and alkyl-naphthalene (3.4–9.6), which have similar *K*_RG_ to high molecular weight (MW) PAHs, such as indeno[1,2,3-*cd*]pyrene, dibenzo[*a*,*h*]anthracene, and benzo[*g*,*h*,*i*]perylene, ranging from 3.8 to 7.9. There is the possibility that there is some redissolution from aerosols to the rainwater dissolved phase, which would cause a sampling artifact explaining the high *K*_RG_ values observed for some chemicals, but this would be not consistent with the strong association of high MW PAHs with aerosol soot carbon. PCBs and OCPs presented similar compound-specific log *K*_RG_ values among the different studies, ranging between 1.6–7.1 and 1.4–6.2, respectively. The log *K*_RG_ value of PCDD/Fs ranged between 3.8 and 6.5. The log *K*_RG_ values of PBDEs and neutral PFAS ranged from 3.2 to 8.1 and 5.3 to 5.4, respectively.

*K*_RG_ depends on the raindrop-air diffusive partitioning and the POP adsorption on the raindrop surface from the gas phase. *K*_RG,dissolved_ equals the inverse of *H*′, and thus, knowing the field-derived *K*_RG_, we could estimate *K*_RG,adsorbed_ (eq 6). Figure S6 shows that the *K*_RG,adsorbed_ value ranged between 1.1 and 9.6, with a mean of 4.1. Only for *K*_RG_ for PAHs reported by Poster and Baker, *K*_RG,adsorbed_ was negligible. For all other data sets and chemicals, adsorption on the raindrop is predicted to be not only important but also to dominate as a scavenging mechanism of gas-phase POPs from the atmosphere. This suggests that the common modeling practice of estimating *K*_RG_ as 1/*H*′ induces an underestimation by several orders of magnitude of the importance of rain deposition of POPs. In fact, *K*_RG_ showed a weak correlation with *H*′ (spearman *r* = −0.156, *n* = 498, *p* < 0.001) (Figure S8 and Table S12). For modeling *K*_RG_ of chemicals other than those shown in Figure 2, a mean value of 10^4.5^ can be used but with high uncertainty.

### Amplification of POPs by Wet Deposition

The overall importance of rain scavenging of both gas- and aerosol-phase POPs is characterized by *K*_RA_ (Figure 3), which can only be reported for those studies providing the concentration in both the aerosol and gas phases separately (39 studies, Table S4). The log *K*_RA_ value ranged between 1.2 and 10.1, with such large variability observed mainly for PAHs. Such large variability of *K*_RP_, *K*_RG_, and *K*_RA_ for PAHs is surprising. This is not due to limitations or difficulties in their chemical analysis as PAHs are at atmospheric concentrations several orders of magnitude higher than other POPs, such as PCBs, PCDDs/Fs, or OPEs. A characteristic of low MW PAHs is that they degrade in the air and water by photodegradation and biodegradation.^8,107^ High MW PAHs are protected by association, adsorption, or incorporation into the particles or in the black carbon.^112,113^ Such degradation in rain could be a reason explaining such large variability of the rain–air partition constants for these chemicals. Confirmation of this hypothesis would require further work. Previously, it was shown that snow amplification of PAHs was reduced due to degradation,^17^ and it could occur similarly for rain amplification. Log *K*_RA_ correlations with the chemical properties explained a small fraction of the variability for most chemical classes (Figure S7 and Table S12).

The amplification potential of POP fugacity by rain is given by
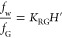
where *f*_W_ and *f*_G_ are the POP fugacity in the deposited water and gas phases, respectively. Figure 4 shows the log *K*_RG_*H*′ values of PCBs, OCPs, PAHs, PCDD/Fs, PBDEs, and three neutral PFAS. With the exception of some studies, there is an amplification of concentration in rain for all compounds, which is maximum for naphthalene, alkyl-naphthalenes, and neutral PFASs.

The amplification of fugacities by rain can occur when adsorption to raindrops is a significant process. This is the case for most POPs (Figure S6). Thus, there is a generalized amplification of the fugacities for all POPs (Figure 4), which surprisingly is especially more relevant for the more volatile chemicals, which can be as high as 6 orders of magnitude. For other POPs, the amplification potential is still important but generally below 3 orders of magnitude.

### Rain and Snow Amplification of POPs

The investigation on which of the two wet deposition processes (snow or rain) is the most effective in scavenging organic compounds has been a recurrent topic in the “fate and transport” field,^12,16,26,28,108^ but this comparison was often focused on predictions from models rather than field-derived assessments. Furthermore, snow has received more attention as an amplification mechanism for POPs than rain. This is especially relevant in polar environments, even though rainfall occurrence is predicted to increase in the coming decades.^76^ The meta-analysis performed here allows for calculating the ratio between *K*_RG_ versus de *K*_SA_ (the snow-air partition coefficient) from the field-derived data. For such a comparison, we use *K*_SA_ as estimated in a companion meta-analysis reported elsewhere.^18^

Figure 5 shows log *K*_RG_/*K*_SA_ for comparative purposes. Such comparisons could be done for PCBs, PAHs, neutral PFAS, and PBDEs. In addition to the mean, we calculated the error using the uncertainty propagation approach (Annex S3). The log *K*_RG_/*K*_SA_ value ranged between −3.16 and 1.9, presenting negative values of log *K*_RG_/*K*_SA_ for most PAHs and some PCB congeners, while positive values of log *K*_RG_/*K*_SA_ for PBDEs, low MW PAHs, and some PCB congeners. Therefore, the fieldwork carried out during the last four decades shows that snow and rain amplification of POPs are of comparable magnitude, with differences that are compound specific.

Snow deposition is limited to cold regions and/or cold periods of time, while rain precipitation occurs widely for different seasons and across climatic regions. Furthermore, there are observations that concentrations in rivers increase after strong rain events.^60,82,83,109−111^ Such large concentrations would be driven by the amplification of POPs by rain and the focusing of water in rivers from the watershed. Future work should be focused on studying the role of wet deposition on the cycle and occurrence of organic pollutants, especially in terms of its spatial and temporal dynamics, and extending this assessment to chemicals of emerging concern. Climate change induces a perturbation of the magnitude and frequency of precipitation events, which should be considered as a potential factor influencing the POP dynamics and amplification under a scenario of global environmental change.

## Supporting Information Available

Analytical procedures, quality assurance/quality control, sampling location for the rain and aerosol samples, PFAS concentrations in aerosols and rain samples from Deception and Livingston Islands, OPEs and PAHs concentrations in aerosols and rain samples from Livingston Island, Meta-analysis of rain-air particulate partition constants, Pearson’s correlations, details of target, recovery, and internal standards for PFAS, OPEs, and PAHs, PFAS, OPE, and PAH sample recoveries of recovery standards, details of limits of detection for PFAS and OPEs for rain and aerosol samples from Deception and Livingston Islands, data used in the meta-analysis of rain-air partition constants, K_RP_ mean, K_RG_ mean, and K_RA_ mean for each compound and each data set, dimensionless Henry’s law constant values, and Pearson’s correlations between log K_RA_, log K_RG_, and log K_RP_ with log K_aw_, log K_oa_, and log K_ow_ (PDF)
