# Supplementary material for: Rain Amplification of Persistent Organic Pollutants
Source: Environ Sci Technol. 2021 Sep 23;55(19):12961–72. doi: 10.1021/acs.est.1c03295 (PMC8495897; doi:10.1021/acs.est.1c03295)
Supplement: Supplementary file 1 — es1c03295_si_001.pdf [file es1c03295_si_001.pdf]

Supporting Information

Rain Amplification of Persistent Organic Pollutants

Gemma Casas[1,2], Alícia Martinez-Varela[1], Maria Vila-Costa[1], Begoña Jiménez[2], and Jordi Dachs[1*]

[1]Institute of Environmental Assessment and Water Research, Spanish National Research Council (IDAEA-CSIC), Barcelona, Catalonia 08034, Spain

[2]Department of Instrumental Analysis and Environmental Chemistry, Institute of Organic Chemistry, Spanish National Research Council (IQOG-CSIC), Madrid 28006, Spain

---

* Corresponding author email: jordi.dachs@idaea.csic.es

Summary

Pages: 59

Annexes: 3

Figures: 9

Tables: 12

Table of Contents

Annex S1. Analytical Procedures

Annex S2. Quality Assurance/Quality Control

Annex S3. Uncertainty error propagation estimation for K_{RG}/K_{SA}. Where se is the standard error.

Figure S1. Sampling location for the rain and aerosol samples analysed in this study.

Figure S2. PFAS concentrations in aerosol (pg m^{-3}, upper panel) and rain (pg L^{-1}, lower panel) samples from Deception and Livingston Island.

Figure S3. OPE concentrations in aerosol (pg m^{-3}, upper panel) and rain (pg L^{-1}, lower panel) samples from Livingston Island.

Figure S4. PAHs concentrations in aerosol (pg m^{-3}, upper panel) and rain (pg L^{-1}, lower panel) samples from Livingston Island.

Figure S5. Meta-analysis of rain-air particulate partition constants (K_{RP}) for various families of organic pollutants differentiating the type of aerosol (Continental, Coastal, Open ocean, Urban). The results shown are the mean and the standard deviation of log K_{RP}.

Figure S6. Meta-analysis of rain-air, gas phase adsorbed ($K_{RG,\ adsorbed}$) for various families of organic pollutants. $K_{RG,\ adsorbed}$ as given by Equation [6], $K_{RG} = K_{RG,dissolved} + K_{RG,adsorbed}$, where $K_{RG,dissolved}$ is 1/H'. The results shown are the mean and the standard deviation of log $K_{RG,adsorbed}$.

Figure S7. Pearson's correlations between log K_{RA} with log K_{aw}, log K_{oa}, log K_{ow}.

Figure S8. Pearson's correlations between log K_{RG} with log K_{aw}, log K_{oa}, log K_{ow}.

Figure S9. Pearson's correlations between log K_{RP} with log K_{aw}, log K_{oa}, log K_{ow}.

Table S1. Target, recovery and internal standards for PFAS (LC-MS/MS), OPEs (GC-MS/MS) and PAHs (GC-MS) analyzed in the present study.

Table S2. PFAS, OPEs and PAHs sample recoveries of recovery standards (%) for rain and aerosols samples.

Table S3. Limits of detection for PFAS, OPEs for rain and aerosols samples from Deception and Livingston s. The limits of detection (LODs) were defined as the mean concentration of field blanks plus three times the standard deviation of the blank response. For the analytes not detected in blanks, LOD were derived from the lowest standard in calibration curve.

Table S4. Information of the data used in the meta-analysis of rain-air partition constants

Table S5. K_{RP} mean for each compound and for each data set in the meta-analysis. The compound order is the same as in Figure 1.

Table S6. K_{RG} mean for each compound and for each data set in the meta-analysis. The compound order is the same as in Figure 2.

Table S7. K_{RA} mean for each compound and for each data set in the meta-analysis. The compound order is the same as in Figure 3.

Table S8. Dimension-less Henry's Law constant values and their sources used for the meta-analysis of K_{RG}H'.

Table S9. PFAS concentrations in rain (pg L^{-1}) and aerosols (pg m^{-3}) samples from Livingston and Deception Island. *= Data from Casas et al. 2020.i Date = start of the rain event or start of aerosol sampling. f Date = end of the rain event or end of aerosol sampling.

Table S10. OPE concentrations in rain (pg L^{-1}) and aerosols (pg m^{-3}) samples from Livingston Island. i Date = start of the rain event or start of aerosol sampling. f Date = end of the rain event or end of aerosol sampling.

Table S11. PAH concentrations in rain (pg L^{-1}) and aerosol (pg m^{-3}) samples from Livingston Island. i Date = start of the rain event or start of aerosol sampling. f Date = end of the rain event or end of aerosol sampling.

Table S12. Pearson's correlations between log K_{RA}, log K_{RG}, log K_{RP} with log K_{aw}, log K_{oa}, log K_{ow}.

References

Annex S1. Analytical Procedures

PFAS where analysed using established methodology with minor modifications[1]. At the research station's laboratory, samples were filtered through precombusted glass fiber filters (47 mm diameter, Whatman 0.7 µm mesh size) and then spiked with 50 pg of nine recovery standards (Table S1). The analytes were extracted using an established solid phase extraction (SPE) method with OASIS WAX cartridges (6 cm^3, 150 mg; Waters) with minor modifications. The cartridges were previously conditioned with 4 mL of methanol, 4 mL of methanol containing 0.1% ammonia and 4 mL of precleaned HPLC-grade water. After loading 2 L of rain through the cartridges, these were washed with 4 mL of HPLC-grade water, dried under vacuum, and stored at -20◦C in sealed bags. After the sampling campaign, the samples were extracted in a ultraclean laboratory at the Institute of Environemntal Assessment and Water Research (IDAEA-CSIC) . Briefly, OASIS WAX cartridges were pH conditioned with 4 mL of ammonium acetate buffer (25 mM pH 4) and centrifuged to remove the remaining water and then eluted with methanol, followed by methanol containing 0.1% ammonia. QFFs were lyophilized to eliminate water and then spiked with recovery standards (Table S1). After addition of 15 mL of methanol, the vials were vortexed, sonicated for 20 min, and centrifuged for 5 min at 4000 rpm. The supernatant was collected, and the extraction was repeated twice with 5 mL of methanol. The three extracts were combined and then concentrated to 1 mL under a gentle stream of N_2. The extract was cleaned with 1mg ENVI-carb cartridge (1 cm^3, 100 mg, Supelco) , precleaned with 3 mL of methanol. The cartridge was then eluted with 3 mL of methanol. The extract was again reduced under a gentle stream of N_2 to 1 mL and then diluted in 50 mL of precleaned HPLC-grade water and extracted using SPE-OASIS WAX as rain samples. The final eluents (methanol containing 0.1% ammonia) were concentrated under N_2 and reconstituted with 50:50 methanol/HPLC-grade water before the PFAS analysis by Ulta Performance Liquid Chromatography tandem triple quadrupole mass spectrometry (UPLC-MS/MS).

Before the injection, the vials were spiked with a labeled mixture containing six ^{13}C labelled perfluoroalkyl carboxylates (PFCA), and two ^{13}C labelled perfluoroalkyl sulfonates (PFSA) used as injection standards (Table S1). Extracts were analyzed using a Waters UPLC-MS/MS system equipped with a XEVO TQS (Waters, Milford,MA) based on an established method with minor modifications[1,2]. A PFAS isolator column (Isolator column Waters ACQUITY UPLC) was installed between the pump and injector and used to separate background contaminations from the sample to be analyzed. A guard column (Waters Acquity UPLC BEH C18 1.7 µm Vanguard 2,1 x 5 mm) was installed between the inyector and analytical column to remove potential contamination in the mobile phase

and minimize extra column volumes. Each ten microliter extract was loaded into a Waters Acquity UPLC BEH Shield RP18 analytical column (1.7 µm, 2.1 x 100 mm; Waters) maintained at 50 ˚C.

Mobile phase consisted of water and methanol:acetonitrile (80:20) with a constant 2mM of amonium acetate buffer at flow rate of 0.3 mL min^{-1}. Analytes were ionized with an electrospray ionization (ESI) source operating in negative ion mode. Multiple-reaction-monitoring (MRM) mode was used for data adquisition. Each sample was injected in duplicate. To eliminate any potential carryover, acetonitrile was injected in duplicate and passed through the system after every sample or calibration standard.

OPEs and PAHs where extracted using SPE with Oasis hydrophilic-lipophilic balance (HLB) cartridges (6 cm^3, 200 mg; Waters)[3,4]. At the research station's laboratory, rain samples were filtered through precombusted glass fiber filters (47 mm, GF/F Whatman) and then spiked with the recovery standards (Table S1). The HLB cartridges were conditioned with 6 mL of 2-propanol and 12 ml of HPLC-grade water. After loading 2 L of rain through the cartridges, these were washed with 6 mL of chromatographic-grade water at 5% of methanol, dried under vacuum, and stored at -20◦C in sealed bags. After the sampling campaign, the samples were extracted in a ultraclean laboratory at the IDAEA-CSIC.

OPEs and PAHs were eluted with 12 mL methyl tert butyl ether: methanol (9:1; v/v). The residual water was removed by adding 3 g of baked sodium sulfate. The final eluents were concentrated under N_2 and reconstituted in 200 µL of toluene. For OPEs and PAHs analysis in QFF samples followed an established methodology with minor modifications[5]. QFFs were lyophilized overnight, weighted and then spiked with recovery standards (Table S1) and Soxhlet extracted with a mixture of dichloromethane: methanol (2:1, v/v) for 24 h. The extracts were rota-evaporated and fractionated on a deactivated alumina column with a top layer sodium sulphate. Each column was eluted first with 25 mL of hexane (not containing target compounds), a second fraction with 40 mL of dichloromethane: hexane (1:3; v/v) and a third fraction with 20 mL of dichloromethane: acetone (7:3; v/v). The third fraction was further cleaned-up using small deactivated alumina. PAHs were eluted in the second fraction and OPEs were eluted in the second and third fraction. These fractions were concentrated to 0.5 mL with the rotatory evaporator, transferred to a amber vials with the corresponding isooctane washing and concentrated to 150 µL under gentle N_2 flow. PAHs analysis were conducted by gas chromatography (GC) coupled with a mass spectrometer (GC-MS) and OPEs by gas chromatography coupled with a triple quadrupole mass spectrometer (GC-MS/MS).

Internal standards were spiked before all instrumental analysis for quantification and to evaluate instrument performance (Table S1).

The chromatographic separation for OPEs was carried out using an Agilent HP-5MS column (30 m, 0.25 mm internal diameter, 0.25 µm film thickness) by an Agilent 7890 GC. Methane was used as ionization gas and helium was used as a carrier gas at a constant flow mode at 20ml min^{-1}. Two µL of sample were injected in split less mode and the injection port temperature was 280 °C. The column temperature ramp for an effective separation of analytes were as follows; 90 °C for 1 min, increased at 15 °C min^{-1} to 200°C and held for 6 min, then at 5 °C min^{-1} to 250 °C, and held for 6 min and then at 10°C min^{-1} to 315°C, and held for 10 min. The detection was carried out with an Agilent 7000B triple quadrupole mass spectrometer using electro impact ionization (EI) mode in positive conditions. The EI source, transfer line and quadrupole temperature were 230°C, 280 °C and 300 °C, respectively. Acquisition was performed in MRM.

The chromatographic separation for PAHs was carried out using an Agilent DB-5MS column (30 m, 0.25 mm internal diameter, 0.25 µm film thickness) by Agilent 7890 GC. Two µL of sample were injected in split less mode. The initial GC oven temperature was set at 90°C. It was risen up to 175°C at a rate of 6 °C min^{-1} and held for 4 min. Then the temperature increasing rate slowed to 3 °C min^{-1} until 235 °C. After, the heating rate was switched to 8 °C min^{-1} and held for 8 min, until 300 °C. At last, with the same rate, the temperature reached 315 °C during 4 min. The detection was carried out with an Agilent 5975C mass spectrometer using EI mode in positive conditions. Acquisition was performed in selected ion monitoring (SIM).

Annex S2. Quality Assurance/Quality Control

All recipients, tubes and connections used from the sampling to the chemical analysis of OPEs and PAHs were made of stainless steel, glass or PTFE. Nevertheless, for PFAS analysis all recipients were made of stainless steel or PP. These were pre-cleaned with methanol and acetone prior use in order to avoid contamination. All filters were pre-combusted at 450°C over 4h.

Procedural blanks, consisting of QFFs and SPE cartridges, were processed analogously to samples. In addition, field blanks consisted of QFFs and cartridges that were transported to sampling sites, shipped back to the laboratory with samples, processed in the same manner as samples albeit without the pass of rain water or air. Recovery of surrogate standards spiked before the extraction for the different types of samples and for each compound family are presented in Table S2. The limits of detection (LODs) were defined as the mean concentration of field blanks plus three times the standard deviation

of the blank value. For the analytes not detected in blanks, LOD were derived from the lowest standard in calibration curve. Limits of detection are presented in Table S3.

Annex S3. Uncertainty error propagation estimation for K_{RG}/K_{SA}. Where se is the standard error.

$$Uncertainty\ error\ propagation\ for\ each\ compound = \frac{se\ \frac{K_{RG}}{K_{SA}}}{|mean\frac{K_{RG}}{K_{SA}}|}$$

$$= \sqrt{\left(\frac{se\ K_{RG}}{mean\ K_{RG}}\right)^2 + \left(\frac{se\ \ K_{SA}}{mean\ K_{SA}}\right)^2}$$

Figure S1. Sampling location for the rain and aerosol samples analysed in this study.

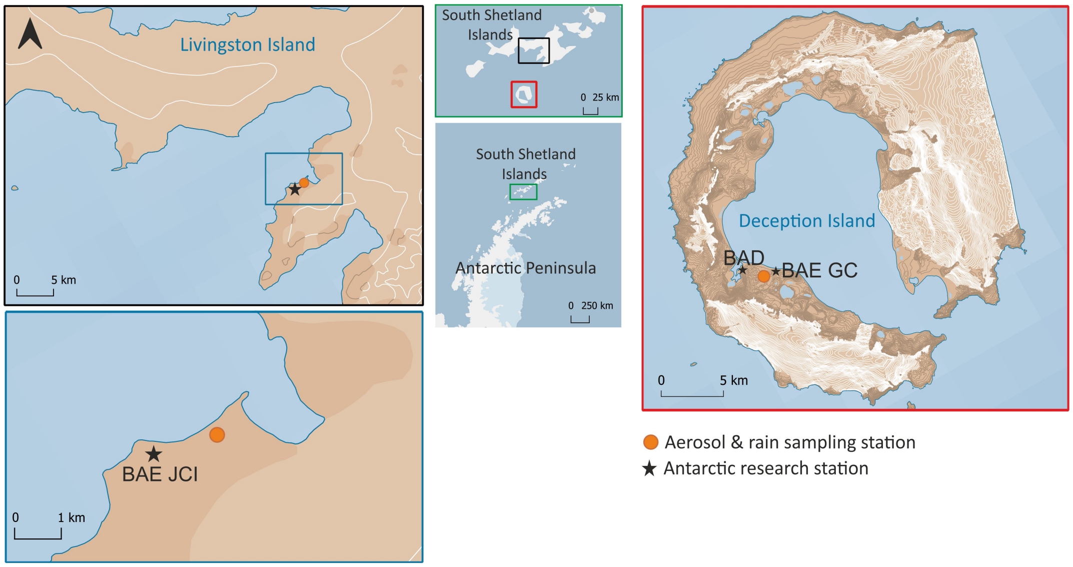

Figure S2. PFAS concentrations in aerosol (pg m^{-3}, upper panel) and rain (pg L^{-1}, lower panel) samples from Deception and Livingston Island.

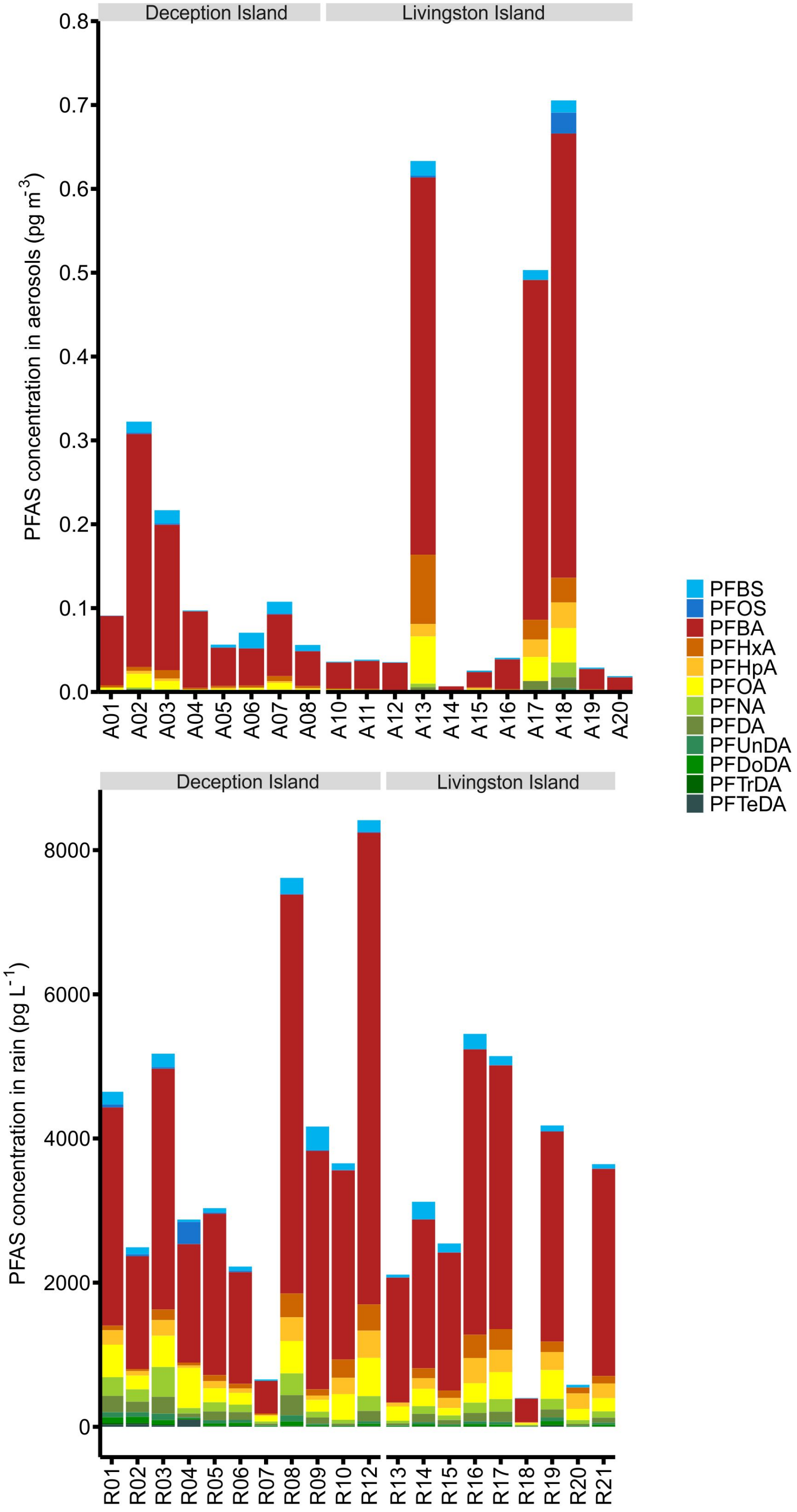

Figure S3. OPE concentrations in aerosol (pg m^{-3}, upper panel) and rain (pg L^{-1}, lower panel) samples from Livingston Island.

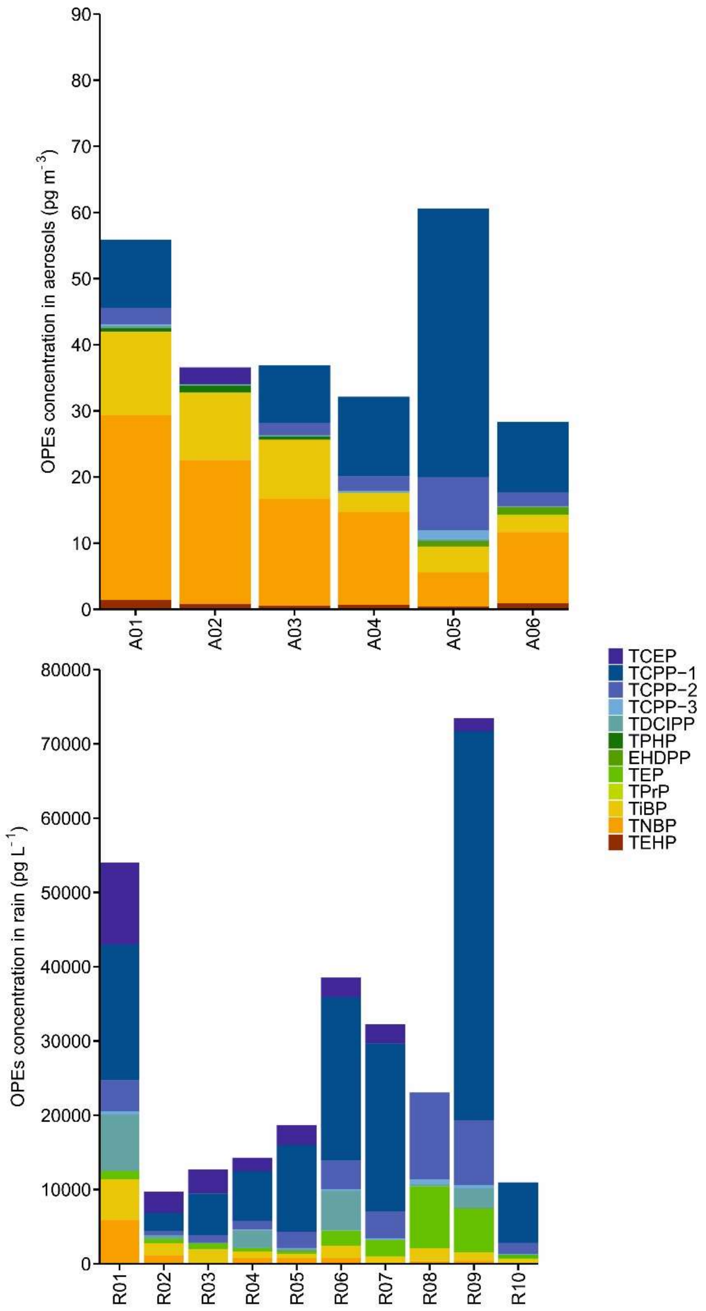

Figure S4. PAH concentrations in aerosol (pg m^{-3}, upper panel) and rain (pg L^{-1}, lower panel) samples at Livingston island.

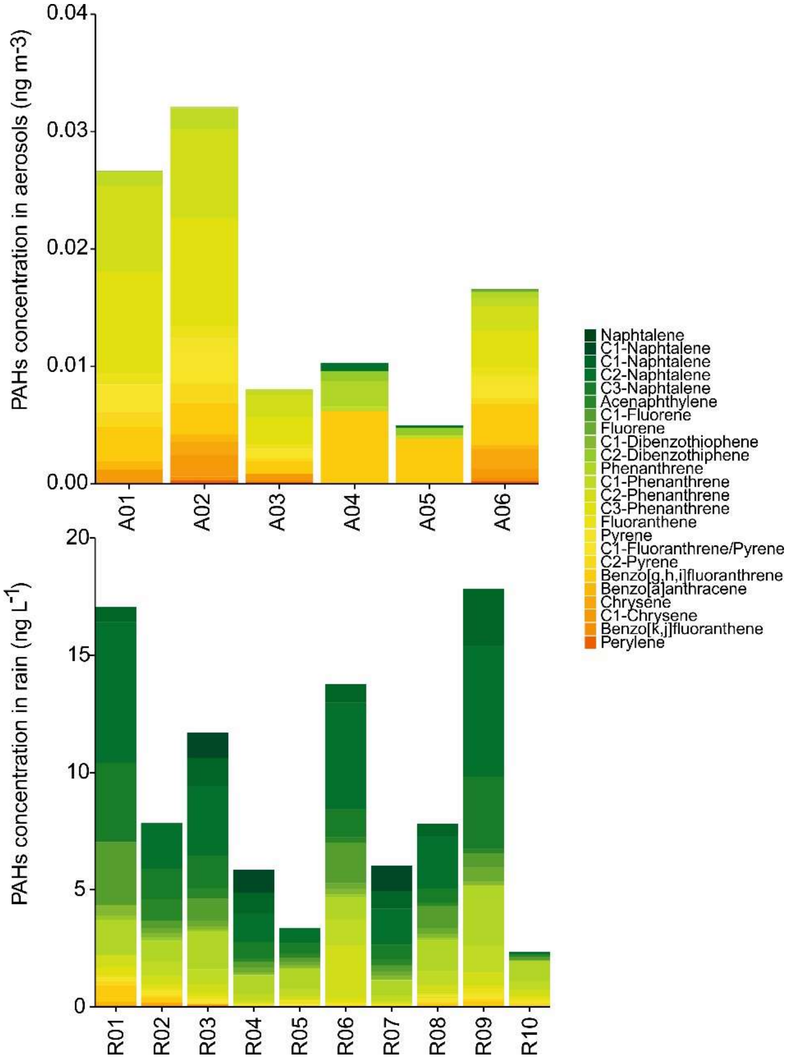

Figure S5. Meta-analysis of rain-air particulate partition constants (K_{RP}) for various families of organic pollutants differentiating the type of aerosol (Continental, Coastal, Open ocean, Urban). The results shown are the mean and the standard deviation of log K_{RP}.

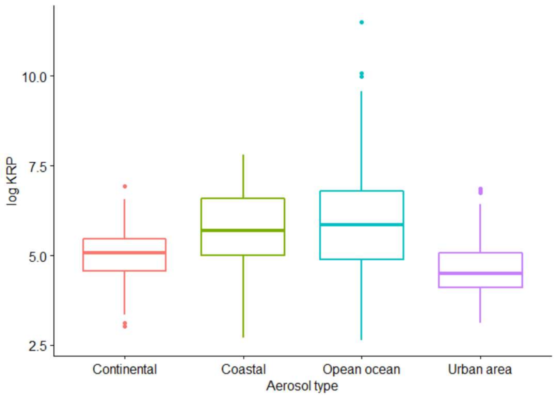

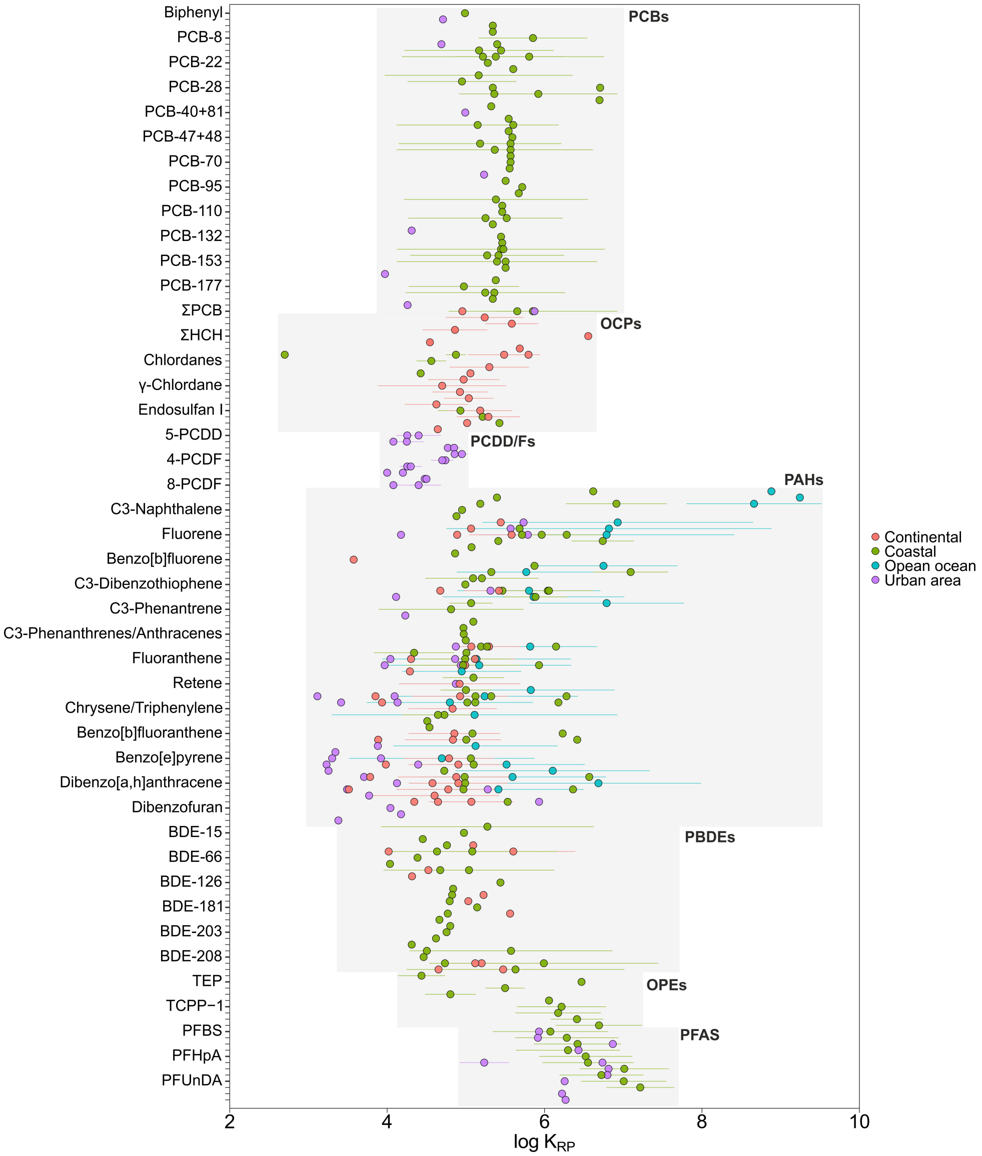

Figure S6. Meta-analysis of rain-air, gas phase adsorbed ($K_{RG,\ adsorbed}$) for various families of organic pollutants. $K_{RG,\ adsorbed}$ as given by Equation [6], $K_{RG} = K_{RG,dissolved} + K_{RG,adsorbed}$, where $K_{RG,dissolved}$ is 1/H'. The results shown are the mean and the standard deviation of log $K_{RG,adsorbed}$.

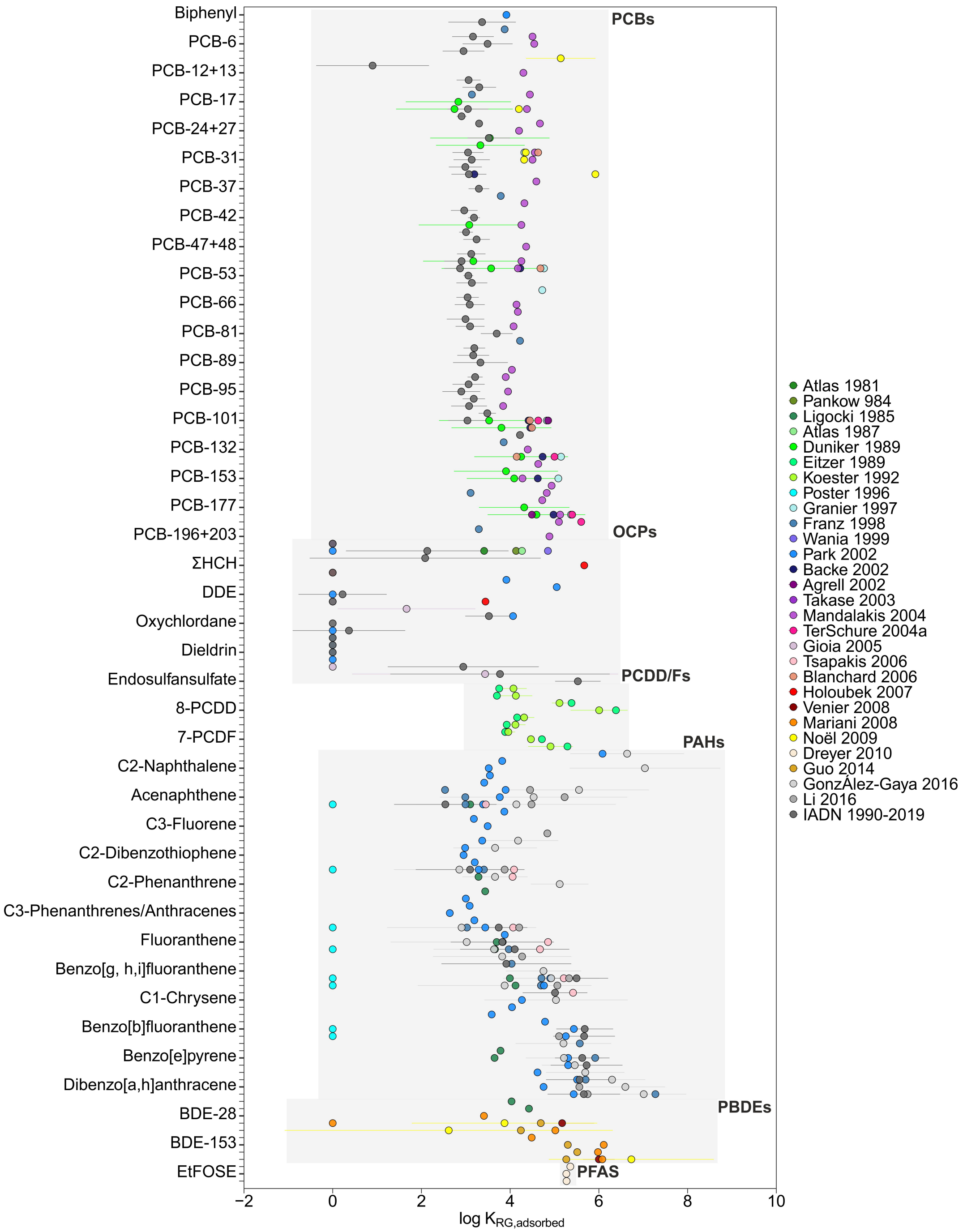

Figure S7. Pearson's correlations between log K_{RA} versus log K_{aw}, log K_{oa}, log K_{ow}.

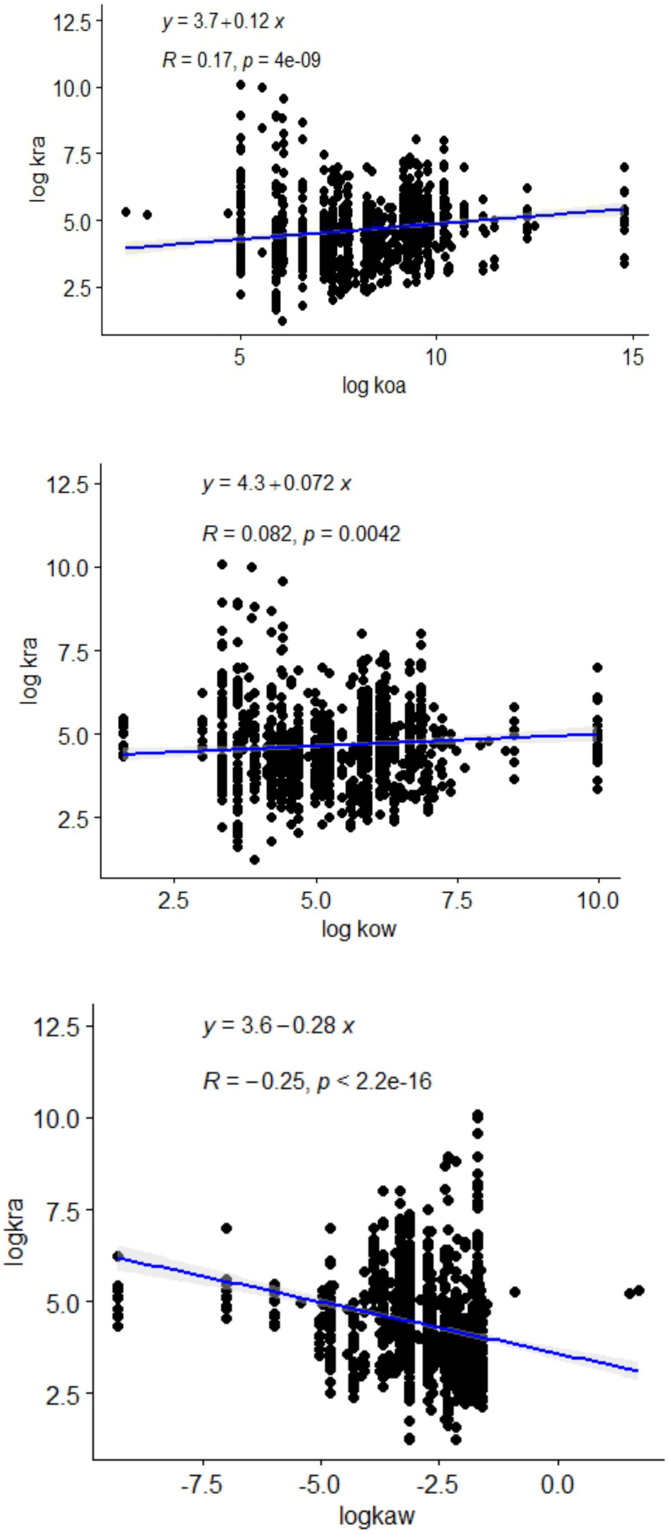

Figure S8. Pearson's correlations between log K_{RG} versus log K_{aw}, log K_{oa}, log K_{ow}.

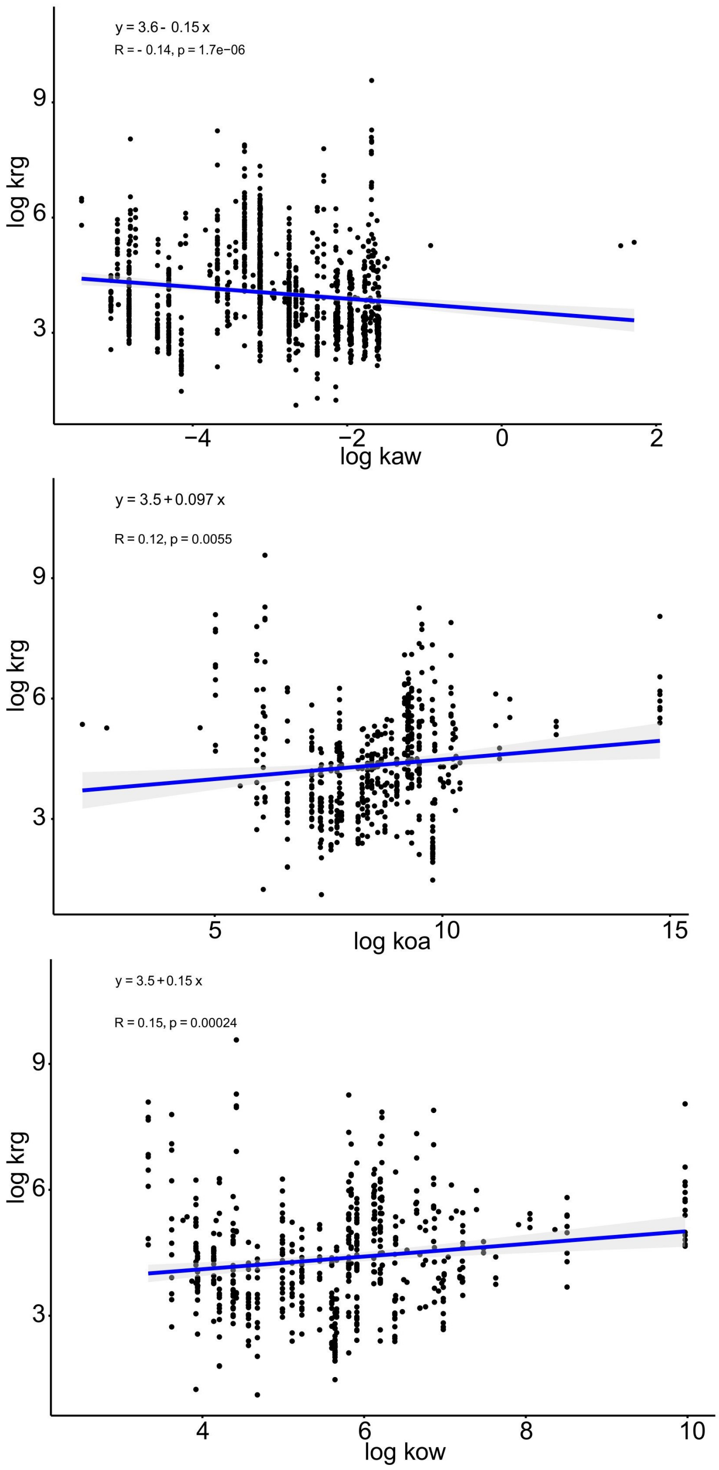

Figure S9. Pearson's correlations between log K_{RP} versus log K_{aw}, log K_{oa}, log K_{ow}.

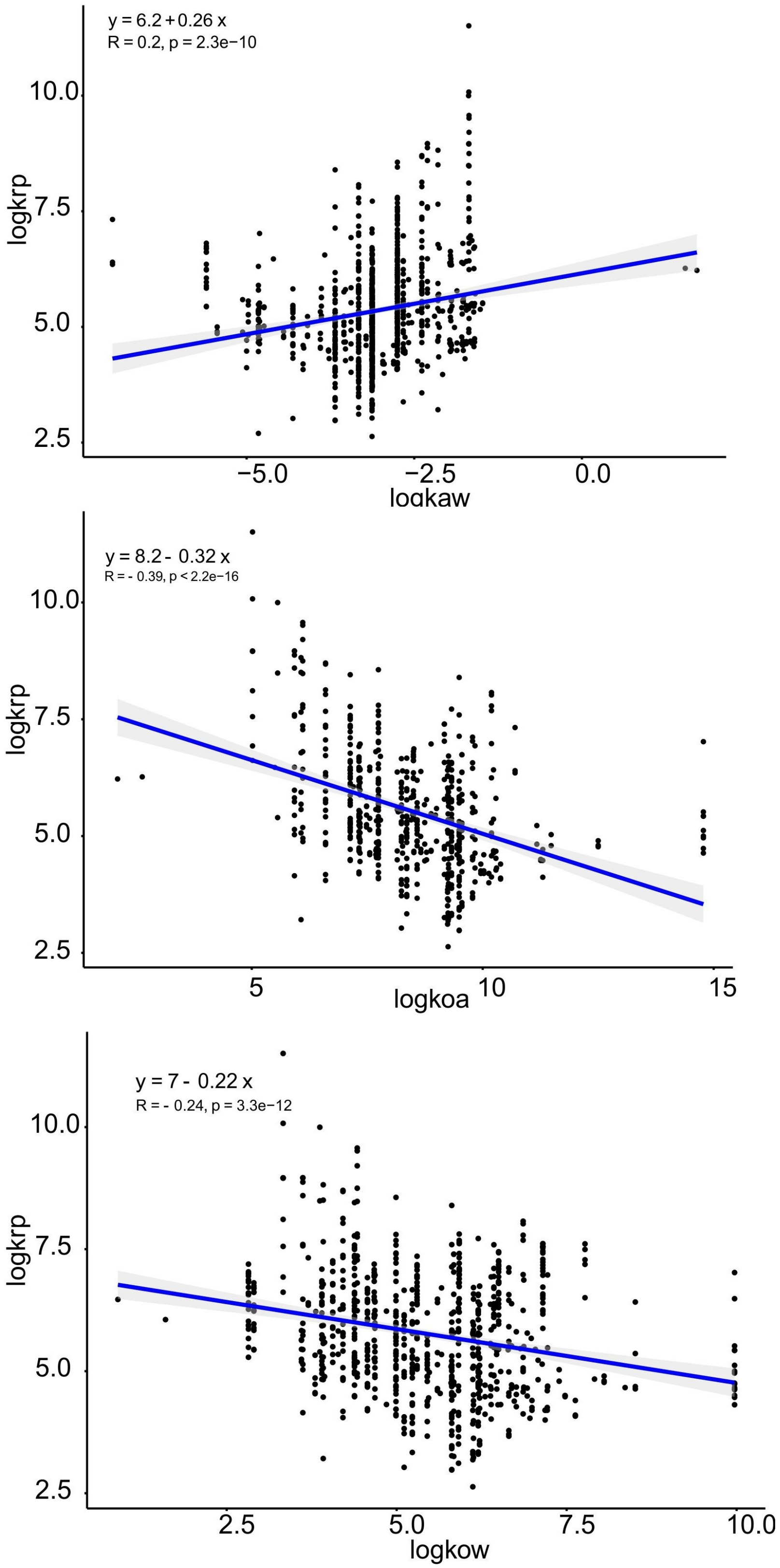

Table S1. Target, recovery and internal standards for PFAS (LC-MS/MS), OPEs (GC-MS/MS) and PAHs (GC-MS) analyzed in the present study.

Compound	Acronym	Precursor Ion	Product Ion
**Perfluoroalkyl sulfonic acids**	**PFSA**		
Perfluorobutane sulfonic acid	PFBS	299	80
Perfluorohexane sulfonic acid	PFHxS	399	80
Perfluorooctane sulfonic acid	PFOS	499	80
Perfluorodecane sulfonic acid	PFDS	599	80
Perfluorododecane sulfonic acid	PFDoDS*	699	80
Perfluoroethylcyclohexane sufonate	PFECHS*	461	381
**Perfluoroalkyl carboxylic acids**	**PFCA**		
Perfluorobutanoic acid	PFBA	213	169
Perfluoropentanoic acid	PFPeA*	263	219
Perfluorohexanoic acid	PFHxA	313	269
Perfluoroheptanoic acid	PFHpA	363	319
Perfluorooctanoic acid	PFOA	413	369
Perfluorononanoic acid	PFNA	463	419
Perfluorodecanoic acid	PFDA	513	469
Perfluoroundecanoic acid	PFUnDA	563	519
Perfluorododecanoic acid	PFDoDA	613	569
Perfluorotridecanoic acid	PFTrDA	663	619
Perfluorotetradecanoic acid	PFTeDA	713	669
Perfluorohexadecanoic acid	PFHxDA*	813	769
Perfluorooctadecanoic acid	PFODA*	913	869
**Recovery standard**			
Perfluoro-n-[$^{13}C_4$]butanoic acid	PFBA $^{13}C_4$	217	172
Perfluoro-n-[1,2-$^{13}C_2$]hexanoic acid	PFHxA $^{13}C_2$	315	270
Perfluoro-n-[1,2,3,4-$^{13}C_4$]octanoic acid	PFOA $^{13}C_4$	417	372
Perfluoro-n-[1,2,3,4,5-$^{13}C_5$]nonanoic acid	PFNA $^{13}C_5$	468	423
Perfluoro-n-[1,2-$^{13}C_2$]decanoic acid	PFDA $^{13}C_2$	515	470
Perfluoro-n-[1,2-$^{13}C_2$]undecanoic acid	PFUnDA $^{13}C_2$	570	525
Perfluoro-n-[1,2-$^{13}C_2$]dodecanoic acid	PFDoDA $^{13}C_2$	615	570
Sodium perfluoro-1-hexane[$^{18}O_2$]sulfonate	PFHxS $^{18}O_2$	403	84
Sodium perfluoro-1-[1,2,3,4-$^{13}C_4$]octanesulfonate	PFOS $^{13}C_4$	503	80
**Internal standard**			
Perfluoro-n-[2,3,4-$^{13}C_3$]butanoic acid	PFBA $^{13}C_3$	216	172
Perfluoro-n-[$^{13}C_5$]pentanoic acid	PFPeA $^{13}C_5$	268	223
Sodium perfluoro-1-[$^{13}C_8$]-octanesulfonate	PFOS $^{13}C_8$	507	80
Perfluoro-n-[$^{13}C_8$]octanoic acid	PFOA $^{13}C_8$	421	376
Sodium perfluoro-1-[1,2,3-$^{13}C_3$]-hexanesulfonate	PFHxS $^{13}C_3$	402	99
Perfluoro-n-[1,2,3,4,5,6,7-$^{13}C_7$]undecanoic acid	PFUnDA $^{13}C_7$	570	525

Those compounds with * were not detected.

Compound	Acronym	Transition	Precursor ion	Product ion
Tris (2-chloroethyl) phosphate	TCEP	249->125	249	99
Tris(2-chloroisoprpyl) phosphate	TCIPP*	125->99	201	125
Tris(1,3-dichloro-2-propyl) phosphate	TDCIPP	208.9->99	380.9	159
Tris(2,3-dibromopropyl) phosphate	TDBPP*	216.9->137	216.9	99
Triphenylphosphine oxide	TPPO*	277.2->199	199	152.1
Triphenyl phosphate	TPHP	326->215	326	169
2-ethylhexyldiphenyl phosphate	EHDPP	251->77	250	170
Tr-p-totyl phosphate / Tris(p-cresyl) phosphate	TpCP*	368.1->165	368.1	107
Tri-m-totyl phosphate / Tris(m-cresyl) phosphate	TmCP*	368.1->165	368.1	91
Tris(3,5-dimethylphenyl) phosphate	TDMPP*	410->193.1	410.1	395
Tris(2-isopropylphenyl) phosphate	TPPP*	452.2->118	452.2	251
Tri-iso-buthyl phosphate	TiBP	155.1->99.1	211.2	99.1
Tributyl phosphate/ tri-n-butylphospate	TNBP	155.1->99.1	211.2	99.1
Tris(2-butoxyethyl)phosphate	TBEP*	299.2->199.1	199	101.1
Tris(2-ethylhexyl) phosphate	TEHP	99->80.9	112.9	94.8
Triethylphosphate	TEP	155->99	127	99
Tripropyl phosphate	TPrP	141.1->99	183.2	99
Tributylphosphine oxide	TBPO*	92->77	189.1	78
Tri-o-totyl phosphate / Tris(o-cresyl) phosphate	ToCP*	368.1->165	368.1	179.1
Dioctyl phenylphosphonate	DOPP*	159->141	271.1	159.1
Tris(4-tert-butylphenyl) phosphate	TTBPP*	479.2->211.2	479.2	57.1
**Recovery standard**				
Tri-n-buthylphophate-d27	D27-TNBP	167.4 -> 103	231.4	103
Triphenyl phosphate-d15	D15-TPhP	341.1->240.1	241.1	223

**Internal standard**			494.3	479.3
Tris(2-chloroethyl) phosphate -d12	D12-TCEP	261.1->148	261.1	213
Tris(1,3-dichloro-2-propyl) phosphate -d15	D15-TDCIPP	196.6->79.1	393.9	196.9
			394.3	164.1

Those compounds with * were not detected.

Compound	Main ion	Confirmation ion
Naphtalene	128	127
C1-Naphtalene	142	141
C1 -Naphtalene	142	-
C2- Naphtalene	156	-
Acenaphthylene	152	151
Acenaphtene	153	154
C3 -Naphtalene	170	155
Fluorene	166	165
C1- Fluorene	180	155
Dibenzothiophene	184	-
Phenanthrene	178	176
Anthracene	178	176
C1 -Dibenzothiophene	198	183
C1- Phenanthrene	192	-
C2- Dibenzothiophene	212	197
C2 -Phenanthrene	206	191
Fluoranthrene	202	200
Pyrene	202	200
C3- Phenanthrene	220	205
C3- Phenanthrene	220	205
P-therpenyl d14	244	122
C1- Fluoranthrene	216	215
C1- Pyrene	216	215
Rethene	234	-
C4- Phenanthrene	234	219
C4- Phenanthrene	234	219
Benzonaphtothiophene	234	117
C2- Pyrene	230	215
Benzo[ghi]fluoranthrene	226	113
Benzo[a]anthracene	228	226
Crysene	228	226
Triphenylene	228	226
C1- Chrysene	242	119
Benzo[b]fluoranthene	252	250
Benzo[k]fluoranthene	252	250
Benzo[j]fluoranthene	252	-
Benzo[e]pyrene	252	-

Benzo[a]pyrene	252	250
Perylene	252	250
Indeno[1,2,3-cd]pyrene	276	277
Dibenzo[a,h]anthracene	278	276
Benzo[ghi]perylene	276	277
Coronene	300	150
**Recovery standard**		
Naphtalene - d8	136	128
Acenaphtene - d10	164	
Phenanthrene - d10	188	
Crysene - d12	240	
Perylene - d12	264	
Anthracene - d10	188	
Benzo[a]fluoranthene - d12	264	
**Internal standard**		
Crysene - d12	164	
Perylene - d12	188	

Table S2. PFAS, OPEs and PAHs sample recoveries of recovery standards (%) for rain and aerosols samples.

Samples	PFBA-$^{13}C_4$	PFOA-$^{13}C_4$	PFHxA-$^{13}C_2$	PFHxS-$^{18}O_2$	PFNA-$^{13}C_5$	PFOS-$^{13}C_4$	PFDA-$^{13}C_2$	PFUnDA-$^{13}C_2$	PFDoDA-$^{13}C_2$
Rain (%)	54	45	37	71	41	53	26	17	13
Aerosols (%)	58	68	68	79	65	80	69	55	38

Samples	D27-TNBP	D15-TPhP
Rain (%)	107	50
Aerosols (%)	104	41

	D10-Acenaphtene	D12-Perylene	D12-Chrysene	D10-Phenanthrene	D8-Naphthalene
Rain (%)	21	31	52	34	15
Aerosols (%)	18	80	78	38	8

Table S3. Limits of detection for PFAS, OPEs for rain and aerosols samples from Deception and Livingston s. The limits of detection (LODs) were defined as the mean concentration of field blanks plus three times the standard deviation of the blank response. For the analytes not detected in blanks, LOD were derived from the lowest standard in calibration curve.

LODs (pg)	Samples	PFBA	PFPeA	PFBS	PFHxA	PFHpA	PFHxS	PFOA	PFECHS	PFNA	PFOS
Aerosols	Deception	0.4	0.2	1.3	0.2	2.0	0.2	1.8	7.3	0.3	0.1
Aerosols	Livingston	0.4	0.2	0.9	1.6	2.7	0.2	1.9	7.3	0.4	0.1
Rain	Deception	7.7	0.2	0.1	0.2	0.5	0.2	1.3	7.3	0.1	1.6
Rain	Livingston	4.8	0.2	0.1	0.2	0.5	0.2	0.4	7.3	0.1	0.1

LODs (pg)	Samples	PFDA	PFUnDA	PFDS	PFDoDA	PFTrDA	PFDoDeS	PFTeDA	PFHxDA	PFODA
Aerosols	Deception	0.5	0.1	0.1	0.2	0.1	0.1	0.1	0.2	1.2
Aerosols	Livingston	0.6	0.1	0.1	0.2	0.1	0.1	0.1	0.2	1.2
Rain	Deception	0.1	0.1	0.1	0.2	0.1	0.1	0.1	0.2	1.2
Rain	Livingston	0.1	0.1	0.1	0.2	0.1	0.1	0.1	0.2	1.2

LODs (ng)	TEP	TPrP	TiBP	TNBP	TCEP	TCPP-2	TCPP-3	TCPP-1	TDClPP	TPHP	EHDPP	TEHP
Rain	0.28	0.06	0.40	1.5	3.3	0.53	0.06	2.9	0.4	1.7	0.07	0.07
Aerosols	0.10	0.002	3.4	3.3	4.3	3.1	0.17	14	0.5	1.7	1.4	0.001

Table S4. Information of the data used in the meta-analysis of rain-air partition constants.

	Publication	POP family	Kr	Rain Sampling points	Air sampling points	#Rain samples	#Air samples	Rain phase	Air phase	Calculation	Location	
1	Bidelman & Christensen 1979	PCBs, OCPs	Kra					Dissolved&particulate	Gas & particulate	Given	Columbia, South Carolina	Urban area
2	Atlas & Giam 1981	OCPs	Kra	2	2			Dissolved&particulate	Gas & particulate	Given	Collage Satation, Texas	Continental
3	Pankow et al. 1984	OCPs	Kra					Dissolved&particulate	Gas & particulate	Given	Portland, Oregon Graduate Center (OGC)	Urban area
4	Ligocki et al. 1985	PAHs	Kra, Krp, Krg	1	1	7	7	Dissolved&particulate	Gas & particulate	Given	Southeast Portland	Urban area
5	McVeety & Hites 1987	PAHs	Kra					Dissolved+particulate	Gas & particulate	Given	Isle Royal, Lake Superior, Michigan	Continental
6	Atlas & Giam 1988	PCBs, OCPs	Kra					Dissolved+particulate	Gas & particulate	Given	College station, Texas	Continental
7	Duinker & Bouchertall 1989	PCBs	Kra, Krp, Krg	1	1	3	3	Dissolved+particulate	Gas & particulate	Calculated	Kiel,Western Baltic	Costal
8	EitzerT & Hites 1989	PCDD/Fs	Kra, Krp, Krg	4	4			Dissolved&particulate	Gas & particulate	Given	Bloomingstin, Indianapolis, USA	Urban area
9	Koester & Hites 1992	PCDD/Fs	Krp, Krg	2	2			Dissolved+particulate	Gas & particulate	Given	Bloomingstin, Indianapolis, USA	Urban area
10	Dickhut & Gustafson 1995	PAHs	Krg					Dissolved&particulate	Gas	Given	Chesapeak Bay watershed, rural location	Costal
11	Poster & Baker 1996	PAHs	Kra, Krp, Krg	1	1	5		Dissolved	Gas & particulate	Given	Chesapeake Bay, Mid-Atlantic region,USA	Costal
12	Granier & Chevreuil 1997	PCBs	Kra					Dissolved+particulate	Gas+particulate	Given	Paris	Urban area
13	Franz &Eisenreich 1998	PCBs, PAHs	Kra, Krp, Krg	1	1		9 (gas), 3 (particulate)	Dissolved+particulate	Gas & particulate	Given	Minneapolis/St. Paul,Minnesota	Urban area
14	Wania & Haugen 1999	OCPs	Krg,kra					Dissolved+particulate	Gas & particulate	Given	Lista station; Norway	Costal
15	Park et al. 2002	PCBs,OCPs, PAHs	Kra, Krp, Krg	1	1		40	Dissolved+particulate	Gas & particulate	Calculated	Corpus Christi Bay, Texas	Costal
16	VanRy et al. 2002	PCBs	Kra, Krp, Krg	7	7	193		Dissolved	Gas & particulate	Calculated	New Jersey	Costal
17	Offenberg & Baker 2002	PCBs, PAHs	Kra	4	4	14	14	Dissolved+particulate	Gas+particulate	Given	Lake Michigan, Chicago	Continental
18	Backe et al. 2002	PCBs	Kra	11	11	206	206	Dissolved+particulate	Gas	Given	Scania,Southern Sweden	Continental
19	Agrell et al. 2002	PCBs	Krg,Kra	16	16	266	299	Dissolved+particulate	Gas	Given	Baltic sea	Costal
20	Takase et al. 2003	OCPs	Krg,kra	3	3	7	7	Dissolved	Gas	Calculated	Niigata, Japan	Urban area
21	Mandalakis & Stephanou 2004	PCBs	Kra, Krp, Krg	2	2	14	14	Dissolved+particulate	Gas & particulate	Given	Eastern Mediterranean	Costal
22	Sahu et al. 2004	PAHs	Kra	1	1			Dissolved	Gas+particulate	Given	Mumbai, India	Urban area
23	Ter Schure et al. 2004a	PCBs, PBDEs	Kra	1	1			Dissolved+particulate	Gas+particulate	Given	Gotska Sandön, Baltic Proper	Urban area
24	Ter Schure et al. 2004b	PBDEs	Kra					Dissolved+particulate	Gas+particulate	Given	Southern Sweden	Urban area
25	Gioia et al. 2005	OCPs	Kra, Krp, Krg	6	6			Dissolved+particulate	Gas & particulate	Calculated	US Mid-Atlantic region	Costal
26	Tsapakis et al. 2006	PAHs	Krg	1	1	7	7	Dissolved+particulate	Gas	Calculated	Crete, Eastern Mediterranean Basin	Costal
27	Blanchard et al. 2006	PAHs	Kra	5	5	60	60	Dissolved+particulate	Gas+particulate	Given	Northern France	Continental
28	Barton et al. 2007	PFAS (PFOA)	Krp	4	4			Dissolved	Particulate	Calculated	Parkerburg, West Virginia	Urban area
29	Holoubek et al. 2007	PCBs, OCPs	Kra, Krp, Krg	1	1			Dissolved	Gas & particulate	Calculated	Kosetice observatory, Czech Republic	Continental
30	Venier & Hites 2008	PBDEs	Kra, Krp, Krg	6	6			Dissolved+particulate	Gas & particulate	Calculated	Great Lake	Continental
31	Mariani et al. 2008	PBDEs	Kra, Krp, Krg	10	10	5	5	Dissolved	Gas & particulate	Calculated	Lake Maggiore, Northern Italy	Continental
32	He & Balasubramanian 2009	PAHs	Kra	1	1	31	37	Dissolved+particulate	Gas+particulate	Calculated	National University of Singapore (NUS)	Urban area
33	Noël et al. 2009	PCBs, PBDEs	Kra, Krp, Krg	2	2		365	Dissolved+particulate	Gas+particulate	Given	Vancouver Island,British Columbia, Canada	Costal
34	Zhang et al. 2009	PBDEs	Kra	2	2	23	34	Dissolved+particulate	Gas+particulate	Given	Pearl River Delta, China	Continental
35	Birgül et al. 2010	PAHs	Krp, Krg	1	1	19	36	Dissolved&particulate	Gas+particulate	Given	Butal, Urban Area of Turkey	Urban area
36	Dreyer et al. 2010	PFAS	Krp,Krg,Kra	2	2	7	7	Dissolved	Gas & particulate	Calculated	Hamburg, Germany	Urban area
37	Günindi et al. 2011	PCBs	Kra, Krp, Krg	1	1	25	25	Dissolved+particulate	Gas & particulate	Given	Butal, Urban Area of Turkey	Urban area
38	Liu et al.2013	PAHs	Krp					Dissolved+particulate	Particulate	Given	Southeastern Hong Kong Island, South China	Costal
39	Guo et al. 2014	PBDEs	Krp,Krg,Kra	3	3	19	40	Dissolved+particulate	Gas & particulate	Calculated	Pearl River Delta, China	Costal
40	Shahpoury et al. 2015	PAHs	Kra	1	1	231	162	Dissolved+particulate	Gas+particulate	Given	Kosetice observatory, Czech Republic	Continental
41	Zhang et al. 2015	PAHs	Kra	3	3	30	30	Dissolved+particulate	Gas+particulate	Calculated	Athabasca oil sands region, Alberta, Canada	Urban impacted
42	González-Gaya et al. 2016	PAHs	Krp,Krg,Kra	12	12	12	12	Dissolved	Gas & particulate	Calculated	Malaspina circumnavigation expedition	Open ocean
43	Li et al. 2016	PAHs	Krp,Krg,Kra	1	1	34	39	Dissolved&particulate	Gas & particulate	Calculated	Mt. Heng, China	Continental
44	IADN 1990-2019	PCBs, OCPs, PAHs	Kra, Krp, Krg	6	6	PCBs: 6905<br>OCPs: 9443<br>PAHs: 7702	Filter:<br>OCPs: 8495<br>PAHs: 15540<br>Vapor:<br>PCBs: 51103<br>OCPs: 26004<br>PAHs: 26004	Dissolved+particulate	Gas & particulate	Calculated	Greate Lakes	Continental
45	Zhang et al. 2020	OPEs	Kra	9	9	12	12	Dissolved	Gas & particulate	Calculated	Nanning City, China	Urban area
46	This study	PAHs, PFAS, OPEs	Krp	1	1	PAHs&OPEs: 10<br>PFAS: 20	PAHs&OPEs: 10<br>PFAS: 20	Dissolved	Particulate	Calculated	Livingston and Deception Island,<br>Antarctic Peninsula	Costal

Table S5. K_{RP} mean for each compound and for each data set in the meta-analysis. The compound order is the same as in Figure 1.

Compound family	Compound	Reference	log K_{RP} Mean
PCB	Biphenyl	Park et al. 2002	5.0
PCB	PCB-4+15	Franz & Eisenreich 1998	4.7
PCB	PCB-5-8	Mandalakis & Stephanou 2004	5.3
PCB	PCB-6	Mandalakis & Stephanou 2004	5.3
PCB	PCB-8	Noël et al. 2009	5.9
PCB	PCB-16+39	Franz & Eisenreich 1998	4.7
PCB	PCB-16+39	Mandalakis & Stephanou 2004	5.4
PCB	PCB-17	Duniker & Bouchertall 1989	5.2
PCB	PCB-17	Mandalakis & Stephanou 2004	5.4
PCB	PCB-18	Duniker & Bouchertall 1989	5.2
PCB	PCB-18	Mandalakis & Stephanou 2004	5.4
PCB	PCB-18	Noël et al. 2009	5.8
PCB	PCB-22	Mandalakis & Stephanou 2004	5.3
PCB	PCB-24+27	Mandalakis & Stephanou 2004	5.6
PCB	PCB-26	Duniker & Bouchertall 1989	5.2
PCB	PCB-27	Duniker & Bouchertall 1989	5.0
PCB	PCB-28	Mandalakis & Stephanou 2004	5.3
PCB	PCB-28	Noël et al. 2009	6.7
PCB	PCB-31	Mandalakis & Stephanou 2004	5.4
PCB	PCB-31	Noël et al. 2009	5.9
PCB	PCB-33	Noël et al. 2009	6.7
PCB	PCB-33+20	Mandalakis & Stephanou 2004	5.3
PCB	PCB-40+81	Franz & Eisenreich 1998	5.0
PCB	PCB-41+46	Mandalakis & Stephanou 2004	5.5
PCB	PCB-44	Duniker & Bouchertall 1989	5.1
PCB	PCB-44	Mandalakis & Stephanou 2004	5.6
PCB	PCB-45	Mandalakis & Stephanou 2004	5.5
PCB	PCB-47+48	Mandalakis & Stephanou 2004	5.6
PCB	PCB-49	Duniker & Bouchertall 1989	5.2
PCB	PCB-49	Mandalakis & Stephanou 2004	5.6
PCB	PCB-52	Duniker & Bouchertall 1989	5.4
PCB	PCB-52	Mandalakis & Stephanou 2004	5.6
PCB	PCB-66	Mandalakis & Stephanou 2004	5.6
PCB	PCB-70	Mandalakis & Stephanou 2004	5.6
PCB	PCB-74	Mandalakis & Stephanou 2004	5.6
PCB	PCB-82+127	Franz & Eisenreich 1998	5.2
PCB	PCB-90+101	Mandalakis & Stephanou 2004	5.5
PCB	PCB-95	Mandalakis & Stephanou 2004	5.7
PCB	PCB-99	Mandalakis & Stephanou 2004	5.7
PCB	PCB-101	Duniker & Bouchertall 1989	5.4
PCB	PCB-105	Mandalakis & Stephanou 2004	5.5
PCB	PCB-110	Mandalakis & Stephanou 2004	5.5
PCB	PCB-118	Duniker & Bouchertall 1989	5.2
PCB	PCB-118	Mandalakis & Stephanou 2004	5.5

PCB	PCB-123	Mandalakis & Stephanou 2004	5.3
PCB	PCB-128+169	Franz & Eisenreich 1998	4.3
PCB	PCB-132	Mandalakis & Stephanou 2004	5.4
PCB	PCB-136	Mandalakis & Stephanou 2004	5.5
PCB	PCB-138	Duniker & Bouchertall 1989	5.4
PCB	PCB-138	Mandalakis & Stephanou 2004	5.5
PCB	PCB-149	Duniker & Bouchertall 1989	5.3
PCB	PCB-149	Mandalakis & Stephanou 2004	5.4
PCB	PCB-153	Duniker & Bouchertall 1989	5.4
PCB	PCB-153	Mandalakis & Stephanou 2004	5.5
PCB	PCB-158+160	Mandalakis & Stephanou 2004	5.5
PCB	PCB-170+190	Franz & Eisenreich 1998	4.0
PCB	PCB-174	Mandalakis & Stephanou 2004	5.4
PCB	PCB-177	Duniker & Bouchertall 1989	5.0
PCB	PCB-180	Duniker & Bouchertall 1989	5.2
PCB	PCB-180	Mandalakis & Stephanou 2004	5.4
PCB	PCB-194	Mandalakis & Stephanou 2004	5.3
PCB	PCB-194+205	Franz & Eisenreich 1998	4.3
PCB	ΣPCB	VanRy et al. 2002	5.7
PCB	ΣPCB	Holoubek et al. 2007	5.0
PCB	ΣPCB	Noël et al. 2009	5.9
PCB	ΣPCB	Günindi et al. 2011	5.9
OCPs	α-HCH	IADN 1990-2019	5.2
OCPs	γ-HCH	IADN 1990-2019	5.6
OCPs	β-HCH	IADN 1990-2019	4.9
OCPs	ΣHCH	Holoubek et al. 2007	6.6
OCPs	HCB	Holoubek et al. 2007	4.5
OCPs	DDD	IADN 1990-2019	5.7
OCPs	DDT	Park et al. 2002	2.7
OCPs	DDT	Gioia et al.2005	4.9
OCPs	DDT	Holoubek et al. 2007	5.8
OCPs	DDT	IADN 1990-2019	5.5
OCPs	Chlordanes	Gioia et al.2005	4.6
OCPs	Heptachlor	IADN 1990-2019	5.3
OCPs	Oxychlordane	Gioia et al.2005	4.4
OCPs	Oxychlordane	IADN 1990-2019	5.1
OCPs	α-Chlordane	IADN 1990-2019	5.0
OCPs	γ-Chlordane	IADN 1990-2019	4.7
OCPs	Trans-nonachlor	IADN 1990-2019	4.9
OCPs	Dieldrin	IADN 1990-2019	5.0
OCPs	Endrin	IADN 1990-2019	4.6
OCPs	EndosulfanI	Gioia et al.2005	4.9
OCPs	EndosulfanI	IADN 1990-2019	5.2
OCPs	EndosulfanII	Gioia et al.2005	5.2
OCPs	EndosulfanII	IADN 1990-2019	5.3
OCPs	Endosulfansulfate	Gioia et al.2005	5.4
OCPs	Endosulfansulfate	IADN 1990-2019	5.0
OCPs	Methoxychlor	IADN 1990-2019	4.6

PCDDFs	5-PCDD	Eitzer & Hites 1989	4.3
PCDDFs	5-PCDD	Koester & Hites 1992	4.4
PCDDFs	6-PCDD	Eitzer & Hites 1989	4.1
PCDDFs	6-PCDD	Koester & Hites 1992	4.3
PCDDFs	7-PCDD	Eitzer & Hites 1989	4.8
PCDDFs	7-PCDD	Koester & Hites 1992	4.9
PCDDFs	8-PCDD	Eitzer & Hites 1989	4.9
PCDDFs	8-PCDD	Koester & Hites 1992	5.0
PCDDFs	4-PCDF	Eitzer & Hites 1989	4.7
PCDDFs	4-PCDF	Koester & Hites 1992	4.7
PCDDFs	5-PCDF	Eitzer & Hites 1989	4.3
PCDDFs	5-PCDF	Koester & Hites 1992	4.3
PCDDFs	6-PCDF	Eitzer & Hites 1989	4.0
PCDDFs	6-PCDF	Koester & Hites 1992	4.2
PCDDFs	7-PCDF	Eitzer & Hites 1989	4.5
PCDDFs	7-PCDF	Koester & Hites 1992	4.5
PCDDFs	8-PCDF	Eitzer & Hites 1989	4.1
PCDDFs	8-PCDF	Koester & Hites 1992	4.4
PAHs	Naphthalene	Park et al. 2002	6.6
PAHs	Naphthalene	Gonzalez-Gaya 2016	8.9
PAHs	C1-Naphthalene	Park et al. 2002	5.4
PAHs	C1-Naphthalene	Gonzalez-Gaya 2016	9.2
PAHs	C2-Naphthalene	Park et al. 2002	5.2
PAHs	C2-Naphthalene	Gonzalez-Gaya 2016	8.7
PAHs	C2-Naphthalene	This study	6.9
PAHs	C3-Naphthalene	Park et al. 2002	5.0
PAHs	C4-Naphthalene	Park et al. 2002	4.9
PAHs	Acenaphthylene	Franz & Eisenreich 1998	5.7
PAHs	Acenaphthylene	Gonzalez-Gaya 2016	6.9
PAHs	Acenaphthylene	Li et al. 2016	5.4
PAHs	Acenaphthene	Franz & Eisenreich 1998	5.6
PAHs	Acenaphthene	Park et al. 2002	5.7
PAHs	Acenaphthene	Gonzalez-Gaya 2016	6.8
PAHs	Acenaphthene	Li et al. 2016	5.1
PAHs	Fluorene	Ligocki et al. 1985	4.2
PAHs	Fluorene	Poster & Baker 1996	6.0
PAHs	Fluorene	Franz & Eisenreich 1998	5.8
PAHs	Fluorene	Park et al. 2002	5.7
PAHs	Fluorene	Gonzalez-Gaya 2016	6.8
PAHs	Fluorene	Li et al. 2016	4.9
PAHs	Fluorene	IADN 1990-2019	5.6
PAHs	Fluorene	This study	6.3
PAHs	C1-Fluorene	Park et al. 2002	5.4
PAHs	C1-Fluorene	This study	6.7
PAHs	C2-Fluorene	Park et al. 2002	5.1
PAHs	C3-Fluorene	Park et al. 2002	4.9
PAHs	Benzo[b]fluorene	Li et al. 2016	3.6
PAHs	Dibenzothiophene	Park et al. 2002	5.9

PAHs	Dibenzothiophene	Gonzalez-Gaya 2016	6.7
PAHs	C1-Dibenzothiophene	Park et al. 2002	5.3
PAHs	C1-Dibenzothiophene	Gonzalez-Gaya 2016	5.8
PAHs	C1-Dibenzothiophene	This study	7.1
PAHs	C2-Dibenzothiophene	Park et al. 2002	5.1
PAHs	C2-Dibenzothiophene	This study	5.2
PAHs	C3-Dibenzothiophene	Park et al. 2002	5.0
PAHs	Phenanthrene	Poster & Baker 1996	6.0
PAHs	Phenanthrene	Franz & Eisenreich 1998	5.3
PAHs	Phenanthrene	Park et al. 2002	5.5
PAHs	Phenanthrene	Gonzalez-Gaya 2016	5.8
PAHs	Phenanthrene	Li et al. 2016	4.7
PAHs	Phenanthrene	IADN 1990-2019	5.4
PAHs	Phenanthrene	This study	6.1
PAHs	C1-Phenanthrene	Ligocki et al. 1985	4.1
PAHs	C1-Phenanthrene	Gonzalez-Gaya 2016	5.9
PAHs	C1-Phenanthrene	This study	5.9
PAHs	C2-Phenanthrene	Gonzalez-Gaya 2016	6.8
PAHs	C2-Phenanthrene	This study	5.1
PAHs	C3-Phenanthrene	This study	4.8
PAHs	Phenanthrene/Anthracene	Ligocki et al. 1985	4.2
PAHs	C1-Phenanthrene/Anthracene	Park et al. 2002	5.1
PAHs	C2-Phenanthrene/Anthracene	Park et al. 2002	5.0
PAHs	C3-Phenanthrene/Anthracene	Park et al. 2002	5.0
PAHs	C4-Phenanthrene/Anthracene	Park et al. 2002	5.0
PAHs	Anthracene	Poster & Baker 1996	6.1
PAHs	Anthracene	Franz & Eisenreich 1998	4.9
PAHs	Anthracene	Park et al. 2002	5.2
PAHs	Anthracene	Gonzalez-Gaya 2016	5.8
PAHs	Anthracene	Li et al. 2016	5.1
PAHs	Anthracene	IADN 1990-2019	5.3
PAHs	Anthracene	This study	5.3
PAHs	C1-Fluoranthrene/pyrene	Park et al. 2002	5.0
PAHs	C1-Fluoranthrene/pyrene	This study	4.3
PAHs	Fluoranthene	Ligocki et al. 1985	4.0
PAHs	Fluoranthene	Franz & Eisenreich 1998	4.9
PAHs	Fluoranthene	Gonzalez-Gaya 2016	5.1
PAHs	Fluoranthene	Li et al. 2016	4.3
PAHs	Fluoranthene	IADN 1990-2019	5.1
PAHs	Fluoranthene	This study	5.0
PAHs	Pyrene	Ligocki et al. 1985	4.0
PAHs	Pyrene	Poster & Baker 1996	5.9
PAHs	Pyrene	Franz & Eisenreich 1998	4.9
PAHs	Pyrene	Gonzalez-Gaya 2016	5.2
PAHs	Pyrene	IADN 1990-2019	5.0
PAHs	Pyrene	This study	5.0
PAHs	112Pyrene-C1	Gonzalez-Gaya 2016	4.9
PAHs	C1-Pyrene	Li et al. 2016	4.3

PAHs	C1-Pyrene	This study	5.1
PAHs	Retene	Franz & Eisenreich 1998	4.9
PAHs	Retene	IADN 1990-2019	4.9
PAHs	Benzo[g,h,i]fluoranthene	Gonzalez-Gaya 2016	5.8
PAHs	Benzo[g,h,i]fluoranthene	This study	5.0
PAHs	Benzo[a]anthracene	Ligocki et al. 1985	3.1
PAHs	Benzo[a]anthracene	Poster & Baker 1996	6.3
PAHs	Benzo[a]anthracene	Franz & Eisenreich 1998	4.1
PAHs	Benzo[a]anthracene	Park et al. 2002	5.1
PAHs	Benzo[a]anthracene	Gonzalez-Gaya 2016	5.2
PAHs	Benzo[a]anthracene	Li et al. 2016	3.9
PAHs	Benzo[a]anthracene	IADN 1990-2019	4.9
PAHs	Benzo[a]anthracene	This study	5.3
PAHs	Chrysene	Ligocki et al. 1985	3.4
PAHs	Chrysene	Poster & Baker 1996	6.2
PAHs	Chrysene	Franz & Eisenreich 1998	4.1
PAHs	Chrysene	Park et al. 2002	5.1
PAHs	Chrysene	Gonzalez-Gaya 2016	4.8
PAHs	Chrysene	Li et al. 2016	3.9
PAHs	Chrysene	This study	5.0
PAHs	Chrysene/Triphenylene	IADN 1990-2019	4.8
PAHs	C1-Chrysene	Park et al. 2002	4.7
PAHs	C1-Chrysene	Gonzalez-Gaya 2016	5.1
PAHs	C1-Chrysene	This study	4.6
PAHs	C2-Chrysene	Park et al. 2002	4.5
PAHs	C3-Chrysene	Park et al. 2002	4.5
PAHs	Benzo[b]fluoranthene	Poster & Baker 1996	6.2
PAHs	Benzo[b]fluoranthene	Park et al. 2002	5.1
PAHs	Benzo[b]fluoranthene	IADN 1990-2019	4.9
PAHs	Benzo[k]fluoranthene	Poster & Baker 1996	6.4
PAHs	Benzo[k]fluoranthene	Park et al. 2002	5.0
PAHs	Benzo[k]fluoranthene	Li et al. 2016	3.9
PAHs	Benzo[k]fluoranthene	IADN 1990-2019	4.8
PAHs	Benzo[b,k]fluoranthene	Franz & Eisenreich 1998	3.9
PAHs	Benzo[b,k]fluoranthene	Gonzalez-Gaya 2016	5.1
PAHs	Benzo[b,j,k]fluoranthene	Ligocki et al. 1985	3.3
PAHs	Benzo[e]pyrene	Ligocki et al. 1985	3.3
PAHs	Benzo[e]pyrene	Franz & Eisenreich 1998	3.9
PAHs	Benzo[e]pyrene	Park et al. 2002	5.1
PAHs	Benzo[e]pyrene	Gonzalez-Gaya 2016	4.7
PAHs	Benzo[e]pyrene	IADN 1990-2019	4.8
PAHs	Benzo[e]pyrene	Ligocki et al. 1985	3.2
PAHs	Benzo[e]pyrene	Franz & Eisenreich 1998	4.4
PAHs	Benzo[e]pyrene	Park et al. 2002	5.1
PAHs	Benzo[e]pyrene	Gonzalez-Gaya 2016	5.5
PAHs	Benzo[e]pyrene	Li et al. 2016	4.0
PAHs	Benzo[e]pyrene	IADN 1990-2019	4.9
PAHs	Perylene	Ligocki et al. 1985	3.3

PAHs	Perylene	Park et al. 2002	4.7
PAHs	Perylene	Gonzalez-Gaya 2016	6.1
PAHs	Indeno[1,2,3-cd]pyrene	Poster & Baker 1996	6.6
PAHs	Indeno[1,2,3-cd]pyrene	Franz & Eisenreich 1998	3.7
PAHs	Indeno[1,2,3-cd]pyrene	Park et al. 2002	5.0
PAHs	Indeno[1,2,3-cd]pyrene	Gonzalez-Gaya 2016	5.6
PAHs	Indeno[1,2,3-cd]pyrene	Li et al. 2016	3.8
PAHs	Indeno[1,2,3-cd]pyrene	IADN 1990-2019	4.9
PAHs	Dibenzo[a,h]anthracene	Franz & Eisenreich 1998	4.1
PAHs	Dibenzo[a,h]anthracene	Park et al. 2002	5.0
PAHs	Dibenzo[a,h]anthracene	Gonzalez-Gaya 2016	6.7
PAHs	Dibenzo[a,h]anthracene	Li et al. 2016	4.6
PAHs	Dibenzo[a,h]anthracene	IADN 1990-2019	4.9
PAHs	Benzo[g,h,i]perylene	Ligocki et al. 1985	3.5
PAHs	Benzo[g,h,i]perylene	Poster & Baker 1996	6.4
PAHs	Benzo[g,h,i]perylene	Franz & Eisenreich 1998	5.3
PAHs	Benzo[g,h,i]perylene	Park et al. 2002	5.0
PAHs	Benzo[g,h,i]perylene	Gonzalez-Gaya 2016	5.4
PAHs	Benzo[g,h,i]perylene	Li et al. 2016	3.5
PAHs	Benzo[g,h,i]perylene	IADN 1990-2019	4.8
PAHs	Coronene	Ligocki et al. 1985	3.8
PAHs	Coronene	IADN 1990-2019	4.6
PAHs	ΣPAHs	Holoubek et al. 2007	4.6
PAHs	ΣPAHs	Birgül et al. 2010	5.9
PAHs	ΣPAHs	Liu et al. 2013	5.5
PAHs	ΣPAHs	Li et al. 2016	4.3
PAHs	ΣPAHs	IADN 1990-2019	5.1
PAHs	Dibenzofuran	Ligocki et al. 1985	4.0
PAHs	9-Fluorenone	Ligocki et al. 1985	4.2
PAHs	9,10-Anthracenedione	Ligocki et al. 1985	3.4
PBDEs	BDE-3	Noël et al. 2009	5.3
PBDEs	BDE-15	Guo et al. 2014	5.0
PBDEs	BDE-17	Guo et al. 2014	4.5
PBDEs	BDE-28	Mariani et al. 2008	5.1
PBDEs	BDE-28	Guo et al. 2014	4.8
PBDEs	BDE-47	Venier & Hites 2008	5.6
PBDEs	BDE-47	Mariani et al. 2008	4.0
PBDEs	BDE-47	Noël et al. 2009	5.1
PBDEs	BDE-47	Guo et al. 2014	4.6
PBDEs	BDE-66	Guo et al. 2014	4.4
PBDEs	BDE-77	Guo et al. 2014	4.0
PBDEs	BDE-99	Mariani et al. 2008	4.5
PBDEs	BDE-99	Noël et al. 2009	5.0
PBDEs	BDE-99	Guo et al. 2014	4.7
PBDEs	BDE-100	Mariani et al. 2008	4.3
PBDEs	BDE-126	Guo et al. 2014	5.4
PBDEs	BDE-138	Guo et al. 2014	4.8
PBDEs	BDE-153	Mariani et al. 2008	5.2

PBDEs	BDE-153	Guo et al. 2014	4.8
PBDEs	BDE-154	Mariani et al. 2008	5.0
PBDEs	BDE-154	Guo et al. 2014	4.8
PBDEs	BDE-181	Guo et al. 2014	5.1
PBDEs	BDE-183	Mariani et al. 2008	5.6
PBDEs	BDE-183	Guo et al. 2014	4.8
PBDEs	BDE-190	Guo et al. 2014	4.7
PBDEs	BDE-196	Guo et al. 2014	4.8
PBDEs	BDE-203	Guo et al. 2014	4.8
PBDEs	BDE-204	Guo et al. 2014	4.6
PBDEs	BDE-206	Guo et al. 2014	4.3
PBDEs	BDE-207	Noël et al. 2009	5.6
PBDEs	BDE-207	Guo et al. 2014	4.5
PBDEs	BDE-208	Guo et al. 2014	4.5
PBDEs	BDE-209	Venier & Hites 2008	5.2
PBDEs	BDE-209	Mariani et al. 2008	5.1
PBDEs	BDE-209	Noël et al. 2009	6.0
PBDEs	BDE-209	Guo et al. 2014	4.7
PBDEs	ΣBDE	Venier & Hites 2008	5.5
PBDEs	ΣBDE	Mariani et al. 2008	4.7
PBDEs	ΣBDE	Noël et al. 2009	5.6
OPEs	EHDPP	This study	4.4
OPEs	TEP	This study	6.5
OPEs	TiBP	This study	5.5
OPEs	TNBP	This study	4.8
OPEs	TCEP	This study	6.1
OPEs	TCPP-1	This study	6.2
OPEs	TCPP-2	This study	6.2
OPEs	TCPP-3	This study	6.4
OPEs	TDCIPP	This study	6.7
PFAS	PFBS	Dreyer et al. 2010	5.9
PFAS	PFBS	This study	6.1
PFAS	PFOS	Dreyer et al. 2010	5.9
PFAS	PFOS	This study	6.3
PFAS	PFBA	Dreyer et al. 2010	6.9
PFAS	PFBA	This study	6.4
PFAS	PFHxA	Dreyer et al. 2010	6.4
PFAS	PFHxA	This study	6.3
PFAS	PFHpA	This study	6.5
PFAS	PFOA	Barton et al. 2007	5.2
PFAS	PFOA	Dreyer et al. 2010	6.7
PFAS	PFOA	This study	6.6
PFAS	PFNA	Dreyer et al. 2010	6.8
PFAS	PFNA	This study	7.0
PFAS	PFDA	Dreyer et al. 2010	6.8
PFAS	PFDA	This study	6.7
PFAS	PFUnDA	Dreyer et al. 2010	6.3
PFAS	PFUnDA	This study	7.0

PFAS	PFDoDA	This study	7.2
PFAS	MeFOSE	Dreyer et al. 2010	6.2
PFAS	EtFOSE	Dreyer et al. 2010	6.3

Table S6. K_{RG} mean for each compound and for each data set in the meta-analysis. The compound order is the same as in Figure 2.

Compound family	Compound	Reference	log K_{RG} Mean
PCB	Biphenyl	Park et al. 2002	3.9
PCB	PCB-4+10	IADN 1990-2019	3.4
PCB	PCB-4+15	Franz & Eisenreich 1998	3.9
PCB	PCB-5+8	Mandalakis & Stephanou 2004	4.5
PCB	PCB-5+8	IADN 1990-2019	3.2
PCB	PCB-6	Mandalakis & Stephanou 2004	4.5
PCB	PCB-6	IADN 1990-2019	3.5
PCB	PCB-7+9	IADN 1990-2019	3.0
PCB	PCB-8	Noël et al. 2009	5.1
PCB	PCB-11	IADN 1990-2019	2.0
PCB	PCB-12+13	Mandalakis & Stephanou 2004	4.3
PCB	PCB-15+17	IADN 1990-2019	3.1
PCB	PCB-16	IADN 1990-2019	3.3
PCB	PCB-16+39	Franz & Eisenreich 1998	3.2
PCB	PCB-16+39	Mandalakis & Stephanou 2004	4.4
PCB	PCB-17	Duniker & Bouchertall 1989	2.9
PCB	PCB-18	Duniker & Bouchertall 1989	2.9
PCB	PCB-18	Mandalakis & Stephanou 2004	4.4
PCB	PCB-18	Noël et al. 2009	4.2
PCB	PCB-18	IADN 1990-2019	3.1
PCB	PCB-19	IADN 1990-2019	2.9
PCB	9PCB-22	Mandalakis & Stephanou 2004	4.7
PCB	9PCB-22	IADN 1990-2019	3.3
PCB	PCB-24+27	Mandalakis & Stephanou 2004	4.2
PCB	PCB-26	Duniker & Bouchertall 1989	3.6
PCB	PCB-26	IADN 1990-2019	3.5
PCB	PCB-27	Duniker & Bouchertall 1989	3.4
PCB	PCB-28	Granier & Chevreuil 1997	4.3
PCB	PCB-28	Mandalakis & Stephanou 2004	4.6
PCB	PCB-28	Blanchard et al. 2006	4.6
PCB	PCB-28	Noël et al. 2009	4.4
PCB	PCB-28	IADN 1990-2019	3.1
PCB	PCB-31	Mandalakis & Stephanou 2004	4.5
PCB	PCB-31	Noël et al. 2009	4.3
PCB	PCB-31	IADN 1990-2019	3.2
PCB	PCB-32	IADN 1990-2019	3.1
PCB	PCB-33	Backe et al. 2002	3.2
PCB	PCB-33	Noël et al. 2009	5.9
PCB	PCB-33	IADN 1990-2019	3.1

PCB	PCB-33+20	Mandalakis & Stephanou 2004	4.6
PCB	PCB-37	IADN 1990-2019	3.3
PCB	PCB-40+81	Franz & Eisenreich 1998	3.8
PCB	PCB-41+46	Mandalakis & Stephanou 2004	4.3
PCB	PCB-41+71	IADN 1990-2019	3.0
PCB	PCB-42	IADN 1990-2019	3.2
PCB	PCB-44	Duniker & Bouchertall 1989	3.2
PCB	PCB-44	Mandalakis & Stephanou 2004	4.3
PCB	PCB-45	IADN 1990-2019	3.0
PCB	PCB-47	IADN 1990-2019	3.3
PCB	PCB-47+48	Mandalakis & Stephanou 2004	4.4
PCB	PCB-48	IADN 1990-2019	3.1
PCB	PCB-49	Duniker & Bouchertall 1989	3.2
PCB	PCB-49	Mandalakis & Stephanou 2004	4.3
PCB	PCB-49	IADN 1990-2019	2.9
PCB	PCB-52	Duniker & Bouchertall 1989	3.6
PCB	PCB-52	Granier & Chevreuil 1997	4.8
PCB	PCB-52	Backe et al. 2002	4.2
PCB	PCB-52	Mandalakis & Stephanou 2004	4.2
PCB	PCB-52	Blanchard et al. 2006	4.7
PCB	PCB-52	IADN 1990-2019	2.9
PCB	PCB-53	IADN 1990-2019	3.1
PCB	PCB-56+60	IADN 1990-2019	3.2
PCB	PCB-60	Granier & Chevreuil 1997	4.7
PCB	PCB-64	IADN 1990-2019	3.1
PCB	PCB-66	Mandalakis & Stephanou 2004	4.1
PCB	PCB-66	IADN 1990-2019	3.1
PCB	PCB-70	Mandalakis & Stephanou 2004	4.2
PCB	PCB-70+76	IADN 1990-2019	3.0
PCB	PCB-74	Mandalakis & Stephanou 2004	4.1
PCB	PCB-74	IADN 1990-2019	3.1
PCB	PCB-81	IADN 1990-2019	3.7
PCB	PCB-82+127	Franz & Eisenreich 1998	4.2
PCB	PCB-83	IADN 1990-2019	3.2
PCB	PCB-87	IADN 1990-2019	3.2
PCB	PCB-89	IADN 1990-2019	3.3
PCB	PCB-90+101	Mandalakis & Stephanou 2004	4.0
PCB	PCB-91	Mandalakis & Stephanou 2004	3.9
PCB	PCB-91	IADN 1990-2019	3.2
PCB	PCB-92+84	IADN 1990-2019	3.1
PCB	PCB-95	Mandalakis & Stephanou 2004	4.0
PCB	PCB-95	IADN 1990-2019	2.9
PCB	PCB-97	IADN 1990-2019	3.2
PCB	PCB-99	Mandalakis & Stephanou 2004	3.8
PCB	PCB-99	IADN 1990-2019	3.1
PCB	PCB-100	IADN 1990-2019	3.5
PCB	PCB-101	Duniker & Bouchertall 1989	3.5
PCB	PCB-101	Granier & Chevreuil 1997	4.8

PCB	PCB-101	Backe et al. 2002	4.4
PCB	PCB-101	Agrell et al. 2002	4.9
PCB	PCB-101	TerSchure et al. 2004a	4.6
PCB	PCB-101	Blanchard et al. 2006	4.4
PCB	PCB-101	IADN 1990-2019	3.1
PCB	PCB-118	Duniker & Bouchertall 1989	3.8
PCB	PCB-118	Backe et al. 2002	4.5
PCB	PCB-118	Blanchard et al. 2006	4.5
PCB	PCB-119	IADN 1990-2019	4.2
PCB	PCB-128+169	Franz & Eisenreich 1998	3.9
PCB	PCB-132	Mandalakis & Stephanou 2004	4.4
PCB	PCB-138	Duniker & Bouchertall 1989	4.3
PCB	PCB-138	Granier & Chevreuil 1997	5.1
PCB	PCB-138	Backe et al. 2002	4.7
PCB	PCB-138	TerSchure et al. 2004a	5.0
PCB	PCB-138	Blanchard et al. 2006	4.1
PCB	PCB-138+163+165	Mandalakis & Stephanou 2004	4.6
PCB	PCB-149	Duniker & Bouchertall 1989	3.9
PCB	PCB-153	Duniker & Bouchertall 1989	4.1
PCB	PCB-153	Granier & Chevreuil 1997	5.1
PCB	PCB-153	Backe et al. 2002	4.6
PCB	PCB-153	Mandalakis & Stephanou 2004	4.3
PCB	PCB-158+160	Mandalakis & Stephanou 2004	4.9
PCB	PCB-170+190	Franz & Eisenreich 1998	3.1
PCB	PCB-170+190	Mandalakis & Stephanou 2004	4.8
PCB	PCB-174	Mandalakis & Stephanou 2004	4.7
PCB	PCB-177	Duniker & Bouchertall 1989	4.3
PCB	PCB-180	Duniker & Bouchertall 1989	4.6
PCB	PCB-180	Granier & Chevreuil 1997	5.4
PCB	PCB-180	Backe et al. 2002	5.0
PCB	PCB-180	Agrell et al. 2002	4.5
PCB	PCB-180	Mandalakis & Stephanou 2004	5.1
PCB	PCB-180	TerSchure et al. 2004a	5.4
PCB	PCB-194	Mandalakis & Stephanou 2004	5.1
PCB	PCB-194	TerSchure et al. 2004a	5.6
PCB	PCB-194+205	Franz & Eisenreich 1998	3.4
PCB	PCB-196+203	Mandalakis & Stephanou 2004	4.9
PCB	ΣPCB	Bidelman & Christensen 1979	4.6
PCB	ΣPCB	Atlas & Giam1987	3.9
PCB	ΣPCB	Granier & Chevreuil 1997	4.5
PCB	ΣPCB	VanRy et al. 2002	3.6
PCB	ΣPCB	Offenberg & Baker 2002	4.6
PCB	ΣPCB	Agrell et al. 2002	4.6
PCB	ΣPCB	TerSchure et al. 2004a	4.7
PCB	ΣPCB	Holoubek et al. 2007	4.5
PCB	ΣPCB	Noël et al. 2009	5.7
PCB	ΣPCB	Günindi et al. 2011	4.9
OCPs	α-HCH	Atlas & Giam 1981	4.0

OCPs	α-HCH	Pankow et al. 1984	4.1
OCPs	α-HCH	Atlas & Giam1987	4.4
OCPs	α-HCH	Wania & Haugen 1999	4.5
OCPs	α-HCH	Park et al. 2002	4.1
OCPs	α-HCH	Takase et al. 2003	3.7
OCPs	α-HCH	IADN 1990-2019	3.7
OCPs	γ-HCH	Atlas & Giam 1981	4.4
OCPs	γ-HCH	Pankow et al. 1984	4.5
OCPs	γ-HCH	Atlas & Giam1987	4.6
OCPs	γ-HCH	Wania & Haugen 1999	5.0
OCPs	γ-HCH	Park et al. 2002	3.8
OCPs	γ-HCH	IADN 1990-2019	4.2
OCPs	β-HCH	IADN 1990-2019	4.8
OCPs	ΣHCH	Holoubek et al. 2007	5.7
OCPs	HCB	Park et al. 2002	3.4
OCPs	HCB	Takase et al. 2003	2.7
OCPs	HCB	Holoubek et al. 2007	3.0
OCPs	HCB	IADN 1990-2019	2.2
OCPs	1,2,4,5-TCB	Park et al. 2002	3.9
OCPs	1,2,3,4-TCB	Park et al. 2002	5.1
OCPs	DDE	Park et al. 2002	3.0
OCPs	DDE	IADN 1990-2019	3.2
OCPs	DDD	IADN 1990-2019	4.7
OCPs	DDT	Park et al. 2002	3.0
OCPs	DDT	Takase et al. 2003	3.6
OCPs	DDT	Gioia et al.2005	3.5
OCPs	DDT	Holoubek et al. 2007	4.8
OCPs	DDT	IADN 1990-2019	3.5
OCPs	Chlordanes	Gioia et al.2005	2.8
OCPs	Heptachlor	Park et al. 2002	4.1
OCPs	Heptachlor	IADN 1990-2019	3.6
OCPs	Oxychlordane	IADN 1990-2019	3.5
OCPs	α-Chlordane	Park et al. 2002	3.4
OCPs	α-Chlordane	IADN 1990-2019	3.3
OCPs	γ-Chlordane	Park et al. 2002	3.3
OCPs	γ-Chlordane	IADN 1990-2019	3.4
OCPs	Trans-nonachlor	Park et al. 2002	3.8
OCPs	Trans-nonachlor	IADN 1990-2019	3.0
OCPs	Pentachloroanisole	Park et al. 2002	3.5
OCPs	Dieldrin	Park et al. 2002	3.9
OCPs	Dieldrin	IADN 1990-2019	3.8
OCPs	Endrin	IADN 1990-2019	4.6
OCPs	Chlorpyrifos	Park et al. 2002	3.7
OCPs	Endosulfan I	Gioia et al.2005	3.4
OCPs	Endosulfan I	IADN 1990-2019	3.9
OCPs	Endosulfan II	Gioia et al.2005	5.1
OCPs	Endosulfan II	IADN 1990-2019	5.2
OCPs	Endosulfansulfate	IADN 1990-2019	5.6

PCDDFs	5-PCDD	Eitzer & Hites 1989	3.8
PCDDFs	5-PCDD	Koester & Hites 1992	4.1
PCDDFs	6-PCDD	Eitzer & Hites 1989	3.7
PCDDFs	6-PCDD	Koester & Hites 1992	4.2
PCDDFs	7-PCDD	Eitzer & Hites 1989	5.4
PCDDFs	7-PCDD	Koester & Hites 1992	5.2
PCDDFs	8-PCDD	Eitzer & Hites 1989	6.4
PCDDFs	8-PCDD	Koester & Hites 1992	6.2
PCDDFs	4-PCDF	Eitzer & Hites 1989	4.2
PCDDFs	4-PCDF	Koester & Hites 1992	4.4
PCDDFs	5-PCDF	Eitzer & Hites 1989	4.0
PCDDFs	5-PCDF	Koester & Hites 1992	4.2
PCDDFs	6-PCDF	Eitzer & Hites 1989	3.9
PCDDFs	6-PCDF	Koester & Hites 1992	4.0
PCDDFs	7-PCDF	Eitzer & Hites 1989	4.8
PCDDFs	7-PCDF	Koester & Hites 1992	4.6
PCDDFs	8-PCDF	Eitzer & Hites 1989	5.3
PCDDFs	8-PCDF	Koester & Hites 1992	5.0
PAHs	Naphthalene	Park et al. 2002	6.1
PAHs	Naphthalene	Gonzalez-Gaya 2016	6.6
PAHs	C1-Naphthalene	Park et al. 2002	3.8
PAHs	C2-Naphthalene	Park et al. 2002	3.5
PAHs	C2-Naphthalene	Gonzalez-Gaya 2016	7.0
PAHs	C3-Naphthalene	Park et al. 2002	3.5
PAHs	C4-Naphthalene	Park et al. 2002	3.4
PAHs	Acenaphthylene	Franz & Eisenreich 1998	2.7
PAHs	Acenaphthylene	Park et al. 2002	3.9
PAHs	Acenaphthylene	Gonzalez-Gaya 2016	5.6
PAHs	Acenaphthylene	Li et al. 2016	4.5
PAHs	Acenaphthene	Franz & Eisenreich 1998	3.0
PAHs	Acenaphthene	Park et al. 2002	3.8
PAHs	Acenaphthene	Gonzalez-Gaya 2016	4.7
PAHs	Acenaphthene	Li et al. 2016	5.2
PAHs	Fluorene	Ligocki et al. 1985	3.2
PAHs	Fluorene	Poster & Baker 1996	1.8
PAHs	Fluorene	Franz & Eisenreich 1998	3.1
PAHs	Fluorene	Park et al. 2002	3.4
PAHs	Fluorene	Tsapakis et al. 2006	3.5
PAHs	Fluorene	Gonzalez-Gaya 2016	4.4
PAHs	Fluorene	Li et al. 2016	4.5
PAHs	Fluorene	IADN 1990-2019	2.9
PAHs	C1-Fluorene	Park et al. 2002	3.9
PAHs	C2-Fluorene	Park et al. 2002	3.2
PAHs	C3-Fluorene	Park et al. 2002	3.5
PAHs	Benzo[b]fluorene	Li et al. 2016	4.8
PAHs	Dibenzothiophene	Park et al. 2002	3.5
PAHs	094Dibenzothiophene	Gonzalez-Gaya 2016	4.2
PAHs	C1-Dibenzothiophene	Park et al. 2002	3.2

PAHs	C1-Dibenzothiophene	Gonzalez-Gaya 2016	3.8
PAHs	C2-Dibenzothiophene	Park et al. 2002	3.2
PAHs	C3-Dibenzothiophene	Park et al. 2002	3.3
PAHs	Phenanthrene	Poster & Baker 1996	2.4
PAHs	Phenanthrene	Franz & Eisenreich 1998	3.5
PAHs	Phenanthrene	Park et al. 2002	3.4
PAHs	Phenanthrene	Tsapakis et al. 2006	4.1
PAHs	Phenanthrene	Gonzalez-Gaya 2016	3.4
PAHs	Phenanthrene	Li et al. 2016	3.9
PAHs	Phenanthrene	IADN 1990-2019	3.5
PAHs	C1-Phenanthrene	Ligocki et al. 1985	3.4
PAHs	C1-Phenanthrene	Tsapakis et al. 2006	4.1
PAHs	C1-Phenanthrene	Gonzalez-Gaya 2016	3.8
PAHs	C2-Phenanthrene	Gonzalez-Gaya 2016	5.1
PAHs	Phenanthrene/Anthracene	Ligocki et al. 1985	3.5
PAHs	C1-Phenanthrene/Anthracene	Park et al. 2002	3.2
PAHs	C2-Phenanthrene/Anthracene	Park et al. 2002	3.3
PAHs	C3-Phenanthrene/Anthracene	Park et al. 2002	3.0
PAHs	C4-Phenanthrene/Anthracene	Park et al. 2002	3.3
PAHs	Anthracene	Poster & Baker 1996	1.1
PAHs	Anthracene	Franz & Eisenreich 1998	3.2
PAHs	Anthracene	Park et al. 2002	3.5
PAHs	Anthracene	Tsapakis et al. 2006	4.1
PAHs	Anthracene	Gonzalez-Gaya 2016	3.5
PAHs	Anthracene	Li et al. 2016	4.2
PAHs	Anthracene	IADN 1990-2019	3.8
PAHs	C1-Fluoranthrene/Pyrene	Park et al. 2002	3.9
PAHs	Fluoranthene	Ligocki et al. 1985	3.8
PAHs	Fluoranthene	Franz & Eisenreich 1998	3.9
PAHs	Fluoranthene	Tsapakis et al. 2006	4.9
PAHs	Fluoranthene	Gonzalez-Gaya 2016	3.8
PAHs	Fluoranthene	Li et al. 2016	3.9
PAHs	Fluoranthene	IADN 1990-2019	4.1
PAHs	Pyrene	Ligocki et al. 1985	3.8
PAHs	Pyrene	Poster & Baker 1996	2.6
PAHs	Pyrene	Franz & Eisenreich 1998	4.0
PAHs	Pyrene	Tsapakis et al. 2006	4.7
PAHs	Pyrene	Gonzalez-Gaya 2016	4.0
PAHs	Pyrene	IADN 1990-2019	4.3
PAHs	C1-Pyrene	Gonzalez-Gaya 2016	4.2
PAHs	C1-Pyrene	Li et al. 2016	4.3
PAHs	Retene	Franz & Eisenreich 1998	4.1
PAHs	Retene	IADN 1990-2019	4.2
PAHs	Benzo[g,h,i]fluoranthene	Gonzalez-Gaya 2016	4.8
PAHs	Benzo[a]anthracene	Ligocki et al. 1985	4.1
PAHs	Benzo[a]anthracene	Poster & Baker 1996	2.7
PAHs	Benzo[a]anthracene	Franz & Eisenreich 1998	4.7
PAHs	Benzo[a]anthracene	Park et al. 2002	4.9

PAHs	Benzo[a]anthracene	Tsapakis et al. 2006	5.2
PAHs	Benzo[a]anthracene	Gonzalez-Gaya 2016	5.0
PAHs	Benzo[a]anthracene	Li et al. 2016	5.3
PAHs	Benzo[a]anthracene	IADN 1990-2019	5.5
PAHs	Chrysene	Ligocki et al. 1985	4.3
PAHs	Chrysene	Poster & Baker 1996	2.1
PAHs	Chrysene	Franz & Eisenreich 1998	4.7
PAHs	Chrysene	Park et al. 2002	4.8
PAHs	Chrysene	Gonzalez-Gaya 2016	4.5
PAHs	Chrysene	Li et al. 2016	5.1
PAHs	Chrysene/Triphenylene	Tsapakis et al. 2006	5.4
PAHs	Chrysene/Triphenylene	IADN 1990-2019	5.1
PAHs	C1-Chrysene	Park et al. 2002	4.4
PAHs	C1-Chrysene	Gonzalez-Gaya 2016	5.2
PAHs	C2-Chrysene	Park et al. 2002	4.2
PAHs	C3-Chrysene	Park et al. 2002	3.9
PAHs	C4-Chrysene	Park et al. 2002	4.8
PAHs	Benzo[b]fluoranthene	Poster & Baker 1996	2.7
PAHs	Benzo[b]fluoranthene	Park et al. 2002	5.4
PAHs	Benzo[b]fluoranthene	IADN 1990-2019	5.7
PAHs	Benzo[k]fluoranthene	Poster & Baker 1996	2.4
PAHs	Benzo[k]fluoranthene	Park et al. 2002	5.3
PAHs	Benzo[k]fluoranthene	Li et al. 2016	5.1
PAHs	Benzo[k]fluoranthene	IADN 1990-2019	5.7
PAHs	Benzo[b,k]fluoranthene	Franz & Eisenreich 1998	5.6
PAHs	Benzo[b,k]fluoranthene	Gonzalez-Gaya 2016	5.2
PAHs	Benzo[b,j,k]fluoranthene	Ligocki et al. 1985	3.9
PAHs	Benzo[e]pyrene	Ligocki et al. 1985	3.8
PAHs	Benzo[e]pyrene	Franz & Eisenreich 1998	5.9
PAHs	Benzo[e]pyrene	Park et al. 2002	5.3
PAHs	Benzo[e]pyrene	Gonzalez-Gaya 2016	5.2
PAHs	Benzo[e]pyrene	IADN 1990-2019	5.6
PAHs	Benzo[a]pyrene	Park et al. 2002	5.3
PAHs	Benzo[a]pyrene	Gonzalez-Gaya 2016	5.5
PAHs	Benzo[a]pyrene	IADN 1990-2019	5.7
PAHs	Perylene	Park et al. 2002	4.6
PAHs	Perylene	Gonzalez-Gaya 2016	5.7
PAHs	Indeno[1,2,3-cd]pyrene	Franz & Eisenreich 1998	5.7
PAHs	Indeno[1,2,3-cd]pyrene	Park et al. 2002	5.5
PAHs	Indeno[1,2,3-cd]pyrene	Gonzalez-Gaya 2016	6.3
PAHs	Indeno[1,2,3-cd]pyrene	IADN 1990-2019	5.6
PAHs	Dibenzo[a,h]anthracene	Park et al. 2002	4.8
PAHs	Dibenzo[a,h]anthracene	Gonzalez-Gaya 2016	6.6
PAHs	Dibenzo[a,h]anthracene	Li et al. 2016	5.6
PAHs	Benzo[g,h,i]perylene	Franz & Eisenreich 1998	7.3
PAHs	Benzo[g,h,i]perylene	Park et al. 2002	5.4
PAHs	Benzo[g,h,i]perylene	Gonzalez-Gaya 2016	7.0
PAHs	Benzo[g,h,i]perylene	Li et al. 2016	5.7

PAHs	Benzo[g,h,i]perylene	IADN 1990-2019	5.7
PAHs	Coronene	IADN 1990-2019	5.4
PAHs	ΣPAHs	Dickhut & Gustafson 1995	3.3
PAHs	ΣPAHs	Holoubek et al. 2007	4.3
PAHs	ΣPAHs	Birgül et al. 2010	6.0
PAHs	ΣPAHs	Li et al. 2016	4.3
PAHs	ΣPAHs	IADN 1990-2019	3.9
PAHs	Dibenzofuran	Ligocki et al. 1985	3.0
PAHs	9-Fluorenone	Ligocki et al. 1985	4.0
PAHs	9,10-Anthracenedione	Ligocki et al. 1985	4.4
PBDEs	BDE-28	Mariani et al. 2008	3.5
PBDEs	BDE-47	Venier & Hites 2008	5.2
PBDEs	BDE-47	Mariani et al. 2008	3.4
PBDEs	BDE-47	Noël et al. 2009	4.4
PBDEs	BDE-47	Guo et al. 2014	4.7
PBDEs	BDE-66	Guo et al. 2014	4.2
PBDEs	BDE-77	Guo et al. 2014	3.8
PBDEs	BDE-99	Mariani et al. 2008	5.0
PBDEs	BDE-99	Noël et al. 2009	4.2
PBDEs	BDE-99	Guo et al. 2014	4.3
PBDEs	BDE-100	Mariani et al. 2008	4.6
PBDEs	BDE-126	Guo et al. 2014	5.7
PBDEs	BDE-138	Guo et al. 2014	5.2
PBDEs	BDE-153	Mariani et al. 2008	6.1
PBDEs	BDE-153	Guo et al. 2014	5.3
PBDEs	BDE-154	Mariani et al. 2008	6.0
PBDEs	BDE-154	Guo et al. 2014	5.5
PBDEs	BDE-181	Guo et al. 2014	5.5
PBDEs	BDE-183	Mariani et al. 2008	5.9
PBDEs	BDE-183	Guo et al. 2014	5.3
PBDEs	BDE-190	Guo et al. 2014	5.1
PBDEs	BDE-196	Guo et al. 2014	4.9
PBDEs	BDE-203	Guo et al. 2014	4.9
PBDEs	BDE-204	Guo et al. 2014	4.8
PBDEs	BDE-206	Guo et al. 2014	4.7
PBDEs	BDE-207	Guo et al. 2014	4.7
PBDEs	BDE-208	Guo et al. 2014	5.0
PBDEs	BDE-209	Venier & Hites 2008	6.0
PBDEs	BDE-209	Mariani et al. 2008	6.1
PBDEs	BDE-209	Noël et al. 2009	6.8
PBDEs	BDE-209	Guo et al. 2014	5.4
PBDEs	ΣBDE	Venier & Hites 2008	5.6
PBDEs	ΣBDE	Mariani et al. 2008	4.2
PBDEs	ΣBDE	Noël et al. 2009	5.1
PFAS	MeFOSE	Dreyer et al. 2010	5.4
PFAS	EtFOSE	Dreyer et al. 2010	5.3
PFAS	MeFBSE	Dreyer et al. 2010	5.3

Table S7. K_{RA} mean for each compound and for each data set in the meta-analysis. The compound order is the same as in Figure 3.

Compound family	Compound	Reference	log K_{RA} Mean
PCB	Biphenyl	Park et al. 2002	3.9
PCB	PCB-4+10	IADN 1990-2019	3.4
PCB	PCB-4+-15	Franz & Eisenreich 1998	3.8
PCB	PCB-5+8	Mandalakis & Stephanou 2004	4.5
PCB	PCB-5+8	IADN 1990-2019	3.205
PCB	PCB-6	Mandalakis & Stephanou 2004	4.544
PCB	PCB-6	IADN 1990-2019	3.514
PCB	PCB-7+9	IADN 1990-2019	2.995
PCB	PCB-8	Noël et al. 2009	5.063
PCB	PCB-11	IADN 1990-2019	1.95
PCB	PCB-12+13	Mandalakis & Stephanou 2004	4.301
PCB	PCB-15+17	IADN 1990-2019	3.121
PCB	PCB-16	IADN 1990-2019	3.331
PCB	PCB-16+39	Franz & Eisenreich 1998	3.167
PCB	PCB-16+39	Mandalakis & Stephanou 2004	4.447
PCB	PCB-17	Duniker & Bouchertall 1989	2.907
PCB	PCB-18	Duniker & Bouchertall 1989	2.936
PCB	PCB-18	Mandalakis & Stephanou 2004	4.38
PCB	PCB-18	Noël et al. 2009	5.314
PCB	PCB-18	IADN 1990-2019	3.102
PCB	PCB-19	IADN 1990-2019	2.927
PCB	PCB-22	Mandalakis & Stephanou 2004	4.672
PCB	PCB-22	IADN 1990-2019	3.313
PCB	PCB-24+27	Mandalakis & Stephanou 2004	4.204
PCB	PCB-26	Duniker & Bouchertall 1989	3.578
PCB	PCB-26	IADN 1990-2019	3.535
PCB	PCB-27	Duniker & Bouchertall 1989	3.388
PCB	PCB-28	Granier & Chevreuil 1997	4.322
PCB	PCB-28	Mandalakis & Stephanou 2004	4.556
PCB	PCB-28	Blanchard et al. 2006	4.632
PCB	PCB-28	Noël et al. 2009	5.53
PCB	PCB-28	IADN 1990-2019	3.112
PCB	PCB-31	Mandalakis & Stephanou 2004	4.505
PCB	PCB-31	Noël et al. 2009	5.448
PCB	PCB-31	IADN 1990-2019	3.171
PCB	PCB-32	IADN 1990-2019	3.063
PCB	PCB-33	Backe et al. 2002	3.204
PCB	PCB-33	Noël et al. 2009	5.854
PCB	PCB-33	IADN 1990-2019	3.091
PCB	PCB-33+20	Mandalakis & Stephanou 2004	4.591
PCB	PCB-37	IADN 1990-2019	3.329
PCB	PCB-40+81	Franz & Eisenreich 1998	3.771
PCB	PCB-41+46	Mandalakis & Stephanou 2004	4.322
PCB	PCB-41+71	IADN 1990-2019	3.0

PCB	PCB-42	IADN 1990-2019	3.2
PCB	PCB-44	Duniker & Bouchertall 1989	3.2
PCB	PCB-44	Mandalakis & Stephanou 2004	4.3
PCB	PCB-45	IADN 1990-2019	3.0
PCB	PCB-47	IADN 1990-2019	3.3
PCB	PCB-47+48	Mandalakis & Stephanou 2004	4.4
PCB	PCB-48	IADN 1990-2019	3.1
PCB	PCB-49	Duniker & Bouchertall 1989	3.2
PCB	PCB-49	Mandalakis & Stephanou 2004	4.3
PCB	PCB-49	IADN 1990-2019	2.9
PCB	PCB-52	Duniker & Bouchertall 1989	3.6
PCB	PCB-52	Granier & Chevreuil 1997	4.8
PCB	PCB-52	Backe et al. 2002	4.2
PCB	PCB-52	Mandalakis & Stephanou 2004	4.2
PCB	PCB-52	Blanchard et al. 2006	4.7
PCB	PCB-52	IADN 1990-2019	2.9
PCB	PCB-53	IADN 1990-2019	3.1
PCB	PCB-56+60	IADN 1990-2019	3.2
PCB	PCB-60	Granier & Chevreuil 1997	4.7
PCB	PCB-64	IADN 1990-2019	3.1
PCB	PCB-66	Mandalakis & Stephanou 2004	4.1
PCB	PCB-66	IADN 1990-2019	3.1
PCB	PCB-70	Mandalakis & Stephanou 2004	4.2
PCB	PCB-70+76	IADN 1990-2019	3.0
PCB	PCB-74	Mandalakis & Stephanou 2004	4.1
PCB	PCB-74	IADN 1990-2019	3.1
PCB	PCB-81	IADN 1990-2019	3.7
PCB	PCB-82+127	Franz & Eisenreich 1998	4.2
PCB	PCB-83	IADN 1990-2019	3.2
PCB	PCB-87	IADN 1990-2019	3.2
PCB	PCB-89	IADN 1990-2019	3.3
PCB	PCB-90+101	Mandalakis & Stephanou 2004	4.0
PCB	PCB-91	Mandalakis & Stephanou 2004	3.9
PCB	PCB-91	IADN 1990-2019	3.2
PCB	PCB-92+84	IADN 1990-2019	3.1
PCB	PCB-95	Mandalakis & Stephanou 2004	4.0
PCB	PCB-95	IADN 1990-2019	2.9
PCB	PCB-97	IADN 1990-2019	3.2
PCB	PCB-99	Mandalakis & Stephanou 2004	3.8
PCB	PCB-99	IADN 1990-2019	3.1
PCB	PCB-100	IADN 1990-2019	3.5
PCB	PCB-101	Duniker & Bouchertall 1989	3.5
PCB	PCB-101	Granier & Chevreuil 1997	4.8
PCB	PCB-101	Backe et al. 2002	4.4
PCB	PCB-101	Agrell et al. 2002	4.9
PCB	PCB-101	TerSchure et al. 2004a	4.6
PCB	PCB-101	Blanchard et al. 2006	4.4
PCB	PCB-101	IADN 1990-2019	3.1

PCB	PCB-118	Duniker & Bouchertall 1989	3.8
PCB	PCB-118	Backe et al. 2002	4.5
PCB	PCB-118	Blanchard et al. 2006	4.5
PCB	PCB-119	IADN 1990-2019	4.2
PCB	PCB-128+169	Franz & Eisenreich 1998	3.7
PCB	PCB-132	Mandalakis & Stephanou 2004	4.4
PCB	PCB-138	Duniker & Bouchertall 1989	4.2
PCB	PCB-138	Granier & Chevreuil 1997	5.1
PCB	PCB-138	Backe et al. 2002	4.7
PCB	PCB-138	TerSchure et al. 2004a	5.0
PCB	PCB-138	Blanchard et al. 2006	4.1
PCB	PCB-138+163+165	Mandalakis & Stephanou 2004	4.6
PCB	PCB-149	Duniker & Bouchertall 1989	3.9
PCB	PCB-153	Duniker & Bouchertall 1989	4.1
PCB	PCB-153	Granier & Chevreuil 1997	5.1
PCB	PCB-153	Backe et al. 2002	4.6
PCB	PCB-153	Mandalakis & Stephanou 2004	4.3
PCB	PCB-158+160	Mandalakis & Stephanou 2004	4.9
PCB	PCB-170+190	Franz & Eisenreich 1998	3.1
PCB	PCB-170+190	Mandalakis & Stephanou 2004	4.8
PCB	PCB-174	Mandalakis & Stephanou 2004	4.7
PCB	PCB-177	Duniker & Bouchertall 1989	4.3
PCB	PCB-180	Duniker & Bouchertall 1989	4.5
PCB	PCB-180	Granier & Chevreuil 1997	5.4
PCB	PCB-180	Backe et al. 2002	5.0
PCB	PCB-180	Agrell et al. 2002	4.5
PCB	PCB-180	Mandalakis & Stephanou 2004	5.1
PCB	PCB-180	TerSchure et al. 2004a	5.4
PCB	PCB-194	Mandalakis & Stephanou 2004	5.1
PCB	PCB-194	TerSchure et al. 2004a	5.6
PCB	PCB-194+205	Franz & Eisenreich 1998	3.3
PCB	PCB-196+203	Mandalakis & Stephanou 2004	4.9
PCB	ΣPCB	Bidelman & Christensen 1979	4.6
PCB	ΣPCB	Atlas & Giam1987	3.9
PCB	ΣPCB	Granier & Chevreuil 1997	4.5
PCB	ΣPCB	VanRy et al. 2002	3.6
PCB	ΣPCB	Offenberg & Baker 2002	4.6
PCB	ΣPCB	Agrell et al. 2002	4.6
PCB	ΣPCB	TerSchure et al. 2004a	4.7
PCB	ΣPCB	Holoubek et al. 2007	4.4
PCB	ΣPCB	Noël et al. 2009	5.3
PCB	ΣPCB	Günindi et al. 2011	4.9
OCPs	α-HCH	Atlas & Giam 1981	4.0
OCPs	α-HCH	Pankow et al. 1984	4.1
OCPs	α-HCH	Atlas & Giam1987	4.4
OCPs	α-HCH	Wania & Haugen 1999	4.5
OCPs	α-HCH	Park et al. 2002	4.1
OCPs	α-HCH	Takase et al. 2003	3.7

OCPs	α-HCH	IADN 1990-2019	3.7
OCPs	γ-HCH	Atlas & Giam 1981	4.4
OCPs	γ-HCH	Pankow et al. 1984	4.5
OCPs	γ-HCH	Atlas & Giam1987	4.6
OCPs	γ-HCH	Wania & Haugen 1999	5.0
OCPs	γ-HCH	Park et al. 2002	3.8
OCPs	γ-HCH	IADN 1990-2019	3.9
OCPs	β-HCH	IADN 1990-2019	4.3
OCPs	ΣHCH	Holoubek et al. 2007	5.6
OCPs	HCB	Atlas & Giam1987	3.3
OCPs	HCB	Park et al. 2002	3.4
OCPs	HCB	Holoubek et al. 2007	3.0
OCPs	1,2,4,5-TCB	Park et al. 2002	3.9
OCPs	1,2,3,4-TCB	Park et al. 2002	5.1
OCPs	DBP	Pankow et al. 1984	4.7
OCPs	DBP	Atlas & Giam1987	4.5
OCPs	DDE	Atlas & Giam1987	3.6
OCPs	DDD	IADN 1990-2019	5.5
OCPs	DDT	Bidelman & Christensen 1979	4.8
OCPs	DDT	Atlas & Giam 1981	4.1
OCPs	DDT	Atlas & Giam1987	4.7
OCPs	DDT	Park et al. 2002	2.5
OCPs	DDT	Gioia et al. 2005	3.5
OCPs	DDT	Holoubek et al. 2007	4.8
OCPs	DDT	IADN 1990-2019	4.0
OCPs	DEHP	Pankow et al. 1984	5.0
OCPs	DEHP	Atlas & Giam1987	5.0
OCPs	Chlordanes	Bidelman & Christensen 1979	4.1
OCPs	Chlordanes	Atlas & Giam1987	3.3
OCPs	Chlordanes	Gioia et al. 2005	2.8
OCPs	Heptachlor	Park et al. 2002	4.1
OCPs	Heptachlor	IADN 1990-2019	3.5
OCPs	Oxychlordane	Gioia et al. 2005	4.4
OCPs	Oxychlordane	IADN 1990-2019	4.1
OCPs	α-Chlordane	Park et al. 2002	3.4
OCPs	α-Chlordane	IADN 1990-2019	3.3
OCPs	γ-Chlordane	Park et al. 2002	3.3
OCPs	γ-Chlordane	IADN 1990-2019	3.2
OCPs	Trans-nonachlor	Park et al. 2002	3.8
OCPs	Trans-nonachlor	IADN 1990-2019	3.2
OCPs	Toxaphene	Bidelman & Christensen 1979	5.0
OCPs	Toxaphene	Atlas & Giam1987	4.1
OCPs	Dieldrin	Atlas & Giam1987	3.9
OCPs	Dieldrin	IADN 1990-2019	3.8
OCPs	Endrin	IADN 1990-2019	3.7
OCPs	Endosulfan I	Gioia et al. 2005	3.4
OCPs	Endosulfan I	IADN 1990-2019	4.0
OCPs	Endosulfan II	Gioia et al. 2005	4.8

OCPs	Endosulfan II	IADN 1990-2019	5.0
OCPs	Endosulfansulfate	Gioia et al. 2005	5.4
PCDD/Fs	5-PCDD	Eitzer & Hites 1989	4.0
PCDD/Fs	6-PCDD	Eitzer & Hites 1989	4.0
PCDD/Fs	7-PCDD	Eitzer & Hites 1989	4.8
PCDD/Fs	8-PCDD	Eitzer & Hites 1989	5.0
PCDD/Fs	4-PCDF	Eitzer & Hites 1989	4.3
PCDD/Fs	5-PCDF	Eitzer & Hites 1989	4.1
PCDD/Fs	6-PCDF	Eitzer & Hites 1989	4.0
PCDD/Fs	7-PCDF	Eitzer & Hites 1989	4.5
PCDD/Fs	8-PCDF	Eitzer & Hites 1989	4.3
PAHs	Naphthalene	Park et al. 2002	6.0
PAHs	Naphthalene	He & Balasubramanian 2009	4.8
PAHs	Naphthalene	Zang et al. 2015	4.4
PAHs	Naphthalene	Gonzalez-Gaya 2016	7.2
PAHs	C1-Naphthalene	Park et al. 2002	3.8
PAHs	C1-Naphthalene	Gonzalez-Gaya 2016	9.2
PAHs	C2-Naphthalene	Park et al. 2002	3.5
PAHs	C2-Naphthalene	Gonzalez-Gaya 2016	7.0
PAHs	C3-Naphthalene	Park et al. 2002	3.5
PAHs	C4-Naphthalene	Park et al. 2002	3.4
PAHs	Acenaphthylene	Franz & Eisenreich 1998	2.7
PAHs	Acenaphthylene	Shahpoury 2015	3.7
PAHs	Acenaphthylene	Zang et al. 2015	3.1
PAHs	Acenaphthylene	Gonzalez-Gaya 2016	6.0
PAHs	Acenaphthylene	Li et al. 2016	4.7
PAHs	Acenaphthene	Franz & Eisenreich 1998	3.0
PAHs	Acenaphthene	Park et al. 2002	3.8
PAHs	Acenaphthene	He & Balasubramanian 2009	5.3
PAHs	Acenaphthene	Shahpoury 2015	3.7
PAHs	Acenaphthene	Zang et al. 2015	4.0
PAHs	Acenaphthene	Gonzalez-Gaya 2016	5.2
PAHs	Acenaphthene	Li et al. 2016	5.2
PAHs	Fluorene	Ligocki et al. 1985	3.2
PAHs	Fluorene	Poster & Baker 1996	3.3
PAHs	Fluorene	Franz & Eisenreich 1998	3.1
PAHs	Fluorene	Park et al. 2002	3.4
PAHs	Fluorene	He & Balasubramanian 2009	4.2
PAHs	Fluorene	Shahpoury 2015	3.7
PAHs	Fluorene	Zang et al. 2015	4.1
PAHs	Fluorene	Gonzalez-Gaya 2016	5.2
PAHs	Fluorene	Li et al. 2016	4.5
PAHs	C1-Fluorene	Park et al. 2002	3.9
PAHs	C2-Fluorene	Park et al. 2002	3.2
PAHs	C3-Fluorene	Park et al. 2002	3.5
PAHs	Benzo[b]fluorene	Shahpoury 2015	3.9
PAHs	Benzo[b]fluorene	Li et al. 2016	4.0
PAHs	Dibenzothiophene	Park et al. 2002	3.5

PAHs	Dibenzothiophene	Zang et al. 2015	4.8
PAHs	Dibenzothiophene	Gonzalez-Gaya 2016	4.5
PAHs	C1-Dibenzothiophene	Park et al. 2002	3.2
PAHs	C1-Dibenzothiophene	Gonzalez-Gaya 2016	3.8
PAHs	C2-Dibenzothiophene	Park et al. 2002	3.2
PAHs	C3-Dibenzothiophene	Park et al. 2002	3.3
PAHs	Phenanthrene	Mc Veety & Hites 1988	2.9
PAHs	Phenanthrene	Poster & Baker 1996	3.5
PAHs	Phenanthrene	Franz & Eisenreich 1998	3.5
PAHs	Phenanthrene	Park et al. 2002	3.4
PAHs	Phenanthrene	Sahu et al. 2004	4.5
PAHs	Phenanthrene	He & Balasubramanian 2009	4.0
PAHs	Phenanthrene	Shahpoury 2015	3.7
PAHs	Phenanthrene	Zang et al. 2015	4.3
PAHs	Phenanthrene	Gonzalez-Gaya 2016	3.7
PAHs	Phenanthrene	Li et al. 2016	4.0
PAHs	C1-Phenanthrene	Ligocki et al. 1985	3.4
PAHs	C1-Phenanthrene	Gonzalez-Gaya 2016	3.8
PAHs	C2-Phenanthrene	Gonzalez-Gaya 2016	5.1
PAHs	Phenanthrene/Anthracene	Ligocki et al. 1985	3.5
PAHs	C1-Phenanthrene/Anthracene	Park et al. 2002	3.2
PAHs	C2-Phenanthrene/Anthracene	Park et al. 2002	3.2
PAHs	C3-Phenanthrene/Anthracene	Park et al. 2002	3.0
PAHs	C4-Phenanthrene/Anthracene	Park et al. 2002	3.3
PAHs	Anthracene	Mc Veety & Hites 1988	3.8
PAHs	Anthracene	Poster & Baker 1996	4.8
PAHs	Anthracene	Franz & Eisenreich 1998	3.2
PAHs	Anthracene	Park et al. 2002	3.5
PAHs	Anthracene	Sahu et al. 2004	4.5
PAHs	Anthracene	He & Balasubramanian 2009	3.8
PAHs	Anthracene	Shahpoury 2015	3.4
PAHs	Anthracene	Zang et al. 2015	4.7
PAHs	Anthracene	Gonzalez-Gaya 2016	3.6
PAHs	Anthracene	Li et al. 2016	4.4
PAHs	C1-Fluoranthrene/pyrene	Park et al. 2002	3.9
PAHs	Fluoranthene	Ligocki et al. 1985	3.8
PAHs	Fluoranthene	Mc Veety & Hites 1988	3.7
PAHs	Fluoranthene	Franz & Eisenreich 1998	3.9
PAHs	Fluoranthene	Sahu et al. 2004	4.3
PAHs	Fluoranthene	He & Balasubramanian 2009	3.3
PAHs	Fluoranthene	Shahpoury 2015	4.1
PAHs	Fluoranthene	Zang et al. 2015	4.8
PAHs	Fluoranthene	Gonzalez-Gaya 2016	3.7
PAHs	Fluoranthene	Li et al. 2016	4.1
PAHs	Benzonaphthothiophene	Shahpoury 2015	3.8
PAHs	Pyrene	Ligocki et al. 1985	3.8
PAHs	Pyrene	Mc Veety & Hites 1988	3.5
PAHs	Pyrene	Poster & Baker 1996	3.6

PAHs	Pyrene	Franz & Eisenreich 1998	4.0
PAHs	Pyrene	Sahu et al. 2004	4.3
PAHs	Pyrene	He & Balasubramanian 2009	3.3
PAHs	Pyrene	Shahpoury 2015	4.1
PAHs	Pyrene	Zang et al. 2015	5.0
PAHs	Pyrene	Gonzalez-Gaya 2016	3.9
PAHs	C1-Pyrene	Gonzalez-Gaya 2016	4.1
PAHs	C1-Pyrene	Li et al. 2016	4.3
PAHs	Cyclopenta[cd]pyrene	Shahpoury 2015	5.8
PAHs	Retene	Franz & Eisenreich 1998	4.0
PAHs	Retene	Shahpoury 2015	3.8
PAHs	Retene	Zang et al. 2015	4.1
PAHs	Triphenylene	Shahpoury 2015	4.3
PAHs	Benzo[g,h,i]fluoranthene	Shahpoury 2015	4.1
PAHs	Benzo[g,h,i]fluoranthene	Gonzalez-Gaya 2016	4.7
PAHs	Benzo[a]anthracene	Ligocki et al. 1985	3.6
PAHs	Benzo[a]anthracene	Mc Veety & Hites 1988	4.7
PAHs	Benzo[a]anthracene	Poster & Baker 1996	5.0
PAHs	Benzo[a]anthracene	Franz & Eisenreich 1998	4.0
PAHs	Benzo[a]anthracene	Park et al. 2002	4.7
PAHs	Benzo[a]anthracene	He & Balasubramanian 2009	3.6
PAHs	Benzo[a]anthracene	Shahpoury 2015	3.7
PAHs	Benzo[a]anthracene	Zang et al. 2015	5.6
PAHs	Benzo[a]anthracene	Gonzalez-Gaya 2016	4.7
PAHs	Benzo[a]anthracene	Li et al. 2016	5.1
PAHs	Benzo[a]anthracene/Chrysene	Sahu et al. 2004	4.2
PAHs	Chrysene	Ligocki et al. 1985	3.8
PAHs	Chrysene	Poster & Baker 1996	4.7
PAHs	Chrysene	Franz & Eisenreich 1998	4.0
PAHs	Chrysene	Park et al. 2002	4.6
PAHs	Chrysene	He & Balasubramanian 2009	4.1
PAHs	Chrysene	Shahpoury 2015	4.1
PAHs	Chrysene	Zang et al. 2015	5.4
PAHs	Chrysene	Gonzalez-Gaya 2016	4.3
PAHs	Chrysene	Li et al. 2016	4.6
PAHs	Chrysene/Triphenylene	Mc Veety & Hites 1988	4.5
PAHs	C1-Chrysene	Park et al. 2002	4.2
PAHs	C1-Chrysene	Gonzalez-Gaya 2016	4.7
PAHs	C2-Chrysene	Park et al. 2002	4.0
PAHs	C3-Chrysene	Park et al. 2002	3.8
PAHs	Benzo[b]fluoranthene	Poster & Baker 1996	5.1
PAHs	Benzo[b]fluoranthene	Park et al. 2002	4.9
PAHs	Benzo[b]fluoranthene	He & Balasubramanian 2009	4.5
PAHs	Benzo[b]fluoranthene	Shahpoury 2015	3.9
PAHs	Benzo[b]fluoranthene	Zang et al. 2015	5.2
PAHs	Benzo[k]fluoranthene	Poster & Baker 1996	5.3
PAHs	Benzo[k]fluoranthene	Park et al. 2002	4.8
PAHs	Benzo[k]fluoranthene	Sahu et al. 2004	4.1

PAHs	Benzo[k]fluoranthene	He & Balasubramanian 2009	4.2
PAHs	Benzo[k]fluoranthene	Shahpoury 2015	3.7
PAHs	Benzo[k]fluoranthene	Zang et al. 2015	5.4
PAHs	Benzo[k]fluoranthene	Li et al. 2016	4.2
PAHs	Benzo[b,k]fluoranthene	Franz & Eisenreich 1998	3.9
PAHs	Benzo[b,k]fluoranthene	Gonzalez-Gaya 2016	4.7
PAHs	Benzo[b,j,k]fluoranthene	Ligocki et al. 1985	3.4
PAHs	Benzo[j]fluoranthene	Shahpoury 2015	4.0
PAHs	Benzo[e]pyrene	Ligocki et al. 1985	3.3
PAHs	Benzo[e]pyrene	Mc Veety & Hites 1988	5.1
PAHs	Benzo[e]pyrene	Franz & Eisenreich 1998	3.9
PAHs	Benzo[e]pyrene	Park et al. 2002	4.9
PAHs	Benzo[e]pyrene	Shahpoury 2015	4.1
PAHs	Benzo[e]pyrene	Gonzalez-Gaya 2016	4.5
PAHs	Benzo[a]pyrene	Ligocki et al. 1985	3.2
PAHs	Benzo[a]pyrene	Mc Veety & Hites 1988	5.3
PAHs	Benzo[a]pyrene	Franz & Eisenreich 1998	4.4
PAHs	Benzo[a]pyrene	Park et al. 2002	4.9
PAHs	Benzo[a]pyrene	Sahu et al. 2004	3.9
PAHs	Benzo[a]pyrene	He & Balasubramanian 2009	3.8
PAHs	Benzo[a]pyrene	Shahpoury 2015	3.2
PAHs	Benzo[a]pyrene	Zang et al. 2015	5.5
PAHs	Benzo[a]pyrene	Gonzalez-Gaya 2016	5.1
PAHs	Benzo[a]pyrene	Li et al. 2016	4.6
PAHs	Perylene	Ligocki et al. 1985	3.3
PAHs	Perylene	Park et al. 2002	4.4
PAHs	Perylene	Shahpoury 2015	3.3
PAHs	Perylene	Zang et al. 2015	5.7
PAHs	Perylene	Gonzalez-Gaya 2016	5.4
PAHs	Indeno[1,2,3-cd]pyrene	Mc Veety & Hites 1988	5.2
PAHs	Indeno[1,2,3-cd]pyrene	Poster & Baker 1996	5.4
PAHs	Indeno[1,2,3-cd]pyrene	Franz & Eisenreich 1998	3.7
PAHs	Indeno[1,2,3-cd]pyrene	Park et al. 2002	4.9
PAHs	Indeno[1,2,3-cd]pyrene	He & Balasubramanian 2009	3.8
PAHs	Indeno[1,2,3-cd]pyrene	Shahpoury 2015	3.9
PAHs	Indeno[1,2,3-cd]pyrene	Zang et al. 2015	5.1
PAHs	Indeno[1,2,3-cd]pyrene	Gonzalez-Gaya 2016	5.4
PAHs	Indeno[1,2,3-cd]pyrene	Li et al. 2016	4.2
PAHs	Dibenzo[a,h]anthracene	Franz & Eisenreich 1998	4.1
PAHs	Dibenzo[a,h]anthracene	Park et al. 2002	4.6
PAHs	Dibenzo[a,h]anthracene	He & Balasubramanian 2009	4.6
PAHs	Dibenzo[a,h]anthracene	Zang et al. 2015	5.5
PAHs	Dibenzo[a,h]anthracene	Gonzalez-Gaya 2016	6.2
PAHs	Dibenzo[a,h]anthracene	Li et al. 2016	4.9
PAHs	Benzo[g,h,i]perylene	Ligocki et al. 1985	3.5
PAHs	Benzo[g,h,i]perylene	Mc Veety & Hites 1988	5.4
PAHs	Benzo[g,h,i]perylene	Poster & Baker 1996	4.1
PAHs	Benzo[g,h,i]perylene	Franz & Eisenreich 1998	5.3

PAHs	Benzo[g,h,i]perylene	Park et al. 2002	4.8
PAHs	Benzo[g,h,i]perylene	He & Balasubramanian 2009	4.2
PAHs	Benzo[g,h,i]perylene	Shahpoury 2015	3.8
PAHs	Benzo[g,h,i]perylene	Zang et al. 2015	5.3
PAHs	Benzo[g,h,i]perylene	Gonzalez-Gaya 2016	5.4
PAHs	Benzo[g,h,i]perylene	Li et al. 2016	4.1
PAHs	Coronene	Ligocki et al. 1985	3.8
PAHs	Coronene	Shahpoury 2015	3.6
PAHs	ΣPAHs	Offenberg & Baker 2002	5.7
PAHs	ΣPAHs	Holoubek et al. 2007	4.1
PAHs	ΣPAHs	Li et al. 2016	4.3
PAHs	Dibenzofuran	Ligocki et al. 1985	3.0
PAHs	9-Fluorenone	Ligocki et al. 1985	4.0
PAHs	9,10-Anthracenedione	Ligocki et al. 1985	4.3
PBDEs	BDE-3	Noël et al. 2009	5.3
PBDEs	BDE-28	TerSchure et al. 2004a	4.9
PBDEs	BDE-28	Mariani et al. 2008	3.5
PBDEs	BDE-28	Zhang et al. 2009	2.8
PBDEs	BDE-47	TerSchure et al. 2004a	5.1
PBDEs	BDE-47	Venier & Hites 2008	5.0
PBDEs	BDE-47	Mariani et al. 2008	3.3
PBDEs	BDE-47	Noël et al. 2009	4.3
PBDEs	BDE-47	Zhang et al. 2009	3.1
PBDEs	BDE-47	Guo et al. 2014	4.4
PBDEs	BDE-66	Guo et al. 2014	4.0
PBDEs	BDE-77	Guo et al. 2014	3.6
PBDEs	BDE-99	TerSchure et al. 2004a	5.5
PBDEs	BDE-99	Mariani et al. 2008	4.4
PBDEs	BDE-99	Noël et al. 2009	4.2
PBDEs	BDE-99	Zhang et al. 2009	3.1
PBDEs	BDE-99	Guo et al. 2014	4.2
PBDEs	BDE-100	Mariani et al. 2008	4.1
PBDEs	BDE-100	Zhang et al. 2009	3.2
PBDEs	BDE-126	Guo et al. 2014	5.2
PBDEs	BDE-138	Guo et al. 2014	4.7
PBDEs	BDE-153	TerSchure et al. 2004a	5.8
PBDEs	BDE-153	Mariani et al. 2008	5.2
PBDEs	BDE-153	Zhang et al. 2009	3.2
PBDEs	BDE-153	Guo et al. 2014	4.7
PBDEs	BDE-154	Mariani et al. 2008	5.0
PBDEs	BDE-154	Zhang et al. 2009	3.4
PBDEs	BDE-154	Guo et al. 2014	4.7
PBDEs	BDE-181	Guo et al. 2014	5.0
PBDEs	BDE-183	TerSchure et al. 2004b	5.5
PBDEs	BDE-183	Mariani et al. 2008	5.4
PBDEs	BDE-183	Guo et al. 2014	4.7
PBDEs	BDE-190	Guo et al. 2014	4.5
PBDEs	BDE-196	Guo et al. 2014	4.5

PBDEs	BDE-203	Guo et al. 2014	4.5
PBDEs	BDE-204	Guo et al. 2014	4.4
PBDEs	BDE-206	Guo et al. 2014	4.2
PBDEs	BDE-207	Noël et al. 2009	4.7
PBDEs	BDE-207	Guo et al. 2014	4.3
PBDEs	BDE-208	Guo et al. 2014	4.3
PBDEs	BDE-209	TerSchure et al. 2004a	6.1
PBDEs	BDE-209	TerSchure et al. 2004b	6.0
PBDEs	BDE-209	Venier & Hites 2008	5.1
PBDEs	BDE-209	Mariani et al. 2008	5.1
PBDEs	BDE-209	Noël et al. 2009	5.9
PBDEs	BDE-209	Zhang et al. 2009	3.5
PBDEs	BDE-209	Guo et al. 2014	4.6
PBDEs	ΣBDE	TerSchure et al. 2004a	5.8
PBDEs	ΣBDE	TerSchure et al. 2004b	5.7
PBDEs	ΣBDE	Venier & Hites 2008	5.2
PBDEs	ΣBDE	Mariani et al. 2008	4.1
PBDEs	ΣBDE	Noël et al. 2009	5.0
OPEs	TiBP	Zhang et al. 2020	6.2
OPEs	TNBP	Zhang et al. 2020	5.5
OPEs	TBOEP	Zhang et al. 2020	5.1
OPEs	TCEP	Zhang et al. 2020	5.0
OPEs	TDCIPP	Zhang et al. 2020	5.3
PFAS	MeFOSE	Dreyer et al. 2010	5.3
PFAS	EtFOSE	Dreyer et al. 2010	5.2
PFAS	MeFBSE	Dreyer et al. 2010	5.3

Table S8. Dimension-less Henry's Law constant values and their sources used for the meta-analysis of $K_{RG}H'$.

Family	Compound	H' (25°C)	Reference
PAHs	2-Methylnaphthalene	0.020732	Bamford et al. 1999[6]
	1-Methylnaphthalene	0.020424	
	Acenaphthylene	0.004970	
	Acenaphthene	0.007136	
	Fluorene	0.004101	
	Phenanthrene	0.001773	
	Anthracene	0.002169	
	1-Methylphenanthrene	0.001979	
	Fluoranthene	0.000749	
	Pyrene	0.000742	
	Benzo[a]fluorene	0.001087	
	Benz[a]anthracene	0.000468	
	Chrysene	0.000209	
PCBs	Biphenyl	0.012593	Mackay Shiu & 2006[7]
	PCB- 8	0.010869	Bamford et al. 1999[6]
	PCB- 11	0.007098	
	PCB-18	0.011115	
	PCB- 28	0.007479	
	PCB-31	0.010950	
	PCB-52	0.016766	
	PCB-44	0.011367	
	PCB-66	0.011282	
	PCB-101	0.025103	
	PCB-105	0.014990	
	PCB-138	0.021783	
	PCB-153	0.022951	
	PCB-180	0.018901	
	PCB-187	0.021300	
	PCB-195	0.008002	
	PCB- 6	0.010788	
	PCB-12	0.007098	
	PCB-13	0.007152	
	PCB-24	0.024547	
	PCB-27	0.007314	
	PCB-16	0.010788	
	PCB-32	0.007314	
	PCB-31	0.010950	
	PCB-33	0.024364	
	PCB-22	0.016395	
	PCB-47	0.007592	
	PCB-48	0.017018	
	PCB-41	0.007592	
	PCB-46	0.016892	

	PCB-66	0.011282	
	PCB-91	0.019925	
	PCB-101	0.025103	
	PCB-90	0.011282	
	PCB-174	0.030494	
	PCB-196	0.008002	
	PCB-203	0.008002	
	PCB-194	0.003570	
	PCB-26	0.010950	
	PCB-4	0.010708	
	PCB-15	0.007098	
	PCB-40	0.007535	
	PCB-81	0.007479	
	PCB-82	0.024364	
	PCB-127	0.006346	
	PCB-194	0.003570	
	PCB-205	0.003570	
	PCB-5+8	0.010869	
	PCB-6	0.010788	
	PCB-16+39	0.007098	
	PCB-17	0.024547	
	PCB-24+27	0.007314	
	PCB-49	0.017018	
	PCB-70	0.011282	
	PCB-74	0.017018	
	PCB-95	0.025671	
	PCB-99	0.017404	
	PCB-118	0.010708	
	PCB-128+169	0.018073	
	PCB-132	0.011538	
	PCB-177	0.013705	
	PCB-149	0.016152	
	PCB-158+160	0.033104	
	PCB-170+190	0.008369	
	PCB-177	0.013705	
PBDEs	BDE-28	0.002899	Cetin & Odabasi 2005[8]
	BDE-47	0.000361	
	BDE-100	0.000170	
	BDE-99	0.000298	
	BDE-154	0.000055	
	BDE-153	0.000081	
	BDE-209	0.000016	
PCDD/Fs	4-PCDF	0.000605	Eitzer & Hites 1989[9]
	5-PCDF	0.001076	
	6-PCDF	0.001126	
	7-PCDF	0.000167	
	8-PCDF	0.000069	
	5-PCDD	0.001555	

	6-PCDD	0.001745	
	7-PCDD	0.000036	
	8-PCDD	0.000004	
PFAS	MeFBSE	0.120337	Xie et al, 2013[10]
	EtFOSE	34.75	
	MeFOSE	51.94	
OPEs	TEP	0.000025	van der Veen & Boer 2012[11]
	TNBP	0.000126	
	TBOEP	0.000000	
	TCEP	0.000001	
	TDCIPP	0.000000	
	TiBP	0.000130	EPISuite
OCPs	a-HCH	0.000299	Xiao et al. 2004[12]
	b-HCh	0.000015	
	y-HCh	0.000125	
	Hexachlorobenzene (HCB)	0.026235	Peterson & Batley 1993[13]
	p,p′-DDT	0.000444	
	Chlordane (CC)	0.002301	
	a-Endosulfan	0.000283	
	b-Endosulfan	0.000018	
	endosulfan sulfate	0.015000	Hinckley et al. 1990[14]

Table S9. PFAS concentrations in rain (pg L^{-1}) and aerosols (pg m^{-3}) samples from Livingston and Deception Island. *= Data from Casas et al. 2020.i Date = start of the rain event or start of aerosol sampling. f Date = end of the rain event or end of aerosol sampling.

Type	Location	Sample	i Date	f Date	PFBS	PFOS	PFBA	PFHxA	PFHpA	PFOA	PFNA	PFDA	PFUnDA	PFDoDA	PFTrDA	PFTeDA	∑PFAs
Aerosol	Deception Island	A01	23/01/2017	25/01/2017	n.d.	0.00042	0.08	0.0027	<LOD	0.003	0.00052	0.0012	0.00017	0.00027	0.00007	0.00010	0.09
Aerosol	Deception Island	A02	25/01/2017	27/01/2017	0.013	0.0014	0.28	0.0048	0.0036	0.016	0.0016	0.0029	0.00021	0.00057	n.d.	n.d.	0.32
Aerosol	Deception Island	A03	27/01/2017	29/01/2017	0.016	0.0014	0.17	0.010	0.0030	0.010	0.0011	0.0017	0.00021	0.00021	n.d.	n.d.	0.22
Aerosol	Deception Island	A04	31/01/2017	02/02/2017	0.001	0.00010	0.091	0.0018	<LOD	0.002	0.00021	0.0005	0.00008	0.00010	n.d.	n.d.	0.10
Aerosol	Deception Island	A05	08/02/2017	10/02/2017	0.0035	0.00012	0.046	0.0022	0.0013	0.002	0.00032	0.0007	0.00012	0.00010	n.d.	n.d.	0.056
Aerosol	Deception Island	A06	10/02/2017	12/02/2017	0.018	0.00039	0.044	0.0027	<LOD	0.003	0.00039	0.00089	0.00020	n.d.	n.d.	n.d.	0.071
Aerosol	Deception Island	A07	12/02/2017	14/02/2017	0.014	0.00080	0.073	0.0063	0.0020	0.008	0.0010	0.0013	0.00016	0.00013	n.d.	n.d.	0.11
Aerosol	Deception Island	A08	14/02/2017	16/02/2017	0.0072	0.00032	0.041	0.0027	0.0011	0.003	0.00032	0.00050	0.00011	<LOD	n.d.	n.d.	0.056
Aerosol	Livingston Island	A10	02/01/2018	05/01/2018	0.0008	0.00014	0.031	0.0012	<LOD	0.0017	0.00045	0.00073	0.00014	<LOD	n.d.	n.d.	0.036
Aerosol	Livingston Island	A11	07/01/2018	09/01/2018	0.0014	0.00013	0.033	0.0011	<LOD	0.0015	0.00034	0.00063	0.00008	<LOD	n.d.	n.d.	0.038
Aerosol	Livingston Island	A12	09/01/2018	11/01/2018	0.0007	0.00013	0.033	<LOD	<LOD	0.0012	0.00023	0.00039	0.00005	<LOD	n.d.	n.d.	0.036
Aerosol	Livingston Island	A13	11/01/2018	13/01/2018	0.018	0.0021	0.45	0.083	0.015	0.056	0.0042	0.0051	0.000563	<LOD	n.d.	n.d.	0.63
Aerosol	Livingston Island	A14	18/01/2018	20/01/2018	<LOD	<LOD	0.007	n.d.	<LOD	<LOD	<LOD	<LOD	0.000053	n.d.	n.d.	n.d.	0.007
Aerosol	Livingston Island	A15	20/01/2018	22/01/2018	0.0016	0.00005	0.018	0.0009	0.0017	0.0021	0.0003	0.00047	0.000052	n.d.	n.d.	n.d.	0.025
Aerosol	Livingston Island	A16	30/01/2018	02/02/2018	0.0017	0.00005	0.035	0.0012	<LOD	0.0017	n.d.	0.00050	0.00010	n.d.	n.d.	n.d.	0.041
Aerosol	Livingston Island	A17*	02/02/2018	04/02/2018	0.012	n.d.	0.41	0.023	0.021	0.029	n.d.	0.010	0.0026	n.d.	n.d.	n.d.	0.50
Aerosol	Livingston Island	A18*	06/02/2018	08/02/2018	0.015	0.0249	0.53	0.029	0.031	0.041	0.018	0.013	0.0044	n.d.	n.d.	n.d.	0.71
Aerosol	Livingston Island	A19	24/02/2018	26/02/2018	0.0015	0.00008	0.024	0.0010	<LOD	0.0015	0.00026	0.00031	0.000078	n.d.	n.d.	n.d.	0.029
Aerosol	Livingston Island	A20	26/02/2018	03/01/2018	0.0013	0.00005	0.02	<LOD	<LOD	0.0011	n.d.	0.00013	0.000052	n.d.	n.d.	n.d.	0.019
Rain	Deception Island	R01	27/01/2017	28/01/2017	180	41	3000	64	200	452	260	230	71	79	21	32	4700
Rain	Deception Island	R02	01/02/2017	01/02/2017	97	27	1600	29	63	191	171	150	64	85	15	39	2500
Rain	Deception Island	R03	09/02/2017	09/02/2017	190	22	3300	150	220	434	414	230	88	62	14	19	5200
Rain	Deception Island	R04	10/02/2017	10/02/2017	33	310	1600	41	34	557	76	52	15	15	5.0	98	2900
Rain	Deception Island	R05	11/02/2017	12/02/2017	60	17	2200	84	99	195	128	120	44	38	5.5	4.0	3000
Rain	Deception Island	R06	12/02/2017	13/02/2017	57	21	1600	65	60	164	104	110	41	41	7.0	8.0	2200

Rain	Deception Island	R07	13/02/2017	14/02/2017	20	n.d.	450	19	18	76	31	24	8.0	8.5	0.50	2.0	660
Rain	Deception Island	R08	14/02/2017	14/02/2017	220	9.0	5500	330	330	450	302	280	86	62	7.0	4.5	7600
Rain	Deception Island	R09	15/02/2017	17/02/2017	330	8.0	3300	86	61	164	81	86	20	19	1.5	3.5	4200
Rain	Deception Island	R10	09/01/2018	09/01/2018	92	5.0	2600	250	230	357	53	30	5.5	6.5	0.5	1.5	3700
Rain	Deception Island	R12	11/01/2018	11/01/2018	160	13	6600	360	380	531	206	140	35	31	4.5	6.5	8400
Rain	Livingston Island	R13	13/01/2018	13/01/2018	38	3.5	1700	n.d.	54	198	37	29	8.5	8.5	n.d.	1.5	2100
Rain	Livingston Island	R14	19/01/2018	19/01/2018	240	4.0	2100	140	150	241	108	120	31	26	3.5	4.0	3100
Rain	Livingston Island	R15	22/01/2018	22/01/2018	120	2.5	1900	100	140	103	64	60	16	15	2.0	2.0	2500
Rain	Livingston Island	R16	22/01/2018	22/01/2018	211	2.5	4000	320	350	270	144	120	37	31	5.5	2.0	5500
Rain	Livingston Island	R17	22/01/2018	22/01/2018	130	3.5	4000	290	310	375	175	140	38	27	3.0	n.d.	5100
Rain	Livingston Island	R18	01/02/2018	01/02/2018	5	2.0	330	n.d.	13	20	12	11	3.5	2.5	n.d.	n.d.	400
Rain	Livingston Island	R19	04/02/2018	04/02/2018	79	5.5	2900	150	250	400	147	110	51	49	15	14	4200
Rain	Livingston Island	R20	09/02/2018	09/02/2018	35	2.0	n.d.	83	210	160	54	27	6.5	5.5	1.0	n.d.	580
Rain	Livingston Island	R21	27/02/2018	27/02/2018	60	5.5	2900	110	200	180	88	70	28	25	4.0	1.0	3600

Table S10. OPE concentrations in rain (pg L^{-1}) and aerosols (pg m^{-3}) samples from Livingston Island. i Date = start of the rain event or start of aerosol sampling. f Date = end of the rain event or end of aerosol sampling.

Type	i Date	f Date	Sample	TCEP	TCPP-2	TCPP-3	TCPP-1	TDCIPP	TEP	TiBP	TNBP	EHDPP	TEHP	TPHP	TPrP	∑OPE
Aerosols	02/01/2018	05/01/2018	A01	<LOD	2.5	0.18	10.4	0.36	0.08	13	28	<LOD	1.4	0.51	n.d.	56
Aerosols	07/01/2018	09/01/2018	A02	2.6	<LOD	<LOD	<LOD	0.23	<LOD	10	22	<LOD	0.81	1.0	0.048	37
Aerosols	09/01/2018	11/01/2018	A03	<LOD	1.84	<LOD	8.7	0.21	<LOD	9.0	16	<LOD	0.49	0.45	<LOD	37
Aerosols	30/01/2018	02/02/2018	A04	<LOD	2.23	<LOD	12	0.26	<LOD	2.9	14	<LOD	0.67	<LOD	n.d.	32
Aerosols	24/02/2018	26/02/2018	A05	<LOD	8.00	1.3	41	0.34	<LOD	3.9	5.1	0.74	0.45	0.047	0.040	61
Aerosols	26/02/2018	03/01/2018	A06	<LOD	2.11	0.11	11	<LOD	<LOD	2.5	11	1.2	0.91	<LOD	0.065	28
Rain	05/01/2018	05/01/2018	R01	11000	4200	402	18000	7600	230	5400	5900	870	<LOD	<LOD	44	54000
Rain	09/01/2017	09/01/2017	R02	2900	500	<LOD	2400	530	560	1600	1200	49	<LOD	<LOD	<LOD	9700
Rain	13/01/2018	13/01/2018	R03	3300	1200	76	5600	<LOD	810	1900	<LOD	<LOD	<LOD	<LOD	<LOD	13000
Rain	19/01/2018	19/01/2018	R04	1800	1200	91	6700	2400	490	840	790	<LOD	<LOD	<LOD	<LOD	14000
Rain	22/01/2018	22/01/2018	R05	2600	2200	140	12000	240	390	530	770	64	<LOD	<LOD	<LOD	19000
Rain	22/01/2018	22/01/2018	R06	2700	3800	270	22000	5300	2000	1700	760	<LOD	<LOD	<LOD	<LOD	39000
Rain	22/01/2018	22/01/2018	R07	2600	3700	250	23000	<LOD	2200	1000	<LOD	<LOD	<LOD	<LOD	<LOD	32000
Rain	28/01/2018	28/01/2018	R08	1900	12000	580	69000	300	8300	1700	370	16	<LOD	<LOD	<LOD	94000
Rain	28/01/2018	28/01/2018	R09	1800	8700	480	52000	2700	5900	1100	390	63	<LOD	<LOD	<LOD	74000
Rain	09/02/2018	09/02/2018	R10	<LOD	1500	110	8200	<LOD	490	710	<LOD	37	<LOD	<LOD	n.d.	11000

Table S11. PAH concentrations in rain (pg L^{-1}) and aerosol (pg m^{-3}) samples from Livingston Island. i Date = start of the rain event or start of aerosol sampling. f Date = end of the rain event or end of aerosol sampling.

Type	Sample	i Date	f Date	Naphthalene	C1-Naphtalene	C2-Naphtalene	Acenaphthylene	C3-Naphtalene	Fluorene	C1-Fluorene	Phenanthrene
Aerosols	A01	02/01/2018	05/01/2018	<LOD	<LOD	<LOD	<LOD	<LOD	<LOD	<LOD	<LOD
Aerosols	A02	07/01/2018	09/01/2018	<LOD	<LOD	<LOD	<LOD	<LOD	<LOD	<LOD	<LOD
Aerosols	A03	09/01/2018	11/01/2018	<LOD	<LOD	<LOD	<LOD	<LOD	<LOD	<LOD	<LOD
Aerosols	A04	18/01/2018	20/01/2018	<LOD	<LOD	0.71	<LOD	<LOD	<LOD	<LOD	2.2
Aerosols	A05	20/01/2018	22/01/2018	<LOD	<LOD	0.24	<LOD	<LOD	<LOD	<LOD	<LOD
Aerosols	A06	26/02/2018	03/03/2018	<LOD	<LOD	<LOD	<LOD	<LOD	0.11	0.08	0.52
Rain	R01	05/01/2018	05/01/2018	<LOD	650	6000	<LOD	3400	<LOD	2700	1500
Rain	R02	09/01/2017	09/01/2017	<LOD	<LOD	1900	920	1300	200	330	860
Rain	R03	13/01/2018	13/01/2018	1100	1200	3000	440	1400	260	930	1600
Rain	R04	19/01/2018	19/01/2018	980	870	1200	140	710	210	260	780
Rain	R05	22/01/2018	22/01/2018	<LOD	<LOD	650	180	430	150	170	890
Rain	R06	22/01/2018	22/01/2018	<LOD	780	4600	220	1200	250	1700	940
Rain	R07	22/01/2018	22/01/2018	1100	740	1500	260	620	170	250	650
Rain	R08	28/01/2018	28/01/2018	<LOD	530	2200	170	590	250	930	1300
Rain	R09	28/01/2018	28/01/2018	2900	2400	5600	210	3100	590	600	2600
Rain	R10	09/02/2018	09/02/2018	<LOD	<LOD	110	96	<LOD	<LOD	160	870

Type	Sample	i Date	f Date	C1-Dibenzothiophene	C1-Phenanthrene	C2-Dibenzothiphene	C2-Phenanthrene-10-	Fluoranthene	Pyrene	C3-Phenanthrene	C1-Fluoranthrene/Pyrene
Aerosols	A01	02/01/2018	05/01/2018	0.028	1.2	<LOD	7.3	1.0	<LOD	8.6	2.3
Aerosols	A02	07/01/2018	09/01/2018	0.022	1.8	0.08	7.6	1.1	0.95	9.1	2.9
Aerosols	A03	09/01/2018	11/01/2018	0.0034	0.47	<LOD	1.9	0.40	<LOD	2.3	0.81
Aerosols	A04	18/01/2018	20/01/2018	<LOD	0.40	0.80	<LOD	<LOD	<LOD	0.0049	<LOD
Aerosols	A05	20/01/2018	22/01/2018	<LOD	0.33	0.60	<LOD	<LOD	<LOD	<LOD	<LOD
Aerosols	A06	26/02/2018	03/03/2018	0.01	0.68	0.05	2.1	0.75	0.56	3.10	1.4
Rain	R01	05/01/2018	05/01/2018	480	<LOD	140	530	94	98	350	51
Rain	R02	09/01/2017	09/01/2017	200	640	130	390	100	93	150	95
Rain	R03	13/01/2018	13/01/2018	140	600	69	420	110	90	110	69
Rain	R04	19/01/2018	19/01/2018	110	280	<LOD	120	<LOD	80	52	<LOD
Rain	R05	22/01/2018	22/01/2018	130	300	19	150	97	81	46	18
Rain	R06	22/01/2018	22/01/2018	240	1100	80	2300	<LOD	75	180	29
Rain	R07	22/01/2018	22/01/2018	190	240	18	79	<LOD	78	53	<LOD
Rain	R08	28/01/2018	28/01/2018	180	630	64	360	140	110	73	<LOD
Rain	R09	28/01/2018	28/01/2018	160	1200	10	550	200	190	150	16
Rain	R10	09/02/2018	09/02/2018	20	350	2.7	280	110	103	100	<LOD

Type	Sample	i Date	f Date	C2-pyrene-8-	Benzo[g,h,i]fluoranthrene	Benzo[a]anthracene	Chrysene	C1-Chrysene	Benzo[k+j]fluoranthene	Perylene	∑PAHs
Aerosols	A01	02/01/2018	05/01/2018	1.3	3.0	0.71	<LOD	1.0	0.18	<LOD	27
Aerosols	A02	07/01/2018	09/01/2018	1.6	2.7	0.62	1.2	1.8	0.31	0.31	32
Aerosols	A03	09/01/2018	11/01/2018	0.29	1.0	<LOD	<LOD	0.57	0.12	0.18	8
Aerosols	A04	18/01/2018	20/01/2018	<LOD	6.2	<LOD	<LOD	<LOD	<LOD	<LOD	10
Aerosols	A05	20/01/2018	22/01/2018	<LOD	3.8	<LOD	<LOD	<LOD	<LOD	<LOD	5
Aerosols	A06	26/02/2018	03/03/2018	0.46	3.5	0.36	1.7	0.81	0.26	0.26	17
Rain	R01	05/01/2018	05/01/2018	170	701.3	150	<LOD	78	<LOD	<LOD	17000
Rain	R02	09/01/2017	09/01/2017	100	208.8	<LOD	120	67	<LOD	<LOD	7800
Rain	R03	13/01/2018	13/01/2018	85	<LOD	<LOD	<LOD	110	<LOD	<LOD	12000
Rain	R04	19/01/2018	19/01/2018	47	<LOD	<LOD	<LOD	<LOD	<LOD	<LOD	5800
Rain	R05	22/01/2018	22/01/2018	73	<LOD	<LOD	<LOD	<LOD	<LOD	<LOD	3400
Rain	R06	22/01/2018	22/01/2018	75	<LOD	<LOD	<LOD	<LOD	<LOD	<LOD	14000
Rain	R07	22/01/2018	22/01/2018	43	<LOD	<LOD	<LOD	<LOD	<LOD	<LOD	6000
Rain	R08	28/01/2018	28/01/2018	68	159.55	<LOD	<LOD	<LOD	<LOD	<LOD	7800
Rain	R09	28/01/2018	28/01/2018	100	201.25	<LOD	<LOD	47	<LOD	<LOD	21000
Rain	R10	09/02/2018	09/02/2018	94.05	<LOD	<LOD	<LOD	55	<LOD	<LOD	2400

Table S12. Pearson's correlations between log K_{RA}, log K_{RG}, log K_{RP} and physical chemical properties (log K_{aw}, log K_{oa}, log K_{ow}).

		Kra						
		**OCPs**	**OPEs**	**PAHs**	**PBDEs**	**PCBs**	**PCDDFs**	**PFAS**
**Kow**	**R**	-0.6	0.4	0.26	0.28	0.38	0.41	-0.87
	**p**	0.0022	0.0031	<2.26e-16	0.026	0.00013	0.36	0.32
	**equation**	logkra=6.2-0.52logkow	logkra=4.4+0.32logkow	logkra=3.2+0.3logkow	logkra=3.2+0.17logkow	logkra=0.89+0.45logkow	logkra=2.6+0.22logkow	logkra=5.8-0.15logkow
**Koa**	**R**	-0.7	-0.29	0.23	0.43	0.39	0.82	-0.067
	**p**	0.00013	0.036	5.20E-13	0.0021	5.90E-05	0.023	0.96
	**equation**	logkra=11-0.8logkoa	logkra=6.3-0.099logkoa	logkra=3.3+0.18logkoa	logkra=2.1+0.21logkoa	logkra=-0.54+0.52logkoa	logkra=1.6+0.25logkoa	logkra=5.3-0.0019logkoa
**Kaw**	**R**	-0.27	0.44	-0.13	-0.43	-0.002	-0.95	-0.071
	**p**	0.0061	0.00083	3.30E-05	0.0025	0.97	9.20E-05	0.95
	**equation**	logkra=2.7-0.26logkaw	logkra=6.3-0.15logkaw	logkra=3.9-0.29logkaw	logkra=2-0.62logkaw	logkra=3.5-0.007logkaw	logkra=3-0.38logkaw	logkra=5.3-0.0018ogkaw

		Krg						
		**OCPs**	**OPEs**	**PAHs**	**PBDEs**	**PCBs**	**PCDDFs**	**PFAS**
**Kow**	**R**	-8.2		0.21	0.47	0.49	0.47	-0.44
	**p**	<2.2e-16		1.00E-04	0.0027	3.50E-07	0.03	0.71
	**equation**	logkrg=8.2-logkow		logkrg=2.9+0.28logkow	logkrg=0.24+0.54logkow	logkrg=2.7+0.24logkow	logkrg=1.2+0.42logkow	logkrg=5.7-0.1logkow
**Koa**	**R**	-0.86		0.13	0.66	0.5	0.82	-0.62
	**p**	<2.2e-16		0.015	0.00013	2.20E-07	5.10E-06	0.57
	**equation**	logkrg=18-1.5logkoa		logkrg=3.5-0.14logkoa	logkrg=1.6-0.31logkoa	logkrg=-1.3+0.61logkoa	logkrg=0.018+0.41logkoa	logkrg=5.4-0.023logkoa
**Kaw**	**R**	-0.2		-0.12	-0.7	0.024	-0.96	0.51
	**p**	0.0012		0.008	3.80E-05	0.66	5.80E-15	0.66
	**equation**	logkrg=2.5-0.26logkaw		logkrg=3.5-0.33logkaw	logkrg=0.75-1.1logkaw	logkrg=3.6+0.087logkaw	logkrg=1.6-0.84logkaw	logkrg=5.3+0.017logkaw

		Krp						
		**OCPs**	**OPEs**	**PAHs**	**PBDEs**	**PCBs**	**PCDDFs**	**PFAS**
**Kow**	**R**	-0.36	-0.64	-0.4	0.061	-0.082	0.0096	0.31
	**p**	0.38	5.10E-05	<2.2e-16	0.69	0.59	0.97	1.40E-05
	**equation**	logkrp=6.3-0.3logkow	logkrp=7.4-0.47logkow	logkrp=9-0.64logkow	logkrp=4.8+0.024logkow	logkrp=5.9-0.081logkow	logkrp=4.4+0.0048logkow	logkrp=5.8+0.15logkow
**Koa**	**R**	-0.54	-0.059	-0.45	0.17	-0.081	0.45	
	**p**	0.17	0.74	<2.2e-16	0.4	0.6	0.042	
	**equation**	logkrp=11-0.62logkoa	logkrp=6.1-0.036logkoa	logkrp=9.6-0.49logkoa	logkrp=4.5+0.051logkoa	logkrp=6.1-0.086logkoa	logkrp=3.1+0.12logkoa	
**Kaw**	**R**	-0.042	-0.65	0.49	-0.14	0.14	-0.69	
	**p**	0.73	4.50E-05	<2.2e-16	0.48	0.19	5.80E-05	
	**equation**	logkrp=4.9-0.033logkoa	logkrp=3-0.54logkoa	logkrp=9.5+1.4logkoa	logkrp=4.5-0.15logkoa	logkrp=6.3+0.49logkoa	logkrp=3.6-0.24logkoa	

References

(1) Casas, G.; Martínez-Varela, A.; Roscales, J. L.; Vila-Costa, M.; Dachs, J.; Jiménez, B. Enrichment of Perfluoroalkyl Substances in the Sea-Surface Microlayer and Sea-Spray Aerosols in the Southern Ocean. Environ. Pollut. **2020,** 267, 115512.

(2) González-Gaya, B.; Casal, P.; Jurado, E.; Dachs, J.; Jiménez, B. Vertical Transport and Sinks of Perfluoroalkyl Substances in the Global Open Ocean. Environ. Sci. Process. Impacts **2019**, 21, 1957–1969.

(3) Sun, Y.; De Silva, A. O.; St Pierre, K. A.; Muir, D. C. G.; Spencer, C.; Lehnherr, I.; MacInnis, J. J. Glacial Melt Inputs of Organophosphate Ester Flame Retardants to the Largest High Arctic Lake. Environ. Sci. Technol. **2020**, 54, 2734–2743.

(4) Zhong, M.; Tang, J.; Guo, X.; Guo, C.; Li, F.; Wu, H. Occurrence and Spatial Distribution of Organophosphorus Flame Retardants and Plasticizers in the Bohai, Yellow and East China Seas. Sci. Total Environ. **2020**, 741, 140434.

(5) Castro-Jiménez, J.; González-Gaya, B.; Pizarro, M.; Casal, P.; Pizarro-Álvarez, C.; Dachs, J. Organophosphate Ester Flame Retardants and Plasticizers in the Global Oceanic Atmosphere. Environ. Sci. Technol. **2016**, 50, 12831–12839.

(6) Bamford, H. A.; Poster, Di. L.; Baker, J. E. Temperature Dependence of Henry's Law Constants of Thirteen Polycyclic Aromatic Hydrocarbons between 4°C AND 31°C. Environ. Toxicol. Chem. **1999**, 18, 1905–1912.

(7) Mackay, D.; Shiu, W. Y.; Lee, S. C. Handbook of physical-chemical properties and environmental fate for organic chemicals. **2006**.CRC press.

(8) Cetin, B.; Odabasi, M. Measurement of Henry's Law Constants of Seven Polybrominated Diphenyl Ether (PBDE) Congeners as a Function of Temperature. Atmos. Environ. **2005**, 39, 5273–5280.

(9) Eltzert, B. D.; Hites, R. A. Atmospheric Transport and Deposition of Polychlorinated Dibenzo-p-Dioxins and Dibenzofurans. Environ. Sci. Technol. **1989**, *23*, 1396-1401.

(10) Xie, Z.; Zhao, Z.; Möller, A.; Wolschke, H.; Ahrens, L.; Sturm, R.; Ebinghaus, R. Neutral Poly- and Perfluoroalkyl Substances in Air and Seawater of the North Sea. Environ. Sci. Pollut. Res. **2013**, 20, 7988–8000.

(11) van der Veen, I.; de Boer, J. Phosphorus Flame Retardants: Properties, Production, Environmental Occurrence, Toxicity and Analysis. Chemosphere. **2012**, 88, 1119–1153.

(12) Xiao, H.; Li, N.; Wania, F. Compilation, Evaluation, and Selection of Physical-Chemical Property Data for α-, β-, and γ-Hexachlorocyclohexane. Journal of Chemical & Engineering Data. **2004**, 49, 173-185.

(13) Peterson, S. M.; Batley, G. E. The Fate of Endosulfan in Aquatic Ecosystems. Environ. Pollut. **1993**, 82, 143–152.

(14) Hinckley, D. A.; Bidleman, T. F.; Foreman, W. T.; Tuschall, J. R. Determination of Vapor Pressures for Nonpolar and Semipolar Organic Compounds from Gas Chromatographic Retention Data. Journal of Chemical and Engineering Data.**1990**, 35, 232-237.
